# Systemically targeted cancer immunotherapy and gene delivery using transmorphic particles

**DOI:** 10.15252/emmm.202115418

**Published:** 2022-06-27

**Authors:** Paladd Asavarut, Sajee Waramit, Keittisak Suwan, Gert J K Marais, Aitthiphon Chongchai, Surachet Benjathummarak, Mariam Al‐Bahrani, Paula Vila‐Gomez, Matthew Williams, Prachya Kongtawelert, Teerapong Yata, Amin Hajitou

**Affiliations:** ^1^ Cancer Phagotherapy, Department of Brain Sciences Imperial College London London UK; ^2^ Thailand Excellence Centre for Tissue Engineering and Stem Cells, Faculty of Medicine Chiang Mai University Chiang Mai Thailand; ^3^ Center of Excellence for Antibody Research, Faculty of Tropical Medicine Mahidol University Bangkok Thailand; ^4^ Department of Surgery and Cancer Imperial College London London UK; ^5^ Present address: Department of Physiology Chulalongkorn University Bangkok Thailand

**Keywords:** cancer immunotherapy, cytokines, targeted gene delivery, vector development, bacteriophage, Cancer, Immunology

## Abstract

Immunotherapy is a powerful tool for cancer treatment, but the pleiotropic nature of cytokines and immunological agents strongly limits clinical translation and safety. To address this unmet need, we designed and characterised a systemically targeted cytokine gene delivery system through transmorphic encapsidation of human recombinant adeno‐associated virus DNA using coat proteins from a tumour‐targeted bacteriophage (phage). We show that Transmorphic Phage/AAV (TPA) particles provide superior delivery of transgenes over current phage‐derived vectors through greater diffusion across the extracellular space and improved intracellular trafficking. We used TPA to target the delivery of cytokine‐encoding transgenes for interleukin‐12 (IL12), and novel isoforms of IL15 and tumour necrosis factor alpha (TNFα) for tumour immunotherapy. Our results demonstrate selective and efficient gene delivery and immunotherapy against solid tumours *in vivo*, without harming healthy organs. Our transmorphic particle system provides a promising modality for safe and effective gene delivery, and cancer immunotherapies through cross‐species complementation of two commonly used viruses.

## Graphical abstract

This study describes the characterization of a new phage‐derived gene delivery particle capable of systemic targeting (TPA), demonstrating its superiority over existing phage systemic viral vectors (AAVP), and its application in targeted cytokine gene therapy of cancer.

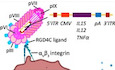

## Introduction

Immunotherapy has the potential to create an enormous impact in cancer treatment if it is able to overcome existing limitations associated with delivery methods and specificity. Because cytokines are pleiotropic molecules produced by immune cells predominantly in the systemic circulation, achieving specific control of their synthesis and release has been an insurmountable challenge (Riley *et al*, 2019). To address this unmet need, we designed and characterised transmorphic particles that systemically target the delivery of recombinant adeno‐associated virus (rAAV) DNA‐bearing cytokine genes using the capsid of a tumour‐targeted prokaryotic viral capsid.

Recruitment of native immunity to target and destroy tumour cells is an effective treatment approach due to the complex array of cellular mechanisms that induce cytotoxicity directly to the pathology (Conforti *et al*, 2018). Since the 1980s, systemically delivered anti‐tumour cytokines and immunoglobulins have been employed in cancer treatment. Today, the five main tactical approaches are the use of cytokines, tumour vaccines, antibodies, immune checkpoint inhibitors and Chimeric Antigen Receptor T‐cells (CAR‐T; Cao *et al*, 2020; Kennedy & Salama, 2020). The use of cytokines is most robust, as much of their signalling pathways are known; however, a fundamental problem is control over when and where they are present to exert the biological effects (Berraondo *et al*, 2018, 2019). The reactivity of cytokines across the diverse cellular and tissue targets is a double‐edged sword. Overactivation of the immune system, that is, a “cytokine storm,” is potentially fatal on the host, and remains a primary concern in clinical translation of immunological agents. Delivering cytokines using gene delivery vectors is a solution, but the inherent biology of existing vectors also needs to be addressed if gene delivery limitations are to be overcome (Riley *et al*, 2019).

Gene delivery is a core technology with potentially broad and impactful applications in immunotherapy. The delivery of cytokine genes at the pathology is a lucrative approach that may effectively target malignant cells whilst sparing normal tissues from the non‐selective effect of cytokines. Even though mammalian viruses are efficient at gene transfer, a key problem is their inherent tropism and immunogenicity, resulting in off‐target transduction, which is of significant concern in the context of cytokine gene delivery (Santiago‐Ortiz & Schaffer, 2016). As a result, existing viral vectors do not allow for effective repeated administrations, as well as require localised delivery to avoid non‐specific transduction (Riviere *et al*, 2006). Significant consideration must also be taken on using mammalian viruses for immunotherapy, as the immunogenic effect of cytokines can be compounded by the immune response to the vector itself, leading to further concerns on safety.

Since the idea of gene transfer was launched in the 1970s, eukaryotic viral vectors have made their impact in gene therapy and *ex vivo* gene transfer; however, their approved applications remain narrow in immunotherapy. In contrast, bacteriophages (phages) are another species of viruses which are capable of gene delivery but have not been extensively investigated. Historically, phages have been used for drug discovery and as an antibiotic due to their complete lack of tropism and pathogenicity to human, and their low immunogenicity. These inherent qualities make them a prime candidate for cytokine gene delivery, where high specificity to the target tissue is required and activation of innate immunity should be avoided. We have attempted to address the parallel challenges in targeting both cytokine and gene delivery by developing a novel phage‐guided system for the delivery of rAAV DNA encoding cytokine genes for cancer immunotherapy. As a result, we characterised a novel system for highly efficient production of transmorphic Phage/Adeno‐associated viral particles (TPA).

Mammalian viruses deliver genes by the use of complex infective mechanisms that coevolved with their mammalian hosts (Waehler *et al*, 2007). Furthermore, they are able to drive stable and long‐term gene expression by the use of mechanisms such as chromosomal integration or protective structures that prevent detection and degradation by the host. These evolutionary mechanisms also give rise to unwanted side effects from immunity or delivery to healthy tissues and cells. Currently commercialised gene therapy vectors, such as Luxturna or Zolgensma, are approved by the Food and Drug Administration (FDA) only for rare diseases with a clearly defined spatial and temporal target (Foust *et al*, 2010; Russell *et al*, 2017). This is because despite being efficient at gene transfer, viral vectors possess native tropism to a wide range of mammalian tissues, similar to how cytokines are reactive towards multiple tissue targets. Indeed, attempts have been made to reduce or ablate the native tropism of mammalian viral vectors in hope that vectors can be delivered systemically, but significant success permitting targeted delivery has yet to be achieved (Anderson *et al*, 2000; Grimm *et al*, 2003; Zincarelli *et al*, 2008). Furthermore, the production and use of mammalian viral vectors come at a great economic cost, with approved therapies costing over 850,000 to over 1.6 million US dollars per treatment (Darrow, 2019; Dean *et al*, 2021). Thus, overcoming challenges that mammalian viruses have in their biology is vital for furthering meaningful progress in the field.

On another side of virology, prokaryotic viruses such as bacteriophages (phages) are routinely used for the very reasons that limit the clinical application of mammalian viruses. Phages are abundant, simplistic viruses that have no native tropism for mammalian tissues, are not pathogenic, and are economical and efficient to manipulate and produce at GMP standards (Regulski *et al*, 2021). The use of phages as an antibiotic was widely accepted and used in the pre‐antibiotic era, but in modern laboratory research, they play an important role in drug discovery *in vitro* and *in vivo* by their ability to tolerate large mutations on their coat proteins with very high binding specificity (Pasqualini & Ruoslahti, 1996; Arap *et al*, 2002; Kutateladze & Adamia, 2010; Bradbury *et al*, 2011). As a result, the use of phages in mammalian gene delivery has been explored through the insertion of a mammalian or viral transgene cassettes in its genome, and a receptor‐specific mutation on its coat protein genes (Larocca *et al*, 1998, 1999, 2001, 2002; Burg *et al*, 2002). An attractive property of the phage in mammalian gene delivery is the target specificity that can be achieved, while at the same time, avoiding significant mammalian immune responses seen in eukaryotic viral vectors. As such, premature vector clearance, as well as harmful and potentially fatal side effects, can be avoided.

At present, phage and chimeric phage vectors for mammalian delivery have not yielded sufficient success in eukaryotic gene delivery. The use of phage‐derived vectors to circumvent the limitations of mammalian viruses is an attractive strategy; however, prokaryotic vectors also face challenges of their own. A significant impediment is their lack of mechanisms to evade intracellular degradation and efficiently induce gene expression when compared to mammalian viruses. Several groups have tried to develop phage vectors for human gene therapy with limited success due to weak gene expression derived from conventional transgene cassettes (Larocca *et al*, 1998, 1999, 2001). Hajitou *et al*, 2006, developed a hybrid vector, the adeno‐associated virus/phage (AAVP), which targeted the angiogenic blood vessels of solid tumours and tumour cells by supplying the phage minor coat protein gene (pIII) with a double cyclic CDCRGDCFC (RGD4C) mutation (Hajitou *et al*, 2006). While enabling applications in targeted therapy and molecular imaging, its efficiency of gene transfer *in vitro* remains incomparable to mammalian viruses. Current phage and phage‐derived vectors continue to possess a fundamental flaw; indeed, the presence of part of the bacteriophage genome or often a full phage genomic sequence dictates the final size of the vector particle. Because filamentous phages have a genome‐dependent particle length, an unnecessarily long capsid gives rise to limitations in replication and packaging, cloning capacity and susceptibility to clearance by the reticuloendothelial system. These factors contribute significantly to poor uptake and induction of gene expression observed in bacteriophage vectors. Previous studies on the AAVP have explored a number of strategies to enhance the relatively low transduction efficiency when compared to conventional mammalian viruses (Kia *et al*, 2013; Przystal *et al*, 2013; Yata *et al*, 2014, 2015; Donnelly *et al*, 2015; Tsafa *et al*, 2016, 2020; Campbell *et al*, 2018). Furthermore, alternative studies have explored removing parts of the phage genome, albeit at the cost of packaging efficiency (Chasteen *et al*, 2006). Together, the challenges present in bacteriophage‐guided gene delivery are inherently constrained by its own reproductive biology.

In this study, we developed a novel approach to systemically targeted cytokine gene delivery using transmorphic particles based on a filamentous phage capsid and the DNA of rAAV‐2 carrying transgene expression cassettes flanked by AAV‐2 inverted terminal repeats (ITRs). The rationale behind combining the phenotype and genotype between two viruses of different kingdom classifications is to combine the specificity and less immunogenic properties of the phage capsid with the efficiency of gene expression observed in rAAV vectors. Using a phage capsid to encapsulate rAAV DNA has the potential to eliminate the native tropism and exceed the cloning capacity of rAAV, which are key limitations imposed by the nature of the AAV capsid and its architecture. Most importantly, phage and AAV are both single‐stranded DNA (ssDNA) viruses, meaning their genomes are compatible for both manipulation and packaging. To achieve this, we used the phage origin of replication in a rAAV plasmid containing a transgene expression cassette of interest and employed a helper phage to supply a capsid bearing the RGD4C mutation on its pIII coat proteins for tumour targeting. By carefully modifying growth conditions, we were able to generate high‐yield TPA particles unseen in any previous study, with low helper phage contamination that can be efficiently removed through ultracentrifugation or fast protein liquid chromatography (FPLC). Unlike previously reported vectors, we completely decoupled the phage genome from the final vector particle, resulting in a compact vector containing only the genetic payload desired for delivery to the target cell. In doing so, we provide evidence of a production system that generates high‐yield, high‐efficacy particles, without any phage structural genes present in the final vector and significant enhancement of gene delivery compared to a phage vector containing the whole phage genome (Larocca *et al*, 1999, 2001; Chasteen *et al*, 2006).

While immunotherapy has been extensively evaluated in haematological cancers and melanoma, targeting solid tumours with current approaches has not been successful due to delivery barriers associated with the tumour interstitial pressure, compressed vasculature and dense extracellular matrix (Riley *et al*, 2019). We performed cancer immunotherapy using three different cytokine genes: interleukin 12 (IL12) and newly designed secreted isoforms of tumour necrosis factor‐α (TNFα) and interleukin 15 (IL15), as they have been shown to produce potent, cell‐mediated anti‐tumour effects (Otani *et al*, 1999; Johansson *et al*, 2012; Waldmann *et al*, 2020). TNFα is a cytokine that achieves tumour killing both through direct induction of apoptosis and recruitment of other immune cells to activate cell‐mediated cytotoxicity and disrupt tumour neoangiogenesis (Johansson *et al*, 2012). Moreover, selective systemic gene delivery of TNFα to cancer by tumour‐targeted phage vectors resulted in tumour growth suppression in mice and pet dogs (Paoloni *et al*, 2009; Tandle *et al*, 2009; Yuan *et al*, 2013; Smith *et al*, 2016). IL12 acts as a bridge between innate and adaptive immunity, activating the Th_1_ response, resulting in cell‐mediated cytotoxicity through the induction of TNFα and interferon gamma (IFN‐y; Otani *et al*, 1999). Similarly, IL15 stimulates cell‐mediated immunity through inducing the proliferation of CD8^+^ cytotoxic T cells and NK cells (Waldmann *et al*, 2020).

Immunotherapy is a potentially safe and powerful tool if met with an equally efficient delivery strategy. Achieving localisation of cytokine expression only at the tumour site will resolve historic concern of side effects when using cytokines for cancer treatment, as well as broadening the application of immunotherapy to a wider range of cancers (Riley *et al*, 2019). We postulated that using TPA particles can confer the ability to safely and systemically, that is intravenous, target cytokine expression in tumours through repeated administrations, which is not achievable by a eukaryotic viral vector. By demonstrating the ability to perform highly targeted immunotherapy, we hope to show the impact that TPA particles can make in targeted immunological therapeutics and beyond.

## Results

### Transmorphic particle design and production

**Figure 1:**
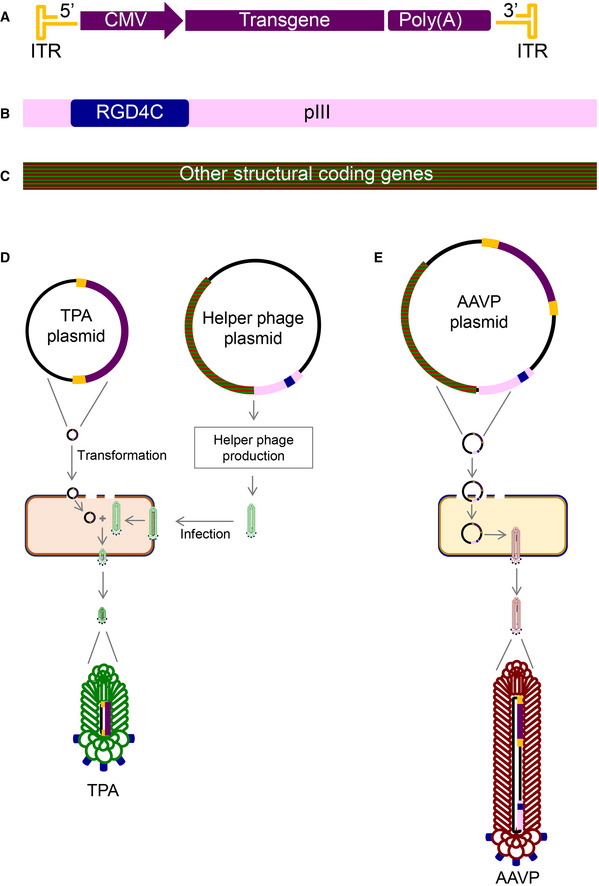
TPA construction A–CProduction of TPA particles requires two key elements: (A) a plasmid containing a mammalian transgene cassette flanked by AAV‐2 ITRs (TPA plasmid) and (B) tumour‐targeted bacteriophage‐derived coat proteins containing the RGD4C peptide insertion mutation on the pIII minor coat protein gene of the M13KO7 filamentous phage, RGD4C.M13KO7, whose genome contains other structural genes required for general other protein subunits for phage assembly (C).DTo encapsidate the AAV DNA cassette using the bacteriophage capsid proteins, the AAV plasmid is transformed into F′ competent *E. coli* hosts and subsequently infected with RGD4C.M13KO7 helper phage. The resulting particle has the external characteristics of a tumour targeted bacteriophage but contains only the AAV DNA transgene cassette encoding a gene of interest.EThe AAVP vector genome contains an inserted transgene cassette from AAV‐2, and an insertion of the RGD4C ligand on the pIII minor coat proteins of the phage display vector fUSE5. The genome of AAVP thus contains both phage structural genes and an AAV transgene cassette.

**Figure EV1:**
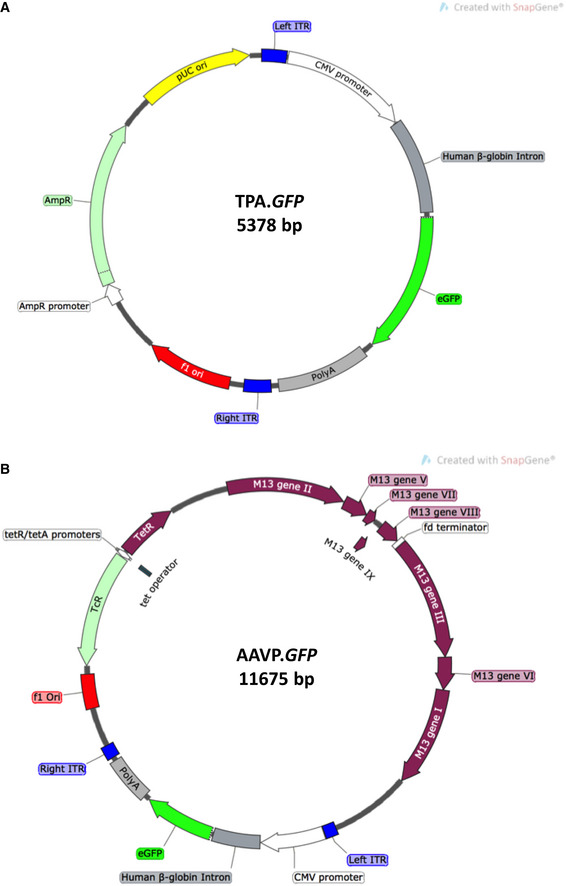
Genetic maps of Transmorphic Phage/AAV, TPA and Adeno‐associated Virus/Phage, AAVP AA schematic diagram of the TPA DNA encoding enhanced eGFP. TPA contains two origins of replication: pUC (high copy‐number, in yellow), which enables double‐stranded DNA replication in prokaryotic hosts, and f1 ori (phage origin of replication, in red), which enables single‐stranded DNA replication and packaging into the phage capsid.BA schematic diagram of the chimeric genome of AAVP encoding eGFP. AAVP contains the full genomic sequence of filamentous bacteriophage, and a transgene cassette from AAV‐2 inserted in to an intergenomic region.

**Figure EV2:**
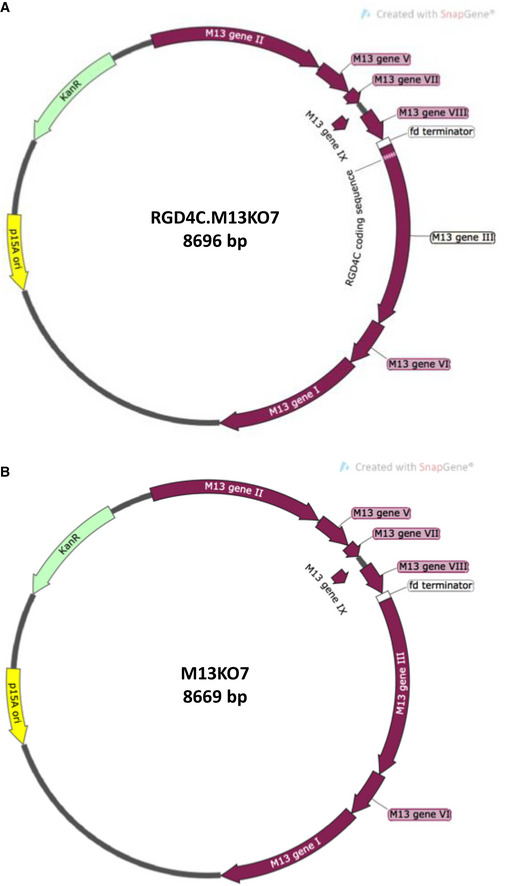
Genetic map of M13KO7 helper phage bearing the RGD4C peptide for tumour targeting AA schematic diagram of the genome of M13KO7 helper phage used for packaging the TPA DNA (shown in Fig EV1) to produce non‐targeted TPA particles. The M13KO7 genome contains a medium copy‐number origin of replication (p15A, in yellow).BA schematic diagram of the genome of RGD4C.M13KO7 helper phage used for packaging the TPA DNA (shown in Fig EV1) to produce the tumour‐targeted RGD4C.TPA particles. The RGD4C coding sequence is inserted in‐frame in to the M13 gene III, which encodes the pIII minor coat proteins.

Transmorphic phage/AAV particles (TPA) were designed and constructed based on the rAAV serotype‐2 DNA, which contains both a pUC high copy‐number origin of replication as well as a phage f1 origin of replication, and a transgene expression cassette flanked by AAV‐2 ITRs (Figs 1A and EV1A). Moreover, production of TPA particles requires a helper phage (Fig 1B–D). The previously reported AAVP contains a full filamentous phage genomic sequence and an expression cassette flanked by AAV‐2 ITRs, thereby significantly increasing its particle size (Figs 1E and EV1B). To generate tumour‐targeted TPA particles, we induced an insertion mutation of the double cyclic RGD4C in the pIII gene of the filamentous M13KO7 helper phage (Figs 1B, and EV2A and B), termed RGD4C.M13KO7 (Fig EV2A), which contains a medium copy‐number p15A origin of replication. In the presence of RGD4C.M13KO7, the rAAV DNA is packaged in bacteria by the tumour‐targeted bacteriophage capsid.

To identify the most efficient production protocol, superinfection and chemically (calcium chloride) competent cell methods were explored at 18 and 40‐h incubation time endpoints with or without the presence of kanamycin (a selection marker for the helper phage). The infective method, done according to the standard reference protocol (Nissim *et al*, 1994), yielded an average of 7 × 10^8^ bacterial transducing units (TU) per μl of TPA in the presence of kanamycin and 3 × 10^8^ TU/μl of TPA without kanamycin. Despite the high number of particles produced, helper phage contamination was significantly higher than the TPA particle yield. In an attempt to identify whether the order of infection will affect particle yield, we generated calcium chloride competent TG1 *Escherichia coli* bearing the RGD4C.M13KO7 genome and transformed the cells with the TPA plasmid. Using identified colonies as a seed for particle production, we observed over four orders of magnitude lower TPA yield after incubation at both 18 and 40‐h timepoints. The presence of kanamycin enabled TPA particle production, but also resulted in low particle yields as well as helper phage contamination.

**Figure 2:**
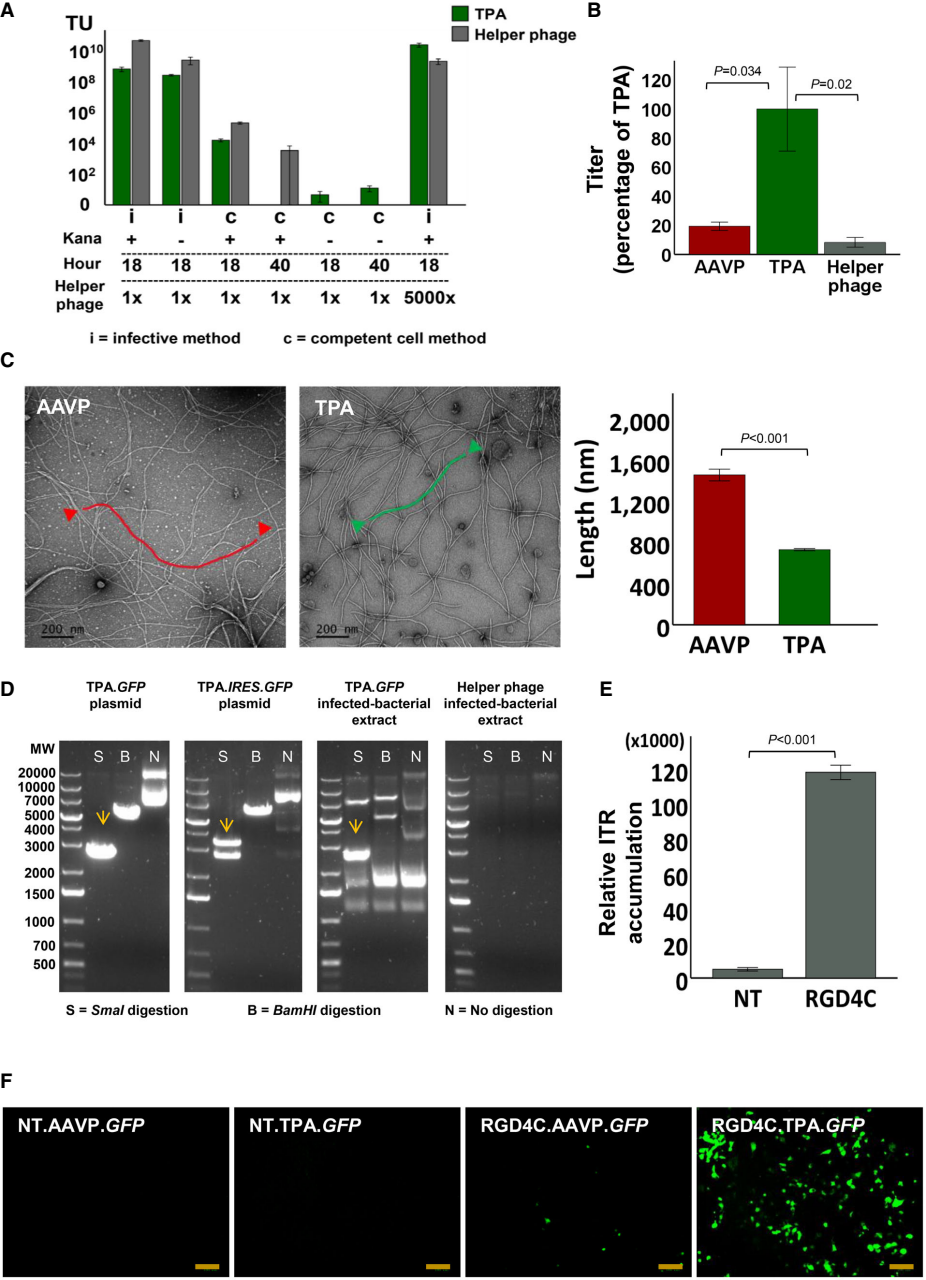
Optimisation of production and physical characterisation of TPA AYield comparison from standardised particle production using either superinfection by RGD4C.M13KO7 of *E. coli* transformed with TPA plasmid (i), or a calcium chloride competent *E. coli* carrying the RGD4C.M13KO7 genome (c), with or without kanamycin for selective expression, and at different incubation time endpoints. Data are expressed as mean ± SEM, and experiments were repeated three times (*n* = 3).BPercentage yield comparison between optimised TPA and AAVP production protocols, normalised to the TPA yield. Data are expressed as mean ± SEM from three independent experiments (*n* = 3), one‐way ANOVA with Tukey HSD test was used.CComparison of mean TPA and AAVP particle length, using a TEM. Scale bar, 200 nm. Independent *t*‐test was used for analysis of data shown in the graph, *n* = 5, technical replicates.D
*SmaI* digestion of plasmid DNA extracted from *E. coli* at 18 h of TPA.*GFP* particle production. The TPA.*IRES*.*GFP* DNA extract was used as positive control to confirm the separation of the two bands and thus complete digestion. Undigested TPA DNA was included and *BamHI* enzyme digestion was used to linearise the TPA plasmid. Extracts from 18 h outgrowth of bacteria carrying either TPA.*GFP* or helper phage were also digested.EQuantification of relative intracellular TPA DNA accumulation in HEK293 cells 2‐h post‐transduction with either targeted (RGD4C) or non‐targeted (NT) TPA, 0.5 × 10^6^ TU/cell, using qPCR and annealing primers specific to the ITR sequence from AAV‐2. Data are expressed as mean ± SEM. The experiment was repeated twice in triplicate and the results shown are representative of one experiment. Independent *t*‐test was used for data analysis.FRepresentative images of GFP‐positive HEK293 cells at day 7 post‐transduction using 1 × 10^6^ TU/cell of RGD4C.TPA.*GFP* or RGD4C.AAVP.*GFP* particles. Non‐targeted (NT) particles were included as control. Data shown are representative of five independent experiments, performed each in triplicates. Scale bar, 100 μm. Source data are available online for this figure.

The standardised infective method, based on a standard phagemid packaging protocol, yielded higher particles compared to the competent cell method (Fig 2A; Larocca *et al*, 2001; Larocca *et al*, 1999; Larocca *et al*, 1998). In addition, a shorter 18‐h incubation period is favourable for higher particle yield; however, extending the incubation period for the infective method is not feasible as the bacterial culture begins to die. These results indicate that RGD4C.M13KO7 and kanamycin are both required for TPA production, and that particle production seems to occur transiently after infection rather than constitutively over time. One problem we had to address, however, was to decrease helper phage contamination. This requires suppression of the competition the helper phage has in packaging its genome in the presence of the TPA DNA. Taking into account that the TPA DNA carries a high copy‐number origin of replication, and RGD4C.M13KO7 carries a medium copy‐number origin, we attempted to generate RGD4C.M13KO7 carrying a low copy‐number origin of replication (pSC101) instead of its medium copy‐number origin (p15A). While positive clones (RGD4C.M13KO7^pSC101^) could be generated and verified by sequencing, no TPA particles could be produced and quantified using this particular mutant. Our findings indicate efficient transmorphic particle production is dependent on sufficient transient expression of RGD4C.M13KO7.

To encourage high‐titre production of transmorphic particles while retaining the original copies of replication, we significantly increased the starting titre of RGD4C.M13KO7 to 1 × 10^12^ TU, while keeping similar incubation conditions for 18 h (Fig 2A and B). The optimised protocol yielded 5 × 10^10^ TU/μl particles, which was _~_100 × times higher than the standard protocol (Fig 2A). To put particle production into context, we compared the yield between TPA and a standard production protocol for AAVP (Fig 1E). When normalised to transmorphic particle yield, AAVP production generated only 20% of the TPA particle yield in our optimised TPA production protocol (Fig 2B). Helper phage contamination in the sample could be further suppressed to 2%, relative to TPA. Moreover, helper phage can be completely removed by ultracentrifugation or fast FPLC; however, this was omitted in our characterisation studies to control for vector preparation used for AAVP production.

### Particle size measurement and determination of ITR stability in TPA particles

Because TPA are packaged from a rAAV DNA, the total genome size should be significantly reduced given the small size of AAV compared to the phage genome. As such, we hypothesised the reduction in genome size would translate into particle length alteration. Using Transmission Electron Microscopy (TEM), we negative‐stained diluted transmorphic particles using uranyl acetate to measure particle length and compared it to the length of AAVP particles containing both rAAV DNA and phage genome (Fig EV1B). The findings support our hypothesis that the reduction of genome size results in a proportional reduction of particle length (Fig 2C). The 5,378 bp TPA genome produced vectors with a mean length of 729 nm, while the 11,675 bp AAVP genome produced vectors with approximately twice the average length at 1,480 nm (Fig 2C). No observable differences in filament diameter between TPA and AAVP were detected across all micrographs imaged.

The stability of ITR sequences flanking the gene of interest serves as a protective hairpin structure that confers the ability of viral genome persistence and concatemer formation, enabling long‐term gene expression (Hajitou *et al*, 2006). To determine whether the ITRs remain intact after vector production from a bacteria culture, the particle DNA extraction from a TPA production overnight culture (infected with M13KO7 or RGD4C.M13KO7) was done at 18‐h post‐inoculation and digested with *SmaI*, a restriction enzyme that specifically digests one site inside the ITR sequences on both 3′ and 5′ ends of the AAV genome. The results showed the two ITR sequences flanking the gene of interest of the TPA genome remain fully intact and thus digested when compared to a *BamHI*‐linearised DNA extract, producing two bands of approximately similar molecular weight (2,681 bp) corresponding both to the transgene cassette and particle backbone (Fig 2D). The two bands were also produced upon *SmaI* digestion of TPA‐infected bacterial extract, but not bacteria infected with helper phage.

After confirming the ITRs remain unaltered in the vector following production in bacteria, we sought to ascertain whether TPA delivers intact ITRs and thus AAV DNA into the target mammalian cells. Hence, we treated human embryonic kidney (HEK293) cells with targeted RGD4C.TPA.*GFP* vector carrying the *green fluorescent protein* (GFP) reporter gene or non‐targeted NT.TPA.*GFP* lacking the RGD4C mutation, as control for targeting, that we produced with a non‐targeted (NT) M13KO7 helper phage. We subsequently subjected the cell‐lysate to qPCR with primers specific to the AAV‐2 ITRs, 2‐h post‐transduction, as previously reported (Aurnhammer *et al*, 2012; Tsafa *et al*, 2016, 2020). RGD4C.TPA.*GFP* was able to successfully deliver AAV‐ITRs to cells when compared to control NT.TPA.*GFP* particles (Fig 2E). These results indicate RGD4C.TPA particles are able to specifically deliver the AAV transgene cassette to the target cells when packaged using the RGD4C.M13KO7 helper phage.

### Evaluation and quantification of gene expression by TPA particles

**Figure EV3:**
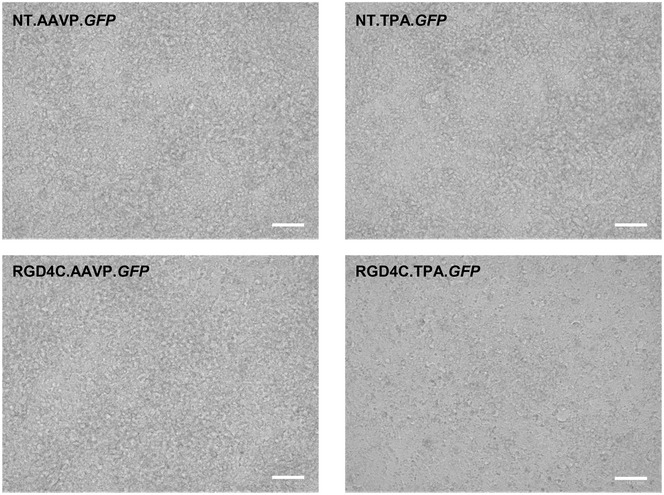
Phase contract images of HEK293 cells treated with TPA.*GFP* and AAVP.*GFP* Images of cells were obtained at day 7 post‐treatment with 1 × 10^6^ TU/cell of either RGD4C.TPA.*GFP* or RGD4C.AAVP.*GFP*. Non‐targeted (NT) vectors were included as controls. Data shown are representative of three independent experiments (*n* = 3). Scale bar, 100 μm. Source data are available online for this figure.

To confirm delivery of the AAV transgene cassette to the cell results in gene expression, we first carried out a qualitative analysis of gene delivery to HEK293 cells by comparing gene expression between RGD4C.TPA.*GFP* and RGD4C.AAVP.*GFP*. AAVP vectors were used as comparison for the effect of particle size imparted by its full phage genomic sequence. Non‐targeted vectors were also included and added to HEK293 cells, as negative controls. At day 7 post‐transduction, microscopic analysis of GFP expression showed extensive GFP production in HEK293 cells transduced by RGD4C.TPA.*GFP*, distinctly higher than that of cells treated with the RGD4C.AAVP.*GFP* (Figs 2F and EV3). Importantly, no GFP expression was detected in cells treated with the non‐targeted NT.TPA.*GFP*, proving gene delivery by targeted transmorphic particles remains selective to integrin‐expressing cells and mediated by the RGD4C ligand. NT.AAVP.*GFP* similarly did not show any GFP‐positive cells. Collectively, these results prove RGD4C.TPA particles deliver a functional AAV transgene cassette to mammalian cells.

**Figure 3:**
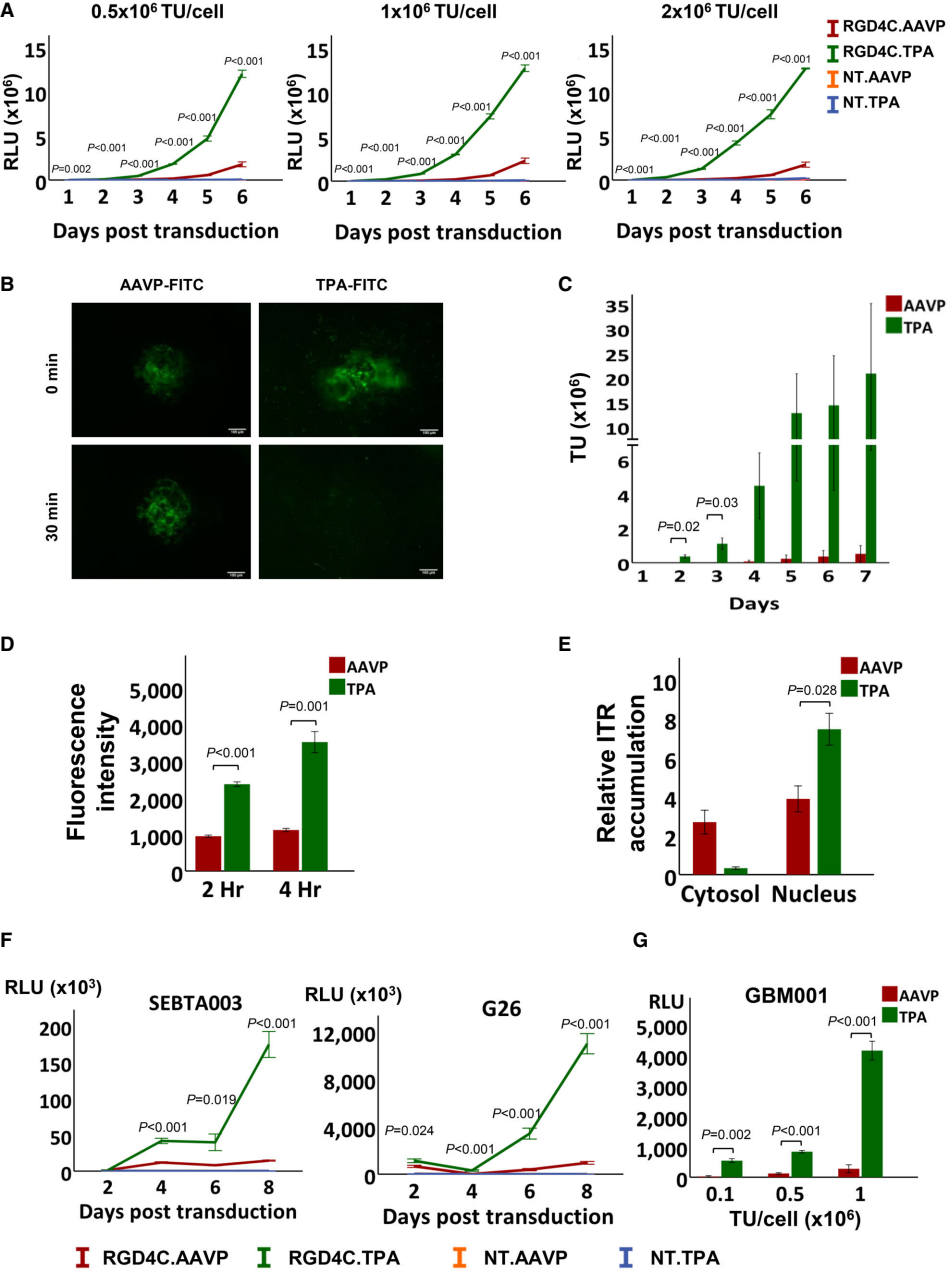
Characterisation of TPA‐mediated gene delivery AQuantification of gene expression in HEK293 cells after transduction with 0.5 × 10^6^, 1 × 10^6^ and 2 × 10^6^ TU/cell of RGD4C.TPA, RGD4C.AAVP or non‐targeted (NT) particles carrying *Lucia*. Luciferase activity was measured at days 1 to 6 post‐transduction and shown as relative light units (RLU). Data shown are representative of three independent experiments (*n* = 3), and are expressed as mean ± SEM. One‐way ANOVA with Tukey's HSD test was used for data analysis.BRepresentative images of FITC‐labelled TPA and AAVP particle diffusion in Matrigel at 5‐ and 30‐min post‐inoculation (*n* = 3). Scale bar, 100 μm.CParticle diffusion using a vertical trans‐well system partitioned by Matrigel. Samples were collected from the wells below the Matrigel at days 1 to 7, *n* = 3, technical replicates. Data are expressed as mean ± SEM, independent *t*‐test was used for data analysis.DComparison of AAVP and TPA internalisation efficiency in HEK293 cells as evaluated by staining of the phage capsid proteins, then quantified by FACS at 2‐ and 4‐h post‐transduction, 0.1 × 10^6^ TU/cell, and normalised to particle length. Data are expressed as mean ± SEM of *n* = 3 independent experiments, independent *t*‐test was used for data analysis.EQuantification of ITRs in the cytosol and nucleus of HEK293 cells 24‐h post‐transduction with 0.5 × 10^6^ TU/cell of RGD4C.TPA and RGD4C.AAVP, using qPCR with primers specific to AAV‐2 ITRs. Data are representative of *n* = 3 independent experiments and expressed as mean ± SEM, independent *t*‐test was used for data analysis.FQuantification of *Lucia* reporter gene expression following transduction of human primary SEBTA003 glioblastoma and G26 glioblastoma‐derived neural stem cells with 1 × 10^6^ TU/cell. Data are expressed as mean ± SEM of *n* = 3 independent experiments. One‐way ANOVA with Tukey's HSD test was used for data analysis.G3D primary GBM001 tumour spheres subjected to transduction by increasing doses of TPA and AAVP encoding *Lucia*. Experiments were repeated three times and performed in triplicates. Data are shown as mean ± SEM and are representative of one experiment. Independent *t*‐test was used for data analysis. Source data are available online for this figure.

Next, we performed a quantitative analysis of gene delivery by using NT and RGD4C targeted TPA particles carrying a reporter gene encoding a secreted Gaussia luciferase (*Lucia*; Wurdinger *et al*, 2008). Gene expression was quantified by analysis of luciferase activity in the growth media (Fig 3A). We tested varying doses of particles and evaluated gene expression over 6 days. The data revealed gene expression from the RGD4C.TPA.*Lucia* was detected as early as 2 days following treatment and increased gradually over time, with all doses tested. In contrast, gene expression by RGD4C.AAVP was only detectable at day 4 post‐transduction and was consistently and significantly lower than that of RGD4C.TPA across all timepoints and doses tested (Fig 3A). Both control (NT.TPA.*Lucia* and NT.AAVP.*Lucia*) vectors showed minimal luminescence attributed to the background. These findings indicate the enhancement of gene expression observed may be a consequence of earlier induction as well as a greater number of successfully transduced cells, or perhaps a combination of these two non‐mutually exclusive events.

### Investigation of mechanisms of transgene expression

To gain an insight into the molecular mechanisms of RGD4C.TPA‐mediated transgene expression and its superiority to RGD4C.AAVP, we investigated the extracellular and intracellular fate of the particles following treatment of mammalian cells and compared side‐by‐side with the RGD4C.AAVP. Cellular binding is the first key stage in viral transduction. However, prior to reaching the cell membrane, vectors must pass through the extracellular matrix (ECM) before becoming bioavailable to their receptors on the cell surface. To observe whether transmorphic particles are able to diffuse through the ECM, we labelled the coat proteins of RGD4C.TPA and RGD4C.AAVP with fluorescein isothiocyanate (FITC) and seeded each vector onto 5 mg/ml Matrigel Matrix, used as an *in vitro* model of ECM. At 5‐min post‐seeding, fluorescence microscopy revealed that transmorphic particles had already begun to diffuse rapidly through the Matrigel in multiple directions while the AAVP sample remained localised around the site of seeding (Fig 3B). After 30‐min, transmorphic particles had diffused completely and pervasively throughout the Matrigel, showing no specific signal localisation in the field of view, whereas the AAVP remained localised around the site of inoculation (Fig 3B). These findings suggest that transmorphic particles are able to move through the ECM to the target site, possibly due to their smaller size.

To ascertain whether this effect of dissemination through a Matrigel Matrix translates into movement across different compartments, we repeated the experiment in a Matrigel Matrix transwell system. We inoculated the top compartment with RGD4C.TPA or RGD4C.AAVP and quantified the transducing units of vectors in the bottom compartment every 24‐h for 7 days. The results showed that TPA particles were able to migrate through the Matrix from the top to bottom compartments as early as 24‐h post‐inoculation, whereas the AAVP was only able to migrate after 4‐day post‐inoculation (Fig 3C). Furthermore, the number of transmorphic particles found in the bottom compartment was 60‐, 55‐, 40‐ and 40‐fold higher than AAVP at days 4, 5, 6 and 7 respectively. These data indicate a dramatically increased particle diffusion *in vitro* as an effect of reduced physical size.

We next sought to establish whether the reduced particle length of our transmorphic particles could also translate into higher cell entry. We performed an internalisation assay by transducing HEK293 cells with RGD4C.TPA.*GFP* or RGD4C.AAVP.*GFP* and arrested the transduction process at 2‐ and 4‐h post‐transduction. Upon fixing and staining the phage coat proteins present in the intracellular compartments, we detected higher internalisation efficiency by RGD4C.TPA when compared to RGD4C.AAVP (Fig 3D). Indeed, transmorphic particles displayed higher overall uptake in HEK293 cells relative to AAVP, with a 2.5‐fold increase in fluorescence intensity at 2 h, and over 3.5‐fold higher fluorescence intensity at 4‐h post‐transduction. These results indicate that transmorphic particles are taken up more quickly and to a greater extent when compared to AAVP.

Finally, we carried out quantification of the ITRs in the nucleus since a hallmark of successful TPA‐mediated AAV transgene expression is sufficient nuclear ITR accumulation to enable efficient and long‐term gene expression. To prove transmorphic particles are able to efficiently deliver their DNA to the host nuclei, we quantified ITRs delivery in to the cytoplasmic and nuclei compartments of HEK293 cells after transduction with RGD4C.TPA and RGD4C.AAVP 24‐h post‐transduction using qPCR (Fig 3E). Our results showed that transmorphic particles delivered significantly greater copies of their DNA into the host nuclei when compared to AAVP, which remained significant in the cytoplasm with no significant changes of distribution at 24‐h post‐transduction. These results indicate the observed differences in gene expression may also be attributed to the efficiency of particle DNA delivery to the nucleus. Altogether, these data suggest RGD4C.TPA may bestow an advantage in gene expression by means of an acquired diffusion through the ECM, better entry into mammalian cells, enhanced delivery of TPA DNA into the nucleus, or perhaps even a combination of these non‐mutually exclusive mechanisms.

### Quantification of gene expression in human primary glioblastoma cells, primary tumour stem cells and primary 3‐dimensional glioblastoma spheroids

To determine whether successful TPA‐induced gene transfer can also be achieved in tumour cells for therapeutic applications, we conducted transductions of human glioblastoma, also known as glioblastoma multiforme (GBM) or grade IV astrocytoma, since this particularly brain tumour remains a challenge to treat in patients. GBM is the most common and fatal of primary brain tumours in adults, with a 14.6‐month median survival time and a 2% 5‐year survival rate (Kwiatkowska *et al*, 2013). Glioblastoma multiforme is a heterogeneous tumour with low survival that has been, at least partly, caused by glioma stem cells. These therapy‐resistant GBM stem cell sub‐populations are able to resist standard treatment and sustain relapse (Kwiatkowska *et al*, 2013; Przystal *et al*, 2019). We conducted cell transduction in a diverse panel of human primary GBM and GBM stem cells using RGD4C.TPA in comparison with RGD4C.AAVP (Fig 3F). We treated adherent human SEBTA003 glioblastoma cells and G26 glioblastoma stem cells. Both are primary cell samples obtained from patients with GBM. Using particles carrying *Lucia*, we observed successful delivery of gene expression by the RGD4C.TPA with luminescence starting at day 4 and peaking at day 8 post‐transduction by transmorphic particles. In contrast, no gene delivery was detected in these primary cells by the RGD4C.AAVP vector.

Because the tumour microenvironment is complex and difficult to penetrate by therapeutic agents and delivery systems, we similarly performed transmorphic particle transduction of patient‐derived GBM001 glioblastoma cells, which are able to form three‐dimensional (3D) spheres that are heterogenous and rich in glioblastoma stem cells (Przystal *et al*, 2019), mimicking human GBM *in vivo* (Fig 3G). The 3D tumour spheroids are considered valid models with features of solid tumours and were used in this study to evaluate the efficacy of gene delivery by RGD4C.TPA and its comparison to RGD4C.AAVP. The results showed that RGD4C.TPA.*Lucia* particles were able to efficiently transduce GBM001 spheres in a dose‐dependent manner, while RGD4C.AAVP vectors were unsuccessful at transduction (Fig 3G). These results can be related, at least partly, to the diffusing ability of TPA. Together, our findings in primary tumour cells, tumour stem cells and primary GBM spheroid models indicate strong *in vitro* evidence for gene delivery and therapeutic potential of transmorphic particles in cancer over the existing AAVP particles.

### *In vivo* tumour immunotherapy using transmorphic particles bearing a *TNFα* transgene

**Figure 4:**
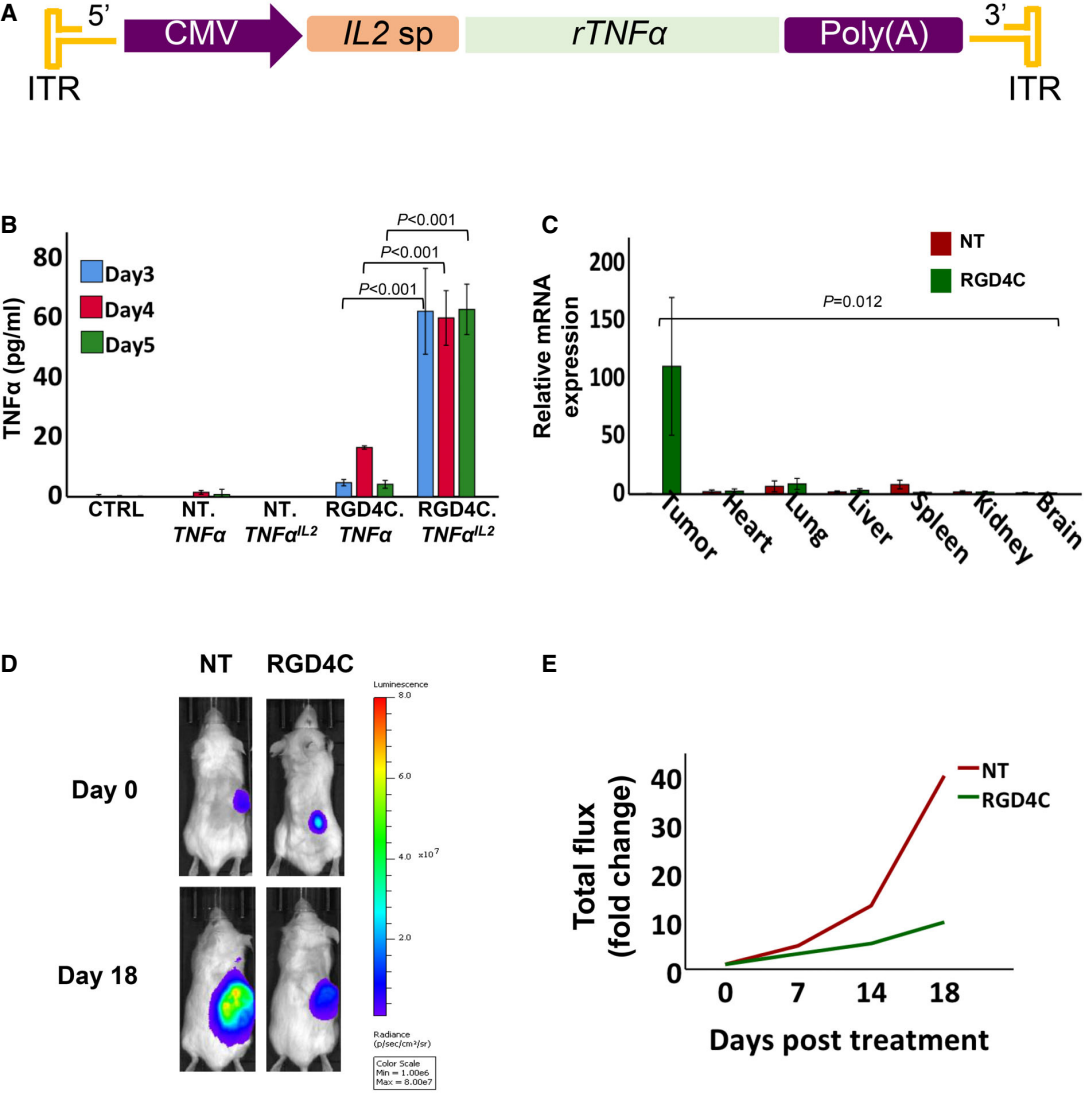
Targeted TNFα^IL2^ cytokine treatment of solid tumours ATPA particle DNA carrying the sequence encoding a secreted human soluble TNFα, whose secretion is controlled by the human IL2 signal peptide (sp).BELISA quantification of TNFα production in media collected on days 3 to 5 post‐transduction of human primary GBM001 tumour spheres at 1 × 10^6^ TU/cell of targeted (RGD4C) or non‐targeted (NT) TPA particles carrying native *TNF*α or recombinant *TNF*α^*IL2*^ sequences. Untreated cells were included as control (CTRL). Data are representative of one experiment and expressed as mean ± SEM. Experiments were repeated twice in triplicates, one‐way ANOVA with Tukey's HSD test was used for data analysis.CBiodistribution of gene delivery upon intravenous administration of 5 × 10^10^ TU of NT or RGD4C.TPA.*TNFα* ^*IL2*^ to NOD/SCID mice bearing subcutaneous human GBM001. Tumours and healthy organs were harvested at day 18 post‐transduction and analysed using RT‐qPCR to quantify the *TNFα* ^*IL2*^ transcripts. Data shown are expressed as mean ± SEM, from *n* = 3 mice. Two‐way ANOVA was used for data analysis.DBLI of *Luc* showing representative mice with subcutaneous GBM001 labelled with *Luc* gene, at day 18 post‐targeted RGD4C.TPA.*TNFα^IL2^* treatment.EAnalysis of tumour growth in representative mice, from D, using the tumour bioluminescence data (total flux) and expressed as fold changes of tumour luminescence flux at different time points compared to day 0. Source data are available online for this figure.

To corroborate our *in vitro* characterisation studies in preclinical disease models, we constructed transmorphic particles bearing a transgene encoding TNFα, a known anti‐tumour cytokine, for the treatment of NOD/SCID immunodeficient mice bearing subcutaneous GBM001 tumours. While patient‐derived xenograft (PDX) models more closely replicate human disease, their requirement for using immunocompromised animals creates challenges when translating results into humans with intact immune systems (Day *et al*, 2015). To demonstrate proof‐of‐efficacy of TPA‐mediated cytokine therapy in a PDX model, we chose to deliver a *TNFα* transgene, since this cytokine can induce direct tumour cell death. In humans, TNFα is released via proteolytic cleavage from transmembrane TNFα by the metalloproteinase, TNF‐alpha‐converting enzyme (TACE). More specifically, TNFα mainly activates the TNFR1 receptor, which is constitutively expressed by almost any cell type. It has limited signalling capacities on the TNFR2 receptor, however, which is expressed in immune cells including myeloid cells, regulatory T‐cells, glial cells and some endothelial cell types (Medler & Wajant, 2019). Interestingly, TNFα promotes significant cytotoxicity through activating its receptors and induces apoptosis of many tumour cell types (Wajant *et al*, 2003; Josephs *et al*, 2018). The effectiveness of the anti‐tumour activity of TNFα can depend on the levels of TNFα released in the ECM and thus on the tumour levels of TACE expression. To ensure constitutive TNFα secretion, independent of TACE, and enhance its activity, we designed a new secreted TNFα version as a hybrid between human TNFα and the potent signal peptide of human interleukin 2 (IL2), known for its high secretion. To achieve this, the transmembrane domain of TNFα was removed, and the resulting recombinant TNFα (rTNFα) was fused with the IL2 signal peptide to produce a hybrid *TNFα* ^*IL2*^. Thus, we constructed transmorphic particles carrying a sequence for the secreted TNFα^IL2^ (TPA.*TNFα* ^*IL2*^) (Fig 4A). We also constructed particles carrying the native TNFα DNA sequence (TPA.*TNFα*) for a side‐by‐side comparison to select for the most suitable TNFα version to test in further investigations.

We first investigated whether TPA particles encoding native or secreted versions of TNF*α* could potentially be effective to deliver TNF*α* to tumours (Fig 4B). We transduced 3D cultures of the human primary glioblastoma GBM001 spheres with targeted RGD4C.TPA.*TNFα* or RGD4C.TPA.*TNFα* ^*IL2*^ and measured the concentration of TNF*α* in the media using ELISA. We found the expression and release of TNF*α* were only detectable and significant with the RGD4C‐targeted particle encoding the secreted *TNFα* ^*IL2*^ isoform across all timepoints on days 3, 4 and 5, compared to particles encoding the native *TNFα* (Fig 4B). Moreover, no TNFα^IL2^ was detected in media of cells treated with the non‐targeted particle (lacking RGD4C) whose data were similar to untreated cells. This is important because TNFα possesses strong anti‐tumour activity but induces systemic toxicity and has to be targeted to tumours. Consequently, we selected RGD4C.TPA.*TNFα* ^*IL2*^ for subsequent *in vivo* studies.

To translate our findings to *in vivo* studies, we first performed a biodistribution investigation of TNFα expression in immunodeficient mice with established human subcutaneous primary GBM, upon intravenous delivery of RGD4C.TPA.*TNFα* ^*IL2*^. For this, we injected tumour‐bearing mice with 5 × 10^10^ TU/mouse, as the dose we have used previously for phage‐based vectors, then applied RT‐qPCR to identify the expression of TNFα mRNA transcripts in the tumour and key internal organs (Fig 4C). These biodistribution experiments were initially performed before therapy studies to ensure that TNFα expression is selective to tumours established in mice after intravenous administration of the RGD4C.TPA.*TNFα* ^*IL2*^ particles without any expression in healthy tissues, that can lead to off‐target effects. We detected significant expression of the *TNFα* mRNA transcript in the tumours, but not in other tested tissues upon three intravenous doses on days 0, 2 and 4. The results show that RGD4C.TPA.*TNFα* ^*IL2*^ efficiently and systemically targets the tumours while sparing other key internal organs. Non‐targeted particles did not show significant expression in the tumours or any of the organs studied.

Knowing our transmorphic particles can specifically transduce tumours *in vivo*, we evaluated the therapeutic efficacy of these particles by repeated administrations twice per week over 2 weeks. To mimic the clinical situation of cancer patient treatment, intravenous administrations of RGD4C.TPA.*TNFα* ^*IL2*^ were only initiated following detection of tumours in mice. Moreover, to monitor tumour response to treatment by imaging, we implanted mice subcutaneously with GBM001 cells stably labelled with the Firefly *Luciferase (Luc)* gene, and mice were serially imaged using bioluminescent imaging (BLI) of *Luc* expression in tumours (Fig 4D). Treatment was initiated when tumours were sizeably evident and confirmed by imaging. Analysis of the imaging data showed that the tumours grew very large between day 0 (treatment initiation), to end of treatment on day 18 in the control groups of mice injected with non‐targeted NT.TPA.*TNFα* ^*IL2*^ particle. In contrast, mice administered with the targeted RGD4C.TPA.*TNFα* ^*IL2*^ particle had their tumour load dramatically reduced, on day 18, relative to control mice receiving non‐targeted particle (Fig 4D). To support these data, serial evaluation of the tumour bioluminescent signals, between days 0 and 18, showed steady increase in the tumour viability in the groups of mice treated with the non‐targeted NT.TPA.*TNFα* ^*IL2*^ (Fig 4E). Interestingly, in the treatment group where targeted particles were administered, mice had reduced tumour viability from day 4 post‐treatment and across all time points compared to the control group, where the non‐targeted vector was used. Taken together, these findings demonstrate the new secreted hybrid TNFα^IL2^ can be used safely and efficiently to inhibit the growth of GBM in combination with the tumour‐targeted RGD4C.TPA particles.

### Construction and function analysis of transmorphic particles bearing interleukin 15 (IL15) transgene

**Figure 5:**
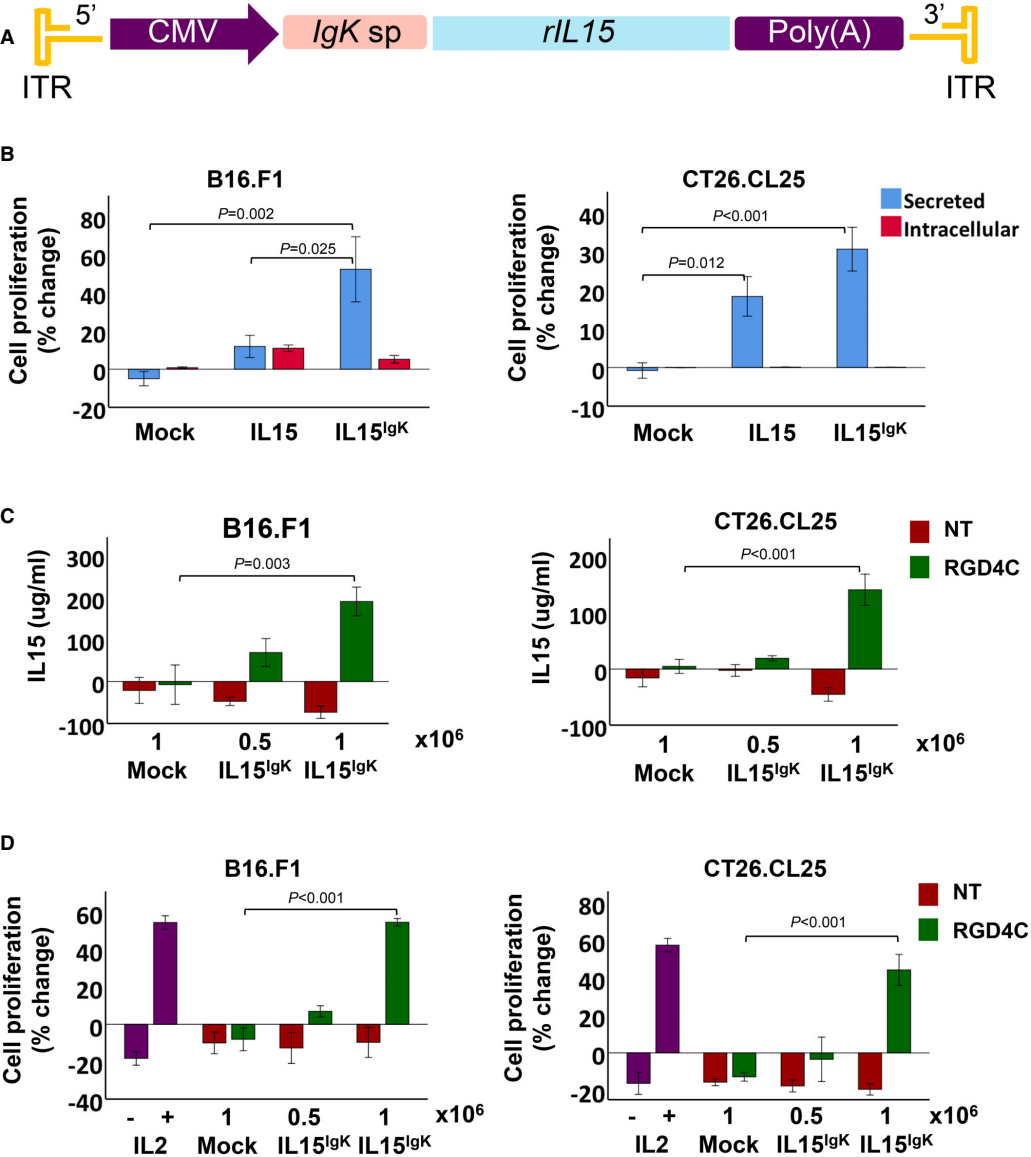
Design, construction and *in vitro* investigation of TPA carrying *IL15* ATPA DNA carrying a sequence encoding a mouse IL15^IgK^ whose secretion is under the mouse IgK sp.BInvestigation of the TPA.*IL15* ^*IgK*^ in comparison to the native TPA.*IL15* in B16.F1 and CT26.CL25 tumour cells. The TPA DNA constructs were transfected into tumour cells, then the bioactivity of secreted IL15 was assessed by evaluating the effects of media from transfected cells on the proliferation of mouse CTLL‐2. TPA DNA without IL15 (mock) was included as control. Data are representative of one experiment and expressed as mean ± SEM. Experiments were repeated twice and performed in triplicates, and one‐way ANOVA with Tukey's HSD test was used for data analysis.CTargeted (RGD4C) and non‐targeted (NT) TPA particles carrying *IL15* ^*IgK*^ were produced and used to transduce B16.F1 or CT26.CL25 cells at 0.5 × 10^6^ or 1 × 10^6^ TU/cell. Then secreted IL15 was quantified by ELISA in tumour cell media collected on day 5 post‐transduction. RGD4C.TPA without *IL15* ^*IgK*^ (mock) was also included as control. Experiments were repeated twice and shown are the results from a representative experiment. Data are expressed as mean ± SEM, one‐way ANOVA with Tukey's HSD test was used for data analysis.DEvaluation of CTLL‐2 proliferation in the presence of media collected from B16.F1 or CT26.CL25 tumour cells after transduction with TPA particles carrying *IL15* ^*IgK*^. Cells treated with a recombinant IL2 cytokine were included as a positive control. Data are representative of one experiment, expressed as mean ± SEM and shown as change in CTLL‐2 number relative to treatment initiation day. Experiments were repeated twice and in triplicates, one‐way ANOVA with Tukey's HSD test was used to analyse the data. Source data are available online for this figure.

While our therapeutic study on particles delivering TNFα yielded positive results, it is vital that immunocompetent preclinical models are also explored due to the complex nature of the intact immune system. To rule out the possibility the observed anti‐tumour effects were cytokine‐specific, we performed immunotherapy with IL15 in wild‐type mice bearing syngeneic solid tumour models. IL15 has been a primary cytokine of interest in immunotherapy due to its ability to activate the main anti‐tumour cell effectors, but does not, in contrast to IL2, stimulate immune‐suppressing regulatory T cells (T_reg_). Unfortunately, the recombinant human IL15 protein used in clinical trials, to treat cancer patients, undergoes a rapid renal clearance and has a short plasma half‐life which diminishes its anti‐tumour effects (Chertova *et al*, 2013). Furthermore, the systemic administration of IL15 carries the risk of potential toxic side effects, including the induction of autoimmunity (Tay *et al*, 2020). Thus, we postulated that targeted gene delivery using transmorphic particles may potentially improve the clinical application of IL15 in cancer immunotherapy. To increase the therapeutic potential of IL15 delivery by TPA particles, we redesigned the IL15 transgene to encode a novel secreted isoform of IL15 as a hybrid with a mouse immunoglobulin IgK signal peptide, named IL15^IgK^ (Fig 5A). Thus, our additional primary objective was to redesign the IL15 in order to overcome the limited secretion of its native version. Therefore, the native signal peptide of IL15 was removed and replaced with the IgK signal peptide to produce a new IL15^IgK^ isoform.

We first used transfection with TPA plasmid, to investigate the potential of the IL15^IgK^ isoform to stimulate T cell proliferation, before packaging into transmorphic particles. Upon DNA transfection of murine B16.F1 melanoma and CT26.CL25 colorectal carcinoma cells, we assessed the activity of secreted IL15 by incubating the CTLL‐2 murine cytotoxic T lymphocytes with media from transfected B16.F1 and CT26.CL25 cells (Fig 5B). We found a significant increase in the proliferation of CTLL‐2 cells treated with media from B16.F1 and CT26.CL25 cells transfected by TPA.*IL15* ^*IgK*^ DNA transgene cassette compared to media from cells transfected with TPA.*IL15*. The treatment group cultured with media from the control (untransfected) cells did not show any changes in T cell proliferation.

Next, we packaged the TPA.*IL15* ^*IgK*^ DNA construct into transmorphic particles using RGD4C‐ and non‐targeted helper phage, and transduced B16.F1 and CT26.CL25 cells using 0.5 × 10^6^ and 1 × 10^6^ TU/cell (Fig 5C). The data revealed that RGD4C.TPA.*IL15* ^*IgK*^ induced a dose‐dependent increase in IL15 production and secretion determined by ELISA with significance at 1 × 10^6^ TU/cell. Non‐targeted NT.TPA.*IL15* ^*IgK*^ and mock RGD4C.TPA, lacking *IL15* ^*IgK*^, did not induce IL15 production. Next, to demonstrate that IL15 secretion by transmorphic particles is bioactive, we used media from transduced cells to maintain the culture of the CTLL‐2 cells (Fig 5D). The data showed the significant secretion of IL15 translated into a significant increase in CTLL‐2 cell proliferation by both B16.F1 and CT26.CL25 media and was comparable to the induction of CTLL‐2 cell proliferation by recombinant IL2 used in this experiment as a positive control. No CTLL‐2 proliferation was observed upon treatment with transduction media from non‐targeted NT.TPA or mock RGD4C.TPA.

### Tumour immunotherapy using transmorphic particles bearing IL15 transgene

**Figure 6:**
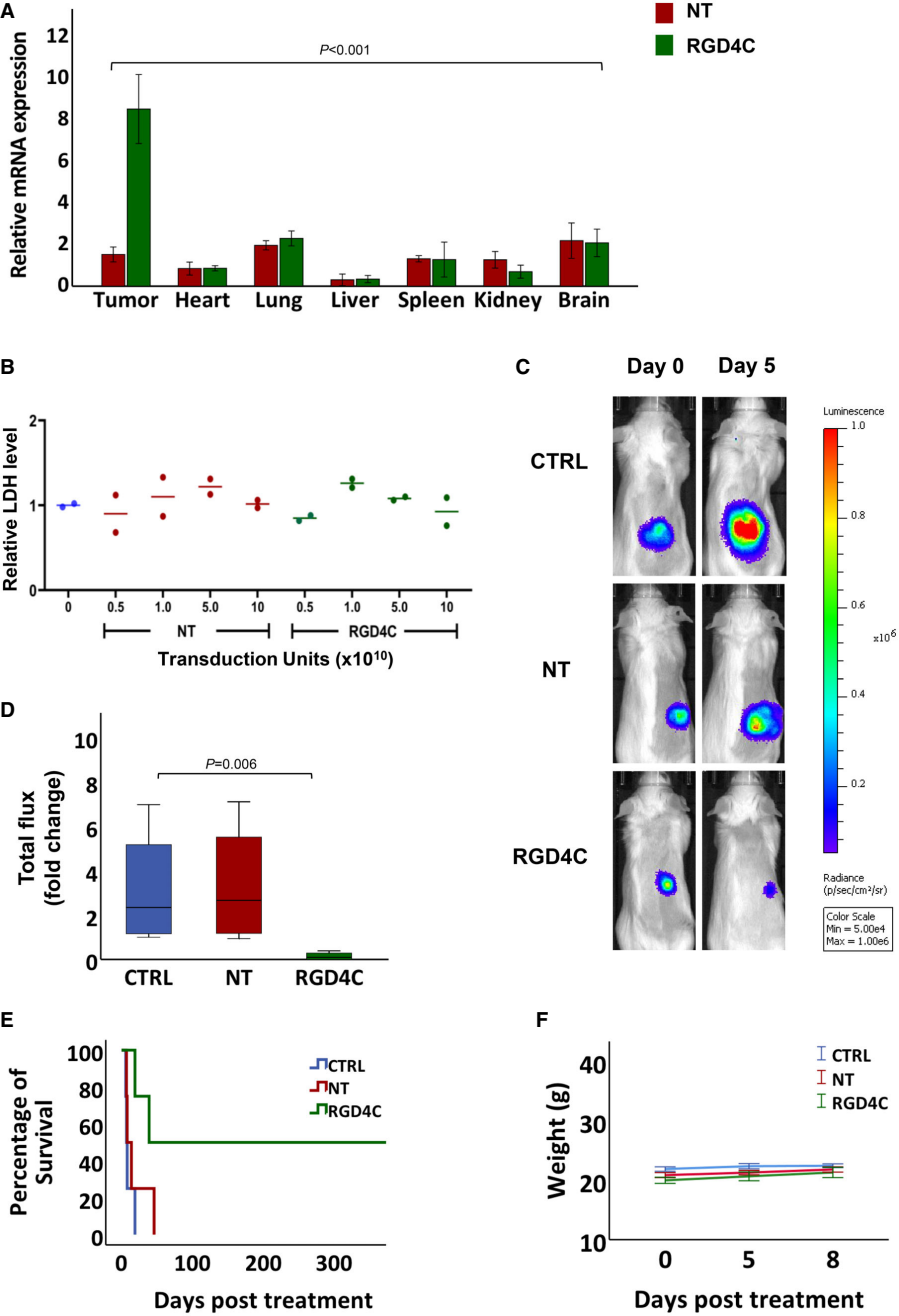
Biodistribution and targeted systemic cancer immunotherapy with RGD4C.TPA.*IL15* ^*IgK*^ ATumour‐bearing BALB/c mice with established subcutaneous CT26.CL25 tumours were intravenously injected with a single dose, 5 × 10^10^ TU/mouse, of targeted (RGD4C) or non‐targeted (NT) TPA.*IL15* ^*IgK*^. Gene delivery was evaluated by quantifying the *IL15* ^*IgK*^ mRNA expression in tumours and healthy tissues at day 5 post‐TPA administration. Data are representative of one experiment, *n* = 3, and expressed as mean ± SEM. Two‐way ANOVA test was used for data analysis.BSafety of dose escalation regimens assessed at day 5 following TPA delivery, by evaluating the serum levels of LDH from *n* = 2 mice/TPA dose of targeted (RGD4C) or non‐targeted (NT) TPA.*IL15* ^*IgK*^. Serums from untreated mice were also analysed.CRepresentative tumour‐bearing mice showing bioluminescence imaging of luciferase on days 0 and 5 post‐administration with 5 × 10^10^ TU of RGD4C.TPA.*IL15* ^*IgK*^ (RGD4C) or non‐targeted (NT) particles. The CT26.CL25 tumour cells stably express a luciferase *Luc* reporter gene. Untreated mice were also included as controls (CTRL).DChanges in tumour viability in mice (*n* = 4) from day 0 to day 5 post‐TPA administration. Tumour viability was measured by bioluminescence activity. The central band represents the median of the data. The boxes upper and lower lines represent quartile 3 and quartile 1 of the data respectively. The whiskers represent the maximum and minimum outliers of the data. Nonparametric Kruskal–Wallis test was used for data analysis.EKaplan–Meier curves showing survival benefit for tumour‐bearing mice (*n* = 4) from all experimental groups.FAnimal weights (*n* = 4) were monitored during therapy experiments and expressed as mean ± SEM. Source data are available online for this figure.

**Figure EV4:**
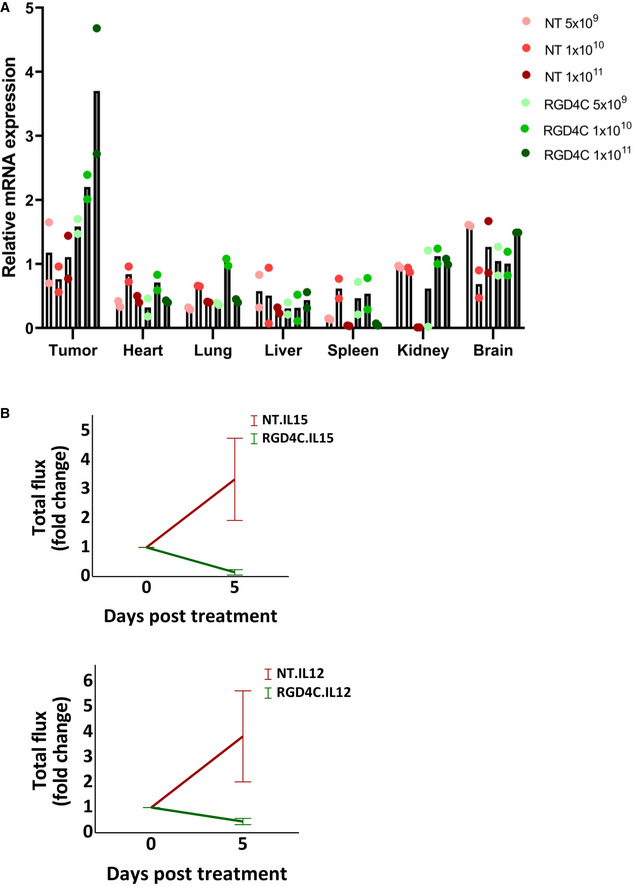
TPA particle biodistribution and targeted cytokine therapy ACohorts of immunocompetent BALB/c mice with established subcutaneous tumours derived from CT26.CL25 cells (*n* = 6 mice), were systemically administered with increasing doses 5 × 10^9^, 1 × 10^10^ and 1 × 10^11^ TU/mouse, of targeted (RGD4C) or non‐targeted (NT) TPA.*IL15* ^*IgK*^. *IL15* ^*IgK*^ gene expression was assessed by quantification of the *IL15* ^*IgK*^ mRNA in tumours and healthy tissues after 5 days. Data shown are representative of one experiment, *n* = 2 technical replicates.BGraphs showing tumour growth after targeted delivery of *IL15* ^*IgK*^ or *IL12*. Tumour‐bearing mice injected with non‐targeted (NT) TPA particles were included as controls. Data from Figs 6D and 7C were used to show the mean of total tumour luminescence from day 0 to day 5 ± SEM (*n* = 4 mice per group). Source data are available online for this figure.

To ascertain the *in vivo* efficacy of transmorphic particles in delivering *IL15* ^*IgK*^ in immunotherapy, we investigated the biodistribution of TPA.*IL15* ^*IgK*^ after intravenous administration in immunocompetent BALB/c mice bearing subcutaneous CT26.CL25 tumours (Fig 6A). We first tested TPA at 5 × 10^10^ TU/mouse and found a significant expression of *IL15* mRNA by RGD4C.TPA.*IL15* ^*IgK*^ in tumours (Fig 6A). In contrast, *IL15* expression in the healthy tissues tested (heart, lung, liver, spleen, kidney and brain) was insignificant and similar to that of the non‐targeted (control) particles that did not induce any *IL15* mRNA expression in both the tumours and other vital organs (Fig 6A). Moreover, we performed biodistribution with increasing doses of TPA and observed a significant dose‐dependent expression of *IL15* mRNA by RGD4C.TPA.*IL15* ^*IgK*^ in tumours at 5 × 10^9^, 1 × 10^10^ and 1 × 10^11^ TU/mouse (Fig EV4A). Yet, again and with all the doses tested, the non‐targeted TPA did not produce any *IL15* mRNA expression in the tumours and normal organs, and *IL15* expression in the healthy tissues from the RGD4C.TPA.*IL15* ^*IgK*^ group, was similar to that of non‐targeted TPA (Fig EV4A).

We also evaluated the safety of RGD4C.TPA.*IL15* ^*IgK*^ by performing a dose escalation using the four doses above, 5 × 10^9^, 1 × 10^10^, 5 × 10^10^ and 1 × 10^11^ TU/mouse, then measuring the serum levels of the lactate dehydrogenase (LDH) as a surrogate marker for cellular cytotoxicity and cytolysis (Fig 6B). We found no increase in LDH expression in the sera of mice receiving all the doses tested, in both RGD4C and non‐targeted treatment groups, when compared with the untreated group.

**Figure EV5:**
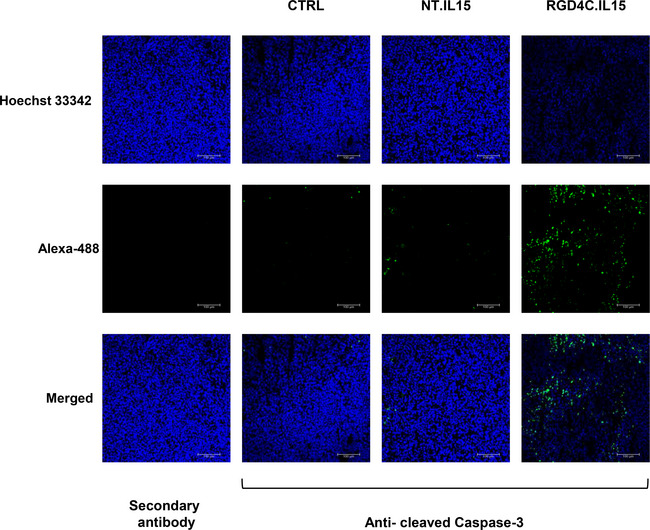
Immunostaining of tumour sections using an anti‐cleaved caspase‐3 antibody Tumour sections from tumour‐bearing mice (*n* = 6) following single TPA.*IL15* ^*IgK*^ dose treatment, 5 × 10^10^ TU/mouse, of targeted (RGD4C.*IL15*) or non‐targeted (NT.*IL15*). Untreated mice were used as controls (CTRL). Tumour sections incubated with the secondary antibody alone were also included as negative controls. Hoechst 33342 was used to stain the cell nuclei. Scale bar, 100 μm. Source data are available online for this figure.

After evaluating the efficacy of gene expression and safety profile of our targeted RGD4C.TPA particles carrying *IL15* ^*IgK*^, we performed solid tumour immunotherapy with single and repeated intravenous dosing of RGD4C.TPA.*IL15* ^*IgK*^ to mice bearing subcutaneous CT26.CL25 tumours labelled with the firefly *Luc* gene, allowing the tumour response to be monitored using BLI of *Luc* (Fig 6C). We selected 5 × 10^10^ particles per mouse as the dose to be administered to mice upon detection of tumours. We also included control groups of tumour‐bearing mice administered with vehicle or non‐targeted vector. Following a single intravenous dose, BLI of mice showed a remarkable and significant regression in tumour size and tumour bioluminescence in the group of mice treated with the RGD4C.TPA.*IL15* ^*IgK*^ as early as day 5 post‐treatment (Fig 6C). The non‐targeted and the untreated groups showed a significant increase in the tumour mass. Moreover, quantification of the total tumour luminescence signals indicated

a substantial reduction of viable tumour cells and tumour growth in the targeted treatment group compared to controls (Figs 6D and EV4B). We also examined the tumours for apoptosis by evaluating expression of the cleaved caspase‐3 which marks apoptotic cells. Interestingly, the data revealed a high level of apoptosis and a decrease in the number of cells in tumours following targeted cytokine therapy with RGD4C.TPA.*IL15* ^*IgK*^ as compared to non‐targeted TPA.*IL15* ^*IgK*^‐treated tumours or control untreated group (Fig EV5).

Next, to demonstrate the long‐term effects of transmorphic particle‐guided immunotherapy using IL15^IgK^, we carried out repeated dosing with 3 vector doses on days 0, 1 and 4 and continued to monitor the mice post‐treatment (Fig 6E). We found complete eradication of tumours with more than 1‐year survival in 50% of the mice treated with RGD4C.TPA.*IL15* ^*IgK*^, indicating a curative response, while all mice receiving the non‐targeted particle died 50 days after initial TPA administration (Fig 6E). In addition, we observed no changes in weight of mice, suggesting that this repeated dosing can be given safely (Fig 6F). Our results indicate a high therapeutic potential of IL15^IgK^ immunotherapy in combination with targeted transmorphic particles as an effective delivery method.

### Immune profiling of tumours after treatment with TPA. *IL15* ^*IgK*^ particles

**Figure 7:**
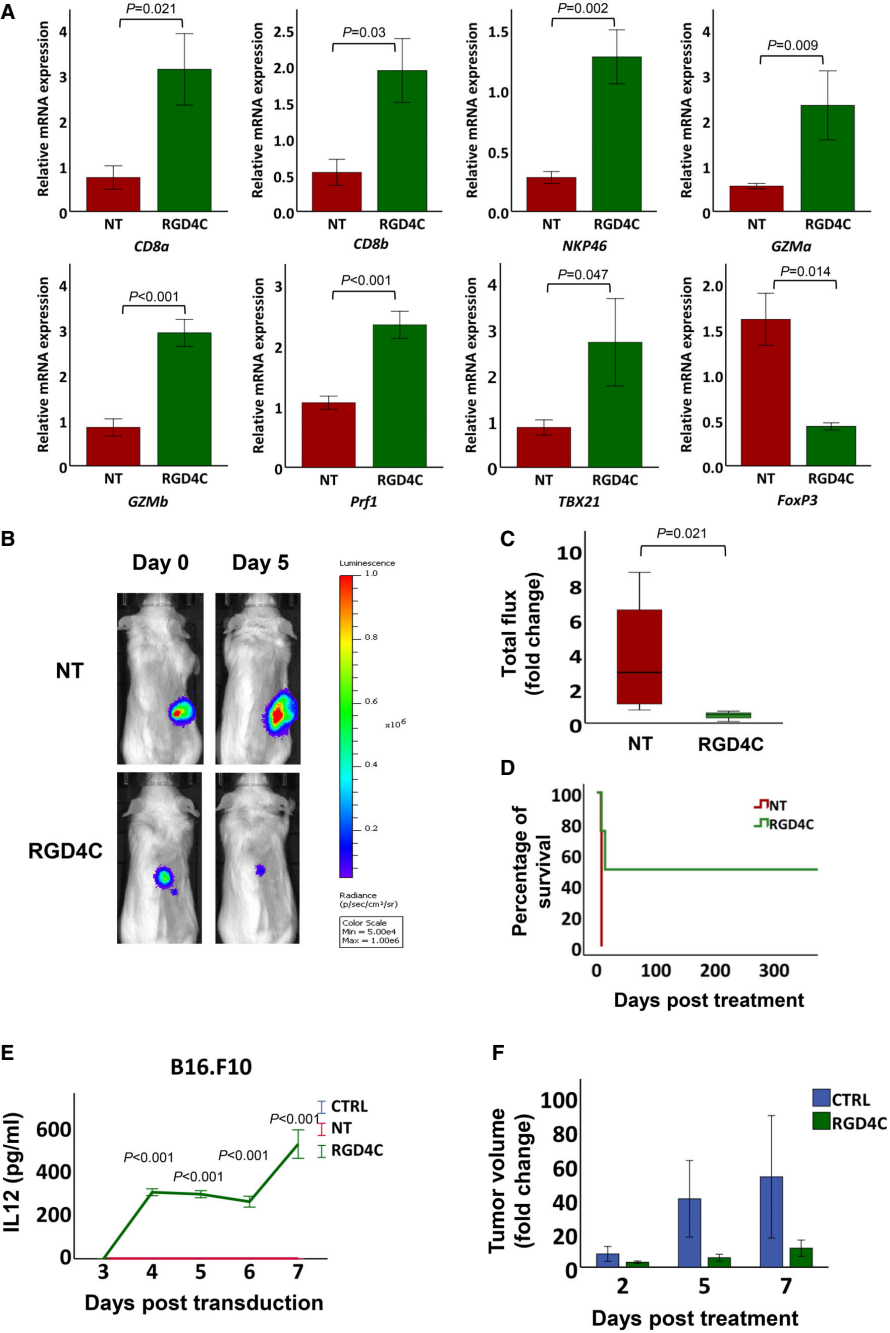
Tumour immune profiling after RGD4C.TPA.*IL15* ^*IgK*^ treatment and immunotherapy with RGD4C.TPA encoding *IL12* AThe immune profile of CT26.CL25 tumours was investigated using RT‐qPCR at day 5 after TPA.*IL15* ^*IgK*^ administration. A panel of immunological mRNA transcripts were selected for analysis and included CD8a and CD8b as markers for CD8^+^ T cell population; NKp46 for NK cell population. Markers for cytotoxic cell‐killing by CD8^+^ T cells (GZMa, GZMb) and by NK cells (Prf1) were also analysed. Expression of TBX21 was evaluated as a marker for the Th_1_ response mediated by interferon gamma (IFN‐γ), and FoxP3 for T_reg_ cells. Data shown are representative of *n* = 5 mice and expressed as mean ± SEM. Nonparametric Mann–Whitney test was used for GZMb and TBX2 data analysis, and independent *t*‐test to analyse data of the other markers.BTPA particles encoding *IL12* were used to treat immunocompetent BALB/c mice bearing subcutaneous CT26.CL25 tumours labelled with a *Luc* reporter gene. Representative tumour‐bearing mice at day 5 post‐treatment with targeted RGD4C.TPA.*IL12* (RGD4C) or non‐targeted TPA.*IL12* (NT).CAnalysis of total flux change in tumours, *n* = 4 mice, after IL12 immunotherapy. The central band represents the median of the data. The boxes upper and lower lines represent quartile 3 and quartile 1 of the data respectively. The whiskers represent the maximum and minimum outliers of the data. Nonparametric Mann–Whitney test was used for data analysis.DKaplan–Meier curves showing survival benefit for tumour‐bearing mice (n = 4) from two experimental groups.ESerial quantification of IL12 in media of B16.F10 cells treated with RGD4C.TPA.*IL12* or controls. Data are representative of one experiment, *n* = 3 biological repeats and shown as mean ± SD. Experiments were repeated three times. One‐way ANOVA with Tukey's HSD test was used for data analysis.FRGD4C.TPA.*IL12* immunotherapy of subcutaneous B16.F10 tumours in C57BL/6 mice (*n* = 4). Data are shown as mean ± SEM independent *t*‐test was used for data analysis. Tumour volume changes are shown over time. Source data are available online for this figure.

To check for post‐treatment effects and investigate the mechanisms of IL15^IgK^‐mediated anti‐tumour immunotherapy, we obtained a detailed analysis of the immune infiltration into CT26.CL25 tumours recovered 5 days after therapy. Thus, we investigated the expression of gene markers of immune cells within the tumour microenvironment. Our results showed that tumour expression of *CD8a* and *CD8b* (main markers of CD8^+^ T cells) and *NKp46* (a main marker of natural killer, NK cells) increased significantly in the group of mice receiving RGD4C.TPA.*IL15* ^*IgK*^ immunotherapy, as compared to the control group treated with non‐targeted TPA.*IL15* ^*IgK*^ (Fig 7A). These findings suggest a differentiation and proliferation of CD8^+^ T and NK cells, which are the main effector cells in anti‐tumour immunity and IL15‐mediated immunotherapy (Uzhachenko & Shanker, 2019). Next, to validate activation of the CD8^+^ T and NK cells, gene expression analysis of *GZMa* (granzyme a), *GZMb* (granzyme b) and *Prf1* (perforins) showed a significant increase in tumours of mice treated with RGD4C.TPA.*IL15* ^*IgK*^ particles (Fig 7A). This suggests that targeted tumour expression of the new hybrid IL15^Igk^ results in tumour enrichment with CD8^+^ T and NK cells, and subsequent stimulation of their cytolytic activity against cancer (Steel *et al*, 2012).

The RGD4C.TPA.*IL15* ^*IgK*^ treatment also increased expression of the *TBX21* gene encoding a T‐box transcription factor protein 21 (Fig 7A). TBX21 is crucial for T helper 1 cell (Th_1_) transformation and controls expression of the hallmark Th_1_ cytokine, interferon gamma (IFN‐γ; Miller & Weinmann, 2010). Since the release of IFN‐γ and IL2 from Th_1_ cells can promote differentiation of naïve CD8^+^ T cells into cytotoxic T cells, Th_1_ cells are considered the most prominent subtype for anti‐tumour immunity (Tay *et al*, 2020).

It is well known that tumour cells can evade host anti‐tumour strategies and develop an immunosuppressive environment to avoid eradication. The central component of this is regulatory T cells (T_reg_), which suppress anti‐tumour responses within the tumour microenvironment (Tay *et al*, 2020). The immunosuppressive function of T_reg_ is regulated and maintained by expression of the transcription factor forkhead box protein P3 (FoxP3) (Lu *et al*, 2017; Owen *et al*, 2019). Thus, we analysed expression of the *FoxP3* gene as a functional marker of Treg cells and found a significant decrease in its expression in tumours of mice administered with targeted RGD4C.TPA.*IL15* ^*IgK*^ particles (Fig 7A). These findings confirm that IL15^IgK^ does not stimulate immunosuppressive functions within the tumour tissue, and further emphasises its promising effect in anti‐cancer immunotherapy.

### Tumour immunotherapy using transmorphic particles bearing IL12

To further validate the observed anti‐tumour effects in immunocompetent mice, we performed immunotherapy in solid tumours using IL12. During the past two decades, IL12 has emerged as one of the most potent cytokines in mediating anti‐tumour activity in a variety of preclinical models (Berraondo *et al*, 2018). Still, the majority of clinical trials involving IL12 failed to show sustained anti‐tumour responses in cancer patients due to the lack of tumour selectivity resulting in systemic toxicity (Nguyen *et al*, 2020). We tested the therapeutic efficacy of transmorphic particles encoding IL12 in immunocompetent BALB/c mice with established subcutaneous CT26.CL25 tumours labelled with the *Luc* gene. Tumour‐bearing mice were treated with three doses of TPA.*IL12*, on days 0, 2 and 5 post‐treatment. BLI of *Luc* showed systemic administrations of targeted RGD4C.TPA.*IL12* not only inhibited tumour growth, but also resulted in substantial regression in tumour size as compared to treatment initiation on day 0 (Fig 7B). In contrast, the tumours continued to grow larger in mice given the non‐targeted particles. Furthermore, evaluation of bioluminescent signals in tumours showed a significant reduction in tumour viability of over 10‐fold at day 5 post‐treatment using RGD4C‐targeted particles compared to the control group receiving non‐targeted particles (Figs 7C and EV4B). We also evaluated the impact of therapeutic efficacy on survival of mice with CT26.CL25 tumours and found that treatment with RGD4C.TPA.*IL12* increased the survival of tumour‐bearing mice (Fig 7D). For instance, there was no animal survival at day 8 post‐treatment in the control non‐targeted group. Remarkably, more than 50% of mice treated with targeted RGD4C.TPA.*IL12* had survived and were cured as a result of complete response to treatment and tumour elimination (Fig 7D).

To rule out the possibility of any species‐ or tumour‐specific activity attributed to IL12, we carried out an investigation using the mouse B16.F10 melanoma model that establishes syngeneic melanoma in C57BL/6 mice. The B16.F10 are well‐recognised murine models used for preclinical testing of immunotherapeutic approaches. First, to confirm that the IL12 can be delivered and secreted efficiently using transmorphic particles, we transduced B16.F10 cells *in vitro* using RGD4C.TPA.*IL12* or non‐targeted NT.TPA.*IL12*. Cells treated with RGD4C‐targeted particles expressed and released IL12 in the culture medium from day 4 and peaking at day 7 post‐transduction at 500 pg/ml (Fig 7E). The non‐targeted group and the control untreated group did not show any detectable IL12 in the culture medium. Next, we performed immunotherapy in C57BL/6 mice bearing subcutaneous B16.F10 tumours. Upon repeated intravenous administrations of RGD4C.TPA.*IL12*, on days 0, 2 and 5, similar to BALB/c mice bearing CT26.CL25 tumours, we observed a reduction in tumour volume by 4‐ and 5‐fold at days 5 and 7 post‐treatment compared to the control (untreated) group respectively (Fig 7F). This is important since many immunotherapy studies failed to stop the growth of these very aggressive tumours. Together, the *in vivo* results from both immunocompetent models indicate that transmorphic particles can be used to deliver native IL12 for efficacious solid tumour immunotherapy.

## Discussion

The efficacy and safety of cancer immunotherapy can be dramatically improved when combined with an effective delivery strategy. We combined the advantages of eukaryotic and prokaryotic viral gene delivery through transmorphic packaging of the rAAV DNA using a tumour‐targeted filamentous phage capsid. The resulting TPA particles are able to efficiently target and deliver immunotherapeutic genes to the tumour cells *in vitro* and *in vivo*, while sparing healthy tissues. Here, we demonstrate its potential to overcome a critical barrier in cancer immunotherapy by enabling controlled and safe systemic delivery of anti‐tumour cytokines to the site of pathology.

The positive response to immunotherapy observed in our study is attributed to the successful production of TPA particles for targeted cytokine gene transfer. Particle expression systems that utilise helper phages have long been established, but the overwhelming contamination of helper phage and insufficient yield of desired particles for therapeutic applications have hindered meaningful development (Chasteen *et al*, 2006). TPA particles produced from our optimised system are currently able to generate a final yield of up to 1 × 10^13^ TU/μl particles for every 1 l of bacteria culture medium used. Moreover, we were able to control the amount of helper phage, which can easily be brought down to 0% by density gradient ultracentrifugation or FPLC (Monjezi *et al*, 2010). The TPA platform is able to provide astonishing particle yield, as well as enhanced *in vitro* and pre‐clinical gene transfer unseen before by any bacteriophage‐derived vectors. Using a prokaryotic manufacturing system to produce TPA particles confers advantages in cost, time and scalability. In addition, completely eliminating the phage genome allows for easier molecular cloning and manipulation.

Gene transfer occurs over a series of extracellular and intracellular processes, and the reduction of particle size confers the efficiency of TPA particles over existing phage‐derived vectors. In each transduction process we investigated, the reduction in particle size of TPA was found to be critical to its efficacy. TPA particles are more likely, than AAVP, to diffuse in the extracellular space, including the extracellular matrix and thus become more bioavailable for binding to the cell surface, resulting in a much higher rate of particle internalisation, particularly through clathrin‐mediated endocytosis (Stoneham *et al*, 2012; Yata *et al*, 2015). We previously reported limited AAVP diffusion, through the‐ extracellular matrix, as a physical barrier to AAVP entry into cells (Yata *et al*, 2015). Subsequently, the rAAV payload of TPA is delivered to the nucleus more quickly and efficiently compared to larger vectors carrying the full phage sequence. Indeed, the higher efficacy of transduction is consistent with the gene expression data gathered in HEK293 cells, as well as primary tumour cells, tumour stem cells and primary tumour spheres. With length being the only physical dissimilarity, we concluded that TPA particles are able to induce efficient gene expression through greater diffusion, internalisation and particle‐guided gene delivery to the nucleus as a function of reduced particle size and genome, and elimination of all the phage structural genes.

In this study, we demonstrate that TPA particles provide a novel method for targeted cytokine gene delivery and cancer immunotherapy by inducing selective cytokine production in solid tumours. Our results show TPA particles are an efficient platform for systemic and targeted delivery of cytokines to tumours while sparing healthy tissues using a panel of three cytokine genes. It is particularly important to note that we observed no off‐target transduction in healthy tissues, even in the liver where bacteriophages are usually subject to clearance by the reticuloendothelial system (Geier *et al*, 1973). TPA particles are able to induce a complete therapeutic response over repeated administrations in immunocompetent animals without observable diminishing efficacy often seen in mammalian viral vectors (Riviere *et al*, 2006). These findings are highly important because targeted cytokine delivery has been an insurmountable barrier for clinical translation. The inability to target cytokines to the tumour site is thought to be a primary cause of side effects, particularly compounded by repeated administrations, due to the pleiotropic effects that cytokines have on multiple tissues. When combined with the induction of cell‐mediated immunity, it is advantageous that TPA are able to induce immunity at the site of the tumour, which involves key anti‐tumour players such as CD8^+^ T cells and NK cells, without stimulating cells involved in tumour immunosuppression (Ribatti, 2017). This conclusion is further substantiated by our immune profiling results of tumour tissue after targeted delivery of *IL15* ^*IgK*^, showing deactivation of immunosuppression and activation of anti‐tumour immune cells. Our targeted approach for cytokine delivery allows immunotherapy to occur at the lesion while other tissues, which are known cytokine targets, are spared.

Transmorphic Phage/AAV particles offer a resolution from limitations currently present in both gene delivery and immunotherapy. A determining feature is its target specificity, which is derived from its prokaryotic capsid proteins that the mammalian immune system is better able to tolerate. By using the RGD4C targeting moiety on the cell surface, we were able to deliver cytokine genes to the tumour without any detectable expression in healthy tissue/organs (Paoloni *et al*, 2009; Przystal *et al*, 2019). The RGD4C ligand binds mainly to α_v_β_3_ integrin heterodimer, but also a lesser level to α_v_β_5_ (Przystal *et al*, 2019). We and collaborators have published a large body of work reporting the tumour selectivity of RGD4C.phage‐based vectors in mice, rats and pet dogs (Hajitou *et al*, 2006; Paoloni *et al*, 2009; Tandle *et al*, 2009; Przystal *et al*, 2013, 2019; Yuan *et al*, 2013), with no gene delivery detected in healthy tissues. We also reported that a panel of normal human primary cells from different histological origins do not express or have very low expression of the α_v_β_3_ and α_v_β_5_ integrin receptors of RGD4C (Przystal *et al*, 2019). Notably, this very low integrin profile did not translate into gene delivery to normal cells by RGD4C.phage‐derived vectors. Finally, the RGD4C peptide was used in cancer patients to target the α_v_β_3_ integrin receptor in human cancer (Reardon *et al*, 2008).

In addition, delivering cytokine genes and the ability to induce sustained expression is not currently achievable in immunotherapy through the use of recombinant cytokines. By using TPA particles, we are able to overcome the physical stability and circulating half‐life of cytokines since packaged TPA particles, like phage, are stable at ambient temperature for months.

In our investigation, we explored the use of three cytokines, TNFα, IL12 and IL15, all of which involve cell‐mediated tumour killing and provide promising results when TPA is used as a delivery method (Otani *et al*, 1999; Johansson *et al*, 2012; Waldmann *et al*, 2020). Clinical trial data have previously shown the potential for cytokine‐based immunotherapy in cancer patients, but were unable to address their short plasma half‐life, susceptibility to renal clearance and side effects caused by lack of specificity. To address these needs, we also designed and characterised two secreted isoforms of human TNFα^IL2^ and IL15^IgK^ to enhance their bioavailability, in addition to packaging them into TPA particles. Using our TPA platform, we were able to target the delivery of cytokine genes through the systemic circulation. This in turn enabled higher bioactivity at the target site, providing higher therapeutic benefit compared to the established native isoforms of TNFα and IL15 with no observable toxicity in our preclinical studies. Using a phage capsid to encapsidate therapeutic AAV transgene cassettes in small particles of _~_7 nm in diameter should also allow TPA to evade particle clearance by the mammalian immune system, which has consistently been an observed hindrance in viral vectors (Shirley *et al*, 2020). As a result, we constantly observed a reduction in tumour volume over repeated administrations, indicating accumulating efficacy in both immunosuppressed and immunocompetent mouse models.

One might speculate that a broad range of current cancer immunotherapies will be made safer and more effective against solid tumours in combination with the TPA particles. For example, systemic delivery of TPA constructs carrying transgene cassettes encoding antibodies can be used for targeted antibody production for immune checkpoint blockade. Ligands for CAR T cells, such as CD19, can be selectively expressed in solid tumours by the TPA to guide CAR T cell therapy. Tumour‐targeted TPA can also be used in cancer vaccines to express foreign antigens in tumours and tag them for destruction. The intrinsic nature of phage in attracting antigen‐presenting cells (APC) should also help promote cellular immunity against antigens it delivers through being a naturally potent adjuvant (Manoutcharian *et al*, 2001; Frenkel & Solomon, 2002). Phage‐based particles can also be internalised by nearby cells, digested and displayed to major histocompatibility complex (MHC) class I and class II antigen‐processing pathways (Gaubin *et al*, 2003). Finally, because of their reduced size, TPA particles can potentially be used to deliver multiple cytokines or immunotherapies in a single particle by accommodating various AAV transgene cassettes.

In the context of immunotherapy, we believe TPA particles possess considerable potential for clinical translation due to their cost advantages and safety compared to currently available cytokine treatments and viral vectors. Immunotherapy is extremely expensive, often requiring the conjugation of the cytokines to a monoclonal antibody to drive target specificity. The resulting treatment programmes may cost over 100,000 US dollars per quality‐adjusted life‐years (Verma *et al*, 2018). On the contrary, our targeted TPA particles are manufactured using a modified and widely used bacteriophage system, do not require costly raw materials (such as purified DNA plasmids), and can be produced at commercial scales with GMP standards (Kotin, 2011). In terms of safety, bacteriophages have a positive and lengthy historic track record as antibiotics and food additives approved by the United States FDA (Moye *et al*, 2018). A safety study of phage‐guided cancer therapy in pet dogs has also shown promising safety data, as well as tolerance by the immune system after repeated administration (Paoloni *et al*, 2009).

In summary, although the field of cancer immunotherapy as a whole is advancing at a rapid pace, the design of novel delivery technologies is still in its emergent stages. Our novel TPA particles represent an important technological advancement in systemic gene delivery capable of safe and efficient targeted cytokine administration.

## Materials and Methods

### Design and construction of TPA particles

Transmorphic Phage/AAV particles were designed and constructed using the rAAV2‐GFP plasmid from a commercially available laboratory‐scale AAV production kit (Cell BioLabs, USA) and a modified helper phage based on the filamentous M13KO7 helper phage (NEB, UK). The rAAV2 plasmid was used as the basis for the construction of all TPA plasmids by replacing GFP DNA sequence with the gene of interest. To package the rAAV transgene into TPA particles, we designed M13KO7 bearing the double cyclic RGD4C insertion mutation on the pIII minor coat protein gene using DNA primers, and amplified by Q5 High‐Fidelity DNA Polymerase (NEB) according to the manufacturer's recommended protocol. The amplicon was subsequently self‐ligated using quick ligase (NEB), and transformed into chemically competent Mix&Go Competent TG1 *E. coli* (Zymo Research, USA) according to the manufacturer's recommended protocol. Transformed cells were plated on Tryptone yeast extract (TYE) top agar supplemented with 50 μg/ml kanamycin and incubated for 18 h at 37°C. Colonies were picked and cultured in 2xYT medium supplemented with 50 μg/ml kanamycin, shaking at 200 rpm overnight for another 18 h. Bacterial outgrowth was then extracted for DNA using the plasmid miniprep kit (Qiagen, UK), loaded on gel electrophoresis to confirm the correct plasmid size, and sent for sequencing (Eurofins, Germany) of the pIII gene to identify positive clones. Mutation‐bearing M13KO7‐positive clones are referred to as RGD4C.M13KO7.

To create stocks of M13KO7 or RGD4C.M13KO7, bacterial clones carrying the helper phage genome were cultured in 500 ml 2xYT supplemented with 50 μg/ml kanamycin then incubated shaking at 200 rpm 37°C overnight for an additional 18 h. The culture was then subjected to phage purification.

To construct TPA.*TNFα* ^*IL2*^, TPA.*IL15* ^*IgK*^ and TPA.*IL12* plasmids, molecular cloning was conducted using restriction enzyme digestions (NEB), Q5 High‐Fidelity polymerase chain reaction (NEB) and Quick Ligation™ Kit (NEB). Briefly, sense and antisense strands of *IL2* and *IgK* signal peptides (Table EV1) flanked by *BamHI* and *EcoRI* restriction sites (Life Technologies, UK) were annealed at a gradually decreased temperature from 95°C to 4°C. *TNFα* and *IL15* sequences flanked by *EcoRI* and *SalI* restriction sites were amplified from pUNO1‐*hTNFα* (Invivogen, France) and pUNO1‐*mIL15* (Invivogen) plasmids, respectively. *IL12* flanked by *AscI* and *SalI* sites was amplified from UNO1‐*mIL12* (Invivogen). Primers used for these PCR are listed in Table EV1. *TNFα* ^*IL2*^, *IL15* ^*IgK*^ or *IL12* sequences were subsequently ligated to TPA backbone and transformed into *DH5α* competent bacteria. Final constructs were confirmed by restriction enzyme digestions and DNA sequencing. The correct constructs were then transformed into Mix&Go Competent *TG1 E. coli* (Zymo Reseach) to produce TPA particles.

### TPA particle production

To produce TPA particles, we transformed Mix&Go Competent TG1 *E. coli* (Zymo Reseach) using the TPA plasmid. Clones were screened using a plasmid miniprep kit (Qiagen) and subjected to DNA gel electrophoresis to confirm the correct size. Positive clones were grown in 50 ml 2xYT medium (shaking at 200 rpm, 37°C) supplemented with 100 μg/ml ampicillin to log phase (OD_600_ = 0.4–0.6) and superinfected by adding 1 × 10^12^ TU RGD4C.M13KO7 or M13KO7 phage, mixed by swirling, and incubated (37°C) at stationary for 20 min. The infected culture was added to 450 ml fresh 2xYT medium supplemented with 100 μg/ml ampicillin and 50 μg/ml kanamycin, and incubated overnight for 18–20 h, shaking at 200 rpm, 37°C.

### TPA particle purification

The overnight culture, 500 ml, was immediately centrifuged at 3,000–6,000 *g* for 15–30 min at 4°C, the supernatant was collected and the bacterial pellet was discarded. Ice‐cold PEG/NaCl solution (20% Polyethylene glycol 8000, 2.5 M NaCl in deionised water) was added to achieve 30% v/v final concentration and swirled for gentle mixing. The supernatant/PEG/NaCl mixture was left at 4°C overnight, then centrifuged at 10,000 *g* for 30 min at 4°C. The supernatant was discarded, and the pellet was resuspended in 10 ml phosphate‐buffered saline (PBS). Next, the solution was again precipitated with PEG/NaCl solution at 4°C for another overnight. After centrifugation, the supernatant was discarded and the phage pellet was resuspended in 1 ml PBS. Another round of centrifugation at 10,000 *g* was performed for 10 min to remove the bacterial debris. The crude phage supernatant was collected and was then filtered through 0.45 μm PVDF membrane filter to remove fine bacterial debris. The TPA particles are stably stored at 4°C; however, we regularly monitor their titre after a long storage. To date, we have stored TPA at 4°C for up to 2 years without any significant drop in their titre.

### AAVP vector construction and production

AAVP vectors were constructed by using the same rAAV2‐GFP plasmid used for TPA construction (Cell Biolabs). Insertion of rAAV2 DNA into the fUSE5 phage genome and construction of RGD4C.fUSE5 displaying the RGD4C peptide, to produce the tumour‐targeted RGD4C.AAVP, were carried out using molecular cloning as we previously described in detail (Hajitou *et al*, 2007). Production of AAVP viral vectors occurred in K91 *E. coli* grown to log phase (OD_600_ = 1.6–2.0) in LB broth in the presence of kanamycin (100 μg/ml) by incubation at 37°C and shaking at 250 rpm since the host K91 *E. coli* are resistant to kanamycin. In each production, 2 ml of the K91 *E. coli* culture was infected by mixing with 1 × 10^9^ TU of AAVP vectors and incubated for 20 min at room temperature. The infected bacteria were added to 450 ml LB broth in the presence of kanamycin (100 μg/ml) and tetracycline (40 μg/ml), selection marker in the AAVP construct, then incubated at 37°C with shaking at 250 rpm for 18–20 h. AAVP vectors were purified using the same protocol as TPA particles.

### Titration of TPA particles and AAVP vectors

Transmorphic Phage/AAV particles were quantified in prokaryotic hosts. Serial dilutions of TPA particles were made in PBS and used to infect TG1 *E. coli* grown to log phase in 2xYT medium, which were subsequently incubated at 37°C. After 20 min incubation at 37°C in a water bath, the particles/bacteria mixture was mixed well again, and was plated on solid agar medium with selective antibiotics. TPA particles contain an ampicillin‐resistant gene, so TYE top agar with 100 μg/ml ampicillin was used. Whereas the helper phage particle contains a kanamycin‐resistant gene, so TYE top agar with 50 μg/ml kanamycin was used. The bacteria were plated on TYE top agar in the presence of ampicillin to determine the concentration of TPA particles and kanamycin to determine the concentration of helper phage present in the sample by colony counting.

AAVP vectors were similarly quantified by serial dilutions in PBS; however, K91 *E. coli* was grown to log phase in LB broth in the presence of kanamycin (100 μg/ml) and used for colony counting on LB plates in the presence of both kanamycin and tetracycline (100 and 40 μg/ml respectively).

All agar plates were incubated at 37°C for 18–20 h until colonies were visible. Both TPA and AAVP are expressed as bacterial transducing units TU/μl.

### Transmission electron microscopy of transmorphic particles

Carbon film‐coated copper mesh grids were glow discharged to induce hydrophilicity. AAVP vectors and TPA particles were applied on the grids, left to incubate for 10–15 min and removed by blotting on absorbent paper. The grids were then washed with sterile‐filtered deionised water, blotted on absorbent paper twice and dried for 15 min. 1% uranyl acetate solution was applied on the grid to negatively stain the particles for 30 s, and subsequently washed twice with sterile‐filtered deionised water and dried. The grids were imaged using a scanning electron microscope (JEOL JEM‐2010, UK) and analysed using ImageJ software (Schneider *et al*, 2012).

### Cell culture

HEK293, CT26.CL25, B16.F1, B16.F10 and CTLL‐2 were purchased from the American Type Culture Collection (ATCC). Biopsy‐derived GBM (SEBTA003) cell line was provided by Prof. Geoff Pilkington, University of Portsmouth. HEK293, SEBTA003, B16.F1 and B16.F10 were grown in Dulbecco's Modified Eagle's Medium (DMEM) supplemented with 10% Fetal Bovine Serum (FBS) and 1% penicillin and streptomycin. CT26.CL25 was cultured in RPMI1640 medium supplemented with 10 mM HEPES, 1 mM sodium pyruvate, 0.1 mM non‐essential amino‐acids and 10% FBS. Cytotoxic T lymphocyte, CTLL‐2, cell line was grown in RPMI1640 supplemented with 2 mM L‐glutamine, 1 mM pyruvate, 10% FBS and 10 IU/ml IL2.

The human primary GBM, HSJD‐GBM‐001 (GBM001), cell line was established by Dr. Angel Carcaboso at the Hospital Sant Joan de Deu (HSJD), Barcelona, Spain, from a biopsy‐derived GBM of a female cancer patient and were grown as previously reported (Monje *et al*, 2011; Przystal *et al*, 2019). Primary glioblastoma‐derived stem cell line G26 (provided by Prof. Steven Pollard from the Edinburgh Brain Cancer, Scotland) was obtained from a patient tumour and was maintained in serum‐free cultures on laminin‐coated plate, using neural stem (NS) cell media supplemented with EGF and FGF‐2 to a final concentration of 10 ng/ml as reported (Pollard, 2013). To establish and culture tumour spheres from GBM001, the tumour cells were resuspended in tumour stem medium (TSM) consisting of neurobasal medium 50% (v/v), D‐MEM F12 50%(v/v), B27 (Life Technologies), human FGF (20 ng/ml), human EGF (20 ng/ml, Peprotech UK), human PDGF‐AA (10 ng/ml, Peprotech), human PDGF‐BB (10 ng/ml) and heparin (20 μg/ml, Sigma‐Aldrich UK).

All the cells used in this study were either authenticated by the supplier ATCC or by collaborators who provided them. Upon reception of these cell lines, they are first tested and cleared for mycoplasma contamination. Cells in tissue culture undergo regular monthly mycoplasma testing (Lonza, UK).

### Transduction of adherent cells and tumour spheres using TPA particles and AAVP vectors

Adherent cells were seeded in well plates/tissue culture dishes of preferred sizes to achieve 70–80% confluence 48 h after seeding. On the day of transduction, the average number of cells per well/dish culture was determined and used for calculating the amount of TPA particles or AAVP vectors to add to the cells. The transduction mixture is then prepared by diluting the appropriate amount of the particles stock solution in serum‐free medium, then mixing thoroughly. The recommended volume of transduction mixture used per well/dish is the minimum volume required to completely cover the cell monolayer. To transduce cells, medium was discarded and the transduction mixture was added to the cells for 6–12 h at 37°C 5% CO_2_ before supplementation with an equal volume of complete medium. After 24 h, the whole medium was discarded and replaced with fresh medium. The transduced cells were maintained in culture until analysis.

To transduce the tumour spheres, cells were suspended in spheroid medium and left to grow for 2 days. On the day of transduction, cells in the spheres were counted to determine the appropriate amount of TPA or AAVP to include into the transduction medium. Next, the transduction mixtures were added directly to the sphere cultures which were then mixed on an orbital shaker at 50 rpm for 10 min. The transduced spheres were left to grow in a cell culture incubator without replacing the medium, until analysis of transgene expression.

### Quantification of secreted luciferase expression in the culture medium

At specified timepoints post‐transduction with TPA.*Lucia* or AAVP.*Lucia* carrying the *Lucia* DNA sequence, 10 μl of culture medium was collected from wells and transferred in to opaque 96‐microwell plates. Luciferase activity was quantified using QUANTI‐Luc; a luciferase substrate was prepared according to the manufacturer's protocol (Invivogen) and added to the microwell plate. Luciferase activity was measured using a GloMax Discover Microplate Luminometer (Promega, UK). For these experiments, the culture medium was not changed at any timepoint.

### Internalisation assay for TPA and AAVP particles

Eukaryotic cells were seeded in 48‐well plates and treated with AAVP or TPA particles according to the above‐outlined transduction protocol. At 2‐ and 4‐h timepoints, cells were immediately placed on ice to suspend internalisation and washed three times with ice‐cold PBS. Cells were subsequently trypsinised, centrifugated and immediately fixed with 4% paraformaldehyde at pH = 7.2 for 15 min at room temperature after mixing. The fixed cells were permeabilised and blocked using ice‐cold 0.1% saponin and 2% BSA in PBS for 30 min, then washed twice in ice‐cold 0.1% saponin and 1% BSA in PBS for 30 min. Cells were stained using a rabbit anti‐bacteriophage antibody (Sigma, USA, B7786), diluted 1:500 in 0.1% saponin and 1% BSA in PBS for 1 h at room temperature, and washed three times in 0.1% saponin and 1% BSA in PBS then centrifuged at 1,500 *g* at 4°C. Cells were then secondarily stained using a goat anti‐rabbit IgG antibody conjugated with AlexaFluor‐647 (Life Technologies, A27040) diluted at 1:250 in 0.1% saponin and 1% BSA in PBS for 1 h at room temperature and washed three further times using 0.1% saponin and 1% BSA in PBS and centrifuged at 1,500 *g* at 4°C. FACS was performed using a BD FACScalibur Flow Cytometer equipped with an argon‐ion (488 nm) and red‐diode (635 nm) laser. The mean fluorescence intensity was measured for at least 10,000 gated cells per triplicate well. Analysis of the results was performed using FlowJo (BD, USA) software.

### Nuclei and cytosol extraction

Following treatment with TPA or AAVP particles, the cells were washed twice with ice‐cold PBS and harvested with ice‐cold Nuclei EZ lysis buffer kit (Sigma, UK) then left on ice for 5 min with brief mixing. The harvested samples were centrifuged at 500 *g* for 5 min at 4°C. Next, the supernatant was collected for the cytosolic fraction, while the nuclei pellet was resuspended/vortexed briefly, washed twice with ice‐cold Nuclei EZ lysis buffer and collected by centrifugation at 500 *g* for 5 min at 4°C. The nuclei fraction was resuspended in TE buffer.

### Quantitative PCR analysis of AAV ITR elements

Quantification of the ITR cis elements in the cytoplasm and nucleus of cells treated with TPA or AAVP particles was carried out using a modified protocol from previous reports (Aurnhammer *et al*, 2012; Tsafa *et al*, 2016, 2020). The nuclear and cytosolic fractions isolated from TPA or AAVP‐transduced cells were diluted (1 in 20) in TE buffer, then used as templates for qPCR analysis by using primers specifics for the ITR elements (Table EV2). Real‐time PCR was performed using SYBR green master mix (Applied Biosystem, UK) and fluorescence was measured on an Agilent AriaMx instrument (Agilent Technologies, UK). The PCR protocol was set as follows: 50°C for 2 min, 98°C for 3 min, 40 cycles of 98°C for 15 s, 60°C for 30 s and melting curve quantification from 65°C to 95°C. Serial dilutions of TPA or AAVP DNA plasmids with known amounts were used as quantitative standards. The relative ITR element levels were calculated using the $2^{-\Delta \Delta C_{T}}$ method.

### Phage particle fluorochrome labelling

TPA and AAVP particles were labelled with FITC. Fifty microlitres of particles (5 × 10^11^ TU, total) was added into 200 μl containing 5 mg/ml FITC (Sigma, UK), then mixed by rotating for 1 h at room temperature in the dark. Subsequently, the particles were precipitated by addition of PEG/NaCl 25–30% total concentration at 4°C, overnight. The solution was centrifuged at 16,200 *g* for 15 min to obtain the pellet of particles. The pellet was resuspended in 250 μl PBS and re‐precipitated with PEG/NaCl until free FITC was completely removed. Finally, FITC‐conjugated phage particles were resuspended in PBS and titres were quantified using the *E. coli* bacterial infection and colony counting method.

### Matrigel diffusion assay

Two hundred microlitres of Matrigel from Engelbreth‐Holm‐Swarm murine sarcoma (Sigma, UK) at 2.5 mg/ml was added to a 48‐well plate, then transferred at 37°C. In the meantime, FITC‐labelled particles were prepared at a concentration of 5 μg/ml. Five microlitres of each particle solution was pipetted in a gel‐loading pipette tip, which was inserted at a fixed position into the Matrigel and left to diffuse. Fluorescent images were taken using a fluorescent microscope (Nikon Eclipse TE2000U, Japan) and analysed by Openlab imaging software at 5 and 30 min intervals thereafter.

### Phage trans‐well diffusion assay

A Matrigel Matrix was thawed at 4°C overnight before used. The Matrigel, final concentration of 5 mg/ml, was prepared in 300 μl of DMEM and loaded into each well of 24 trans‐well plate, then incubated at 37°C for at least 30 min. TPA or AAVP particles, 1 × 10^10^ TU, were diluted in 300 μl of PBS and added into each well of the coated Matrigel Matrix trans‐well containing 600 μl of PBS, then incubated at 37°C. Subsequently, the diffused particle solution was collected every day from the lower compartment for 7 days. The number of phage particles was quantified using the *E. coli* bacterial infection and counting colony titration method.

### Enzyme‐linked immunosorbent assay (ELISA)

Production of IL15, IL12 and TNFα in the supernatant or cell lysates after transduction was quantified using a mouse IL15 duoset ELISA (R&D systems, UK), mouse IL12 (p70) ELISA MAX™ Deluxe set (Biolegend, UK) and human ELISA MAX™ Standard Set TNFα kit (Biolegend) respectively. The assays were performed according to the manufacturer's instructions.

### Endotoxin removal

The endotoxins contaminating the TPA and AAVP preps were removed using Pierce™ High Capacity Endotoxin Removal Spin Columns (Thermo Fisher Scientific, UK). Briefly, 2 ml of phage particles solution was loaded into the equilibrated columns and incubated overnight with gentle end‐to‐end mixing at 4°C. The phage solution was eluted by centrifugation at 500 *g* for 1 min. Endotoxin level was subsequently measured using Limulus Amebocyte Lysate (LAL) PYROGENT™ Plus Single Test Vials (Lonza).

### IL15 bioactivity assay

Stimulation of CTLL‐2 proliferation was used to test the bioactivity of IL15. Cell proliferation assay was performed using CellTiter 96® AQueous One Solution Cell Proliferation Assay (Promega). The protocol was modified from a previous report (Soman *et al*, 2009). Briefly, CTLL‐2 was washed with Hank's balanced salt solution (HBSS) and incubated in RPMI 1640 supplemented with 10% FBS for 4 h at 37°C/5% CO_2_ during which 100 μl of conditioned media from TPA‐transduced tumour cells were added to 96‐well plate. After 4 h incubation, 5 × 10^4^ CTLL‐2 were seeded into the 96‐well plate with the condition media and incubated for a further 48 h. Next, 20 μl/well of CellTiter 96® AQueous One Solution was added and incubated for 4 h at 37°C/5% CO_2_ before adding 10% SDS and reading the plate at 490 nm using a VersaMAX tunable microplate reader (Molecular Devices, UK).

### *In vivo* biodistribution of TPA‐mediated transgene expression

Tumour‐bearing mice received different systemic doses of TPA particles and were sacrificed at various time points, as indicated in the results section; then tumours, healthy organs and serum from each mouse were collected. Snap frozen tumour samples and organs were homogenised with ceramic bead and RNA was subsequently extracted using PureLink RNA mini kit (Thermo Fisher Scientific) according to the manufacturer's instructions. RNA concentration was measured by a nanodrop spectrophotometer. Total RNA was converted to cDNA using High Capacity cDNA Reverse Transcription Kits (Thermo Fisher Scientific) following the manufacturer's protocol. qPCR was performed to quantify TNFα and IL15 transgenes by using Powerup SYBR Green Master Mix (Thermo Fisher Scientific). The primers were purchased from Life Technologies, and are listed in Table EV2. The level of gene expression in tumours and healthy organs was calculated using the $2^{-\Delta \Delta C_{T}}$ method. *GAPDH* expression (the house‐keeping gene) was used as internal control.

### Immunotherapy in tumour‐bearing mice

To establish tumour‐bearing mouse models for immunotherapy, tumour cells were first labelled with the *Luc* reporter gene by using a lentiviral Lenti‐*GFP‐Luc* vector (System Biosciences, UK) to generate the GBM001‐*Luc* and CT26.CL25‐*Luc*. Then, GBM001‐*Luc* and CT26.CL25‐*Luc* were subcutaneously implanted to mice (1 × 10^6^ cells/mouse). BLI of *Luc* was thus used as a simple way to detect tumours, monitor tumour growth and viability and evaluate tumour response to therapy. Tumour‐bearing mice were intravenously administered through the tail vein with targeted or control non‐targeted TPA particles carrying cytokines transgenes at a dose of 5 × 10^10^ TU/mouse or otherwise indicated in the manuscript. To monitor luciferase expression, mice were anaesthetised and administered with 100 mg/kg of d‐luciferin (Gold Biotechnology, USA), then imaged by using the *In Vivo* Imaging System (IVIS 100; Calliper Life Sciences/Perkin Elmer, UK). A region of interest was defined manually over the tumours to measure the signal intensities recorded as total photon counts per second per cm^2^ (p/s/cm2 /sr; Hajitou *et al*, 2007; Hajitou *et al*, 2006). Tumour growth was evaluated by measuring the tumour luciferase signal intensity overtime. At the end of experiment, the tumour and healthy tissues were harvested. Sera from mice were also collected to evaluate any systemic toxicity by quantitative measurement of the LDH using the CytoTox 96® colorimetric Cytotoxicity Assay (Promega).

Experiments involving living mice were carried out according to the Institutional and Home Office Guidelines, and under a granted Home Office‐issued project licence number PPL 70/7035. The project licence was first reviewed and approved by the Animal Welfare and Ethical Review Body (AWERB committee) at Imperial College London, before its final review and approval by the UK Home Office. Immunodeficient female NOD/SCID mice, adult 5–7 weeks, were purchased from Charles River, UK, and immunocompetent C57BL/6 or BALB/c female mice, adult 5–7 weeks, from Envigo, UK. NOD/SCID mice are more prone to infection by opportunistic pathogens and were thus housed behind appropriate barrier housing; irradiated diet and bedding were also provided to reduce the risk of infection.

### Immunofluorescence staining of cleaved caspase‐3

Tumour frozen sections, 6 μm, were allowed to air dry for 3 h at room temperature, then fixed in 4% paraformaldehyde for 15 min and washed three times with Tris‐buffer saline (TBS). Next, sections were blocked with 5% normal goat serum in TBS with 0.3% Triton‐X for 1 h at room temperature, and incubated with an anti‐cleaved caspase‐3 rabbit monoclonal antibody (Cell Signalling Technology, UK), 1:500, in TBS with 1% BSA and 0.3% Triton‐X overnight at 4°C. Thereafter, sections were washed 3 times with TBS and 0.3% Triton‐X, then incubated with an anti‐rabbit IgG‐alexa Fluor‐488 secondary antibody (Invitrogen, UK), 1:1,000, and Hoechst33342 (Invitrogen), 1:2,000, for 1 h at room temperature. Slides were mounted with Prolong Gold anti‐fade mounts (Invitrogen). Immunofluorescence images were acquired using Leica SP8 TCS confocal fluorescence microscope (Leica, UK) with LASX software version 3.7.4.23463.

### Statistical analysis

Statistical analysis was performed using IBM SPSS statistics software version 25. *P* values were generated by either independent *t*‐test, one‐way ANOVA with Tukey's honestly significant difference (HSD) *post hoc* test, or two‐way ANOVA. Mann–Whitney and Kruskal–Wallis tests were also used as a nonparametric test. Animal survival curves were generated using Kaplan–Meier method (Kaplan–Meier survival fractions). *P* values were considered significant at *P* < 0.05 and represented as follows: *P < 0.05, **P ≤ 0.01, ***P ≤ 0.001.

*In vitro* experiments were designed to ensure 5% significance level and a minimum of 80% power. Experiments were performed in triplicates and repeated, at least twice, increasing the total number of samples, which is sufficient for a statistical power above 80%.

For animal experimentation, we have applied the principles of the 3Rs (Replacement, Reduction and Refinement). However, the experiments were replicated, which increased the total number of animals and subsequently the statistical power. We also took into account that some animals are terminated before the end point. Indeed, animals with tumour necrosis/ulceration (characterised as tissue loss) are culled and excluded in line with our Home Office‐issued project licence. Moreover, blinding was applied throughout the animal studies. Upon tumour detection, tumour‐bearing mice were randomly assigned to groups. Then, imaging of tumour‐bearing mice was done, cage by cage, blindingly and by applying similar parameters for all cages.

## Data availability

This study includes no data deposited in external repositories.

## Author contributions

**Paladd Asavarut:** Data curation; software; formal analysis; validation; investigation; visualization; writing––original draft; writing––review and editing; methodology. **Sajee Waramit:** Data curation; software; formal analysis; validation; methodology. **Keittisak Suwan:** Resources; data curation; software; formal analysis; validation; investigation; methodology. **Gert J K Marais:** Data curation; validation; methodology. **Aitthiphon Chongchai:** Data curation; methodology. **Surachet Benjathummarak:** Data curation; methodology. **Mariam Al‐Bahrani:** Data curation; methodology. **Paula Vila‐Gomez:** Data curation; methodology. **Matthew Williams:** Resources; funding acquisition. **Prachya Kongtawelert:** Resources; funding acquisition. **Teerapong Yata:** Conceptualization; methodology. **Amin Hajitou:** Conceptualization; resources; data curation; formal analysis; supervision; funding acquisition; investigation; methodology; project administration; writing––original draft; writing––review and editing.

In addition to the CRediT author contributions listed above, the contributions in detail are:

PA, SW and KS designed, optimised and performed the majority of the *in vitro* and *in vivo* experiments then analysed the data. GJKM, AC, SB, MA‐B and PV‐G helped with the *in vitro* experiments and data analysis. TY assisted with the initial *in vitro* experiments and early design of the technology. MW and PK supported the project and assisted with the funding. AH designed the experiments, conceived and supervised the study, analysed the data, performed *in vivo* experiments, ensured financial support for the entire project and drafted the manuscript with PA, SW and KS.

## Disclosure and competing interests statement

PA, SW, KS, MA‐B, TY and AH are inventors on two patent applications related to this work. These patents have been licenced to M13 Therapeutics, USA. The remaining authors declare that they have no conflict of interest.

**The paper explained**

**Problem**

Since the 1980s, anti‐tumour cytokines have been used in cancer immunotherapy. Yet, a fundamental problem remains the control over immune‐activating cytokines at the target site, which can have fatal effects on the host. Cytokine‐encoding genes have thus been developed to express cytokines in cancer cells only; however, gene delivery is hindered by the lack of tumour‐selective vectors and issues linked to repeated administrations.

**Results**

We established a unique prokaryotic viral‐based approach of intravenous gene delivery to specifically target tumours by using the filamentous M13 bacteriophage that infects bacteria only. In this vector, the M13 phage capsid was engineered to target cancer and deliver therapeutic transgene expression cassettes carrying genes encoding interleukin IL12, IL15 and tumour necrosis factor alpha. These phage‐derived particles proved to be an efficient platform for safe and selective systemic delivery of cytokines to solid tumours, while avoiding healthy tissues in preclinical models of human and murine tumours. Moreover, administration of particles in immunocompetent animals could be repeated and resulted in tumour eradication and complete response in more than 50% of the mice.

**Impact**

The newly developed phage‐derived particles can be applied for selective and efficient cytokine therapy. These findings are important since targeted cytokine delivery has been a major barrier for clinical translation. Given that cytokines have already been tested in cancer patients, and that phage safety in humans is increasingly established, the clinical efficacy of this targeted cytokine therapy to treat solid tumours is promising. Moreover, the treatment is administered through the systemic route, and thus could be applied both for localised and metastatic disease.

## For more information

i) https://patents.google.com/patent/WO2017077275A1/en
ii) https://www.m13tx.com/
iii) https://pubmed.ncbi.nlm.nih.gov/30888467/
iv) http://www.biosci.missouri.edu/smithgp/PhageDisplayWebsite/PhageDisplayWebsiteindex.html

## Supporting information



Expanded View Figures PDF

Table EV1

Table EV2

Source Data for Expanded View

Source Data for Figure 2

Source Data for Figure 3

Source Data for Figure 4

Source Data for Figure 5

Source Data for Figure 6

Source Data for Figure 7

## References

[emmm202115418-bib-0001] Anderson DB , Laquerre S , Ghosh K , Ghosh HP , Goins WF , Cohen JB , Glorioso JC (2000) Pseudotyping of glycoprotein D‐deficient herpes simplex virus type 1 with vesicular stomatitis virus glycoprotein G enables mutant virus attachment and entry. J Virol 74: 2481–2487 10666285 10.1128/jvi.74.5.2481-2487.2000 PMC111736

[emmm202115418-bib-0002] Arap W , Kolonin MG , Trepel M , Lahdenranta J , Cardo‐Vila M , Giordano RJ , Mintz PJ , Ardelt PU , Yao VJ , Vidal CI *et al* (2002) Steps toward mapping the human vasculature by phage display. Nat Med 8: 121–127 11821895 10.1038/nm0202-121

[emmm202115418-bib-0003] Aurnhammer C , Haase M , Muether N , Hausl M , Rauschhuber C , Huber I , Nitschko H , Busch U , Sing A , Ehrhardt A *et al* (2012) Universal real‐time PCR for the detection and quantification of adeno‐associated virus serotype 2‐derived inverted terminal repeat sequences. Hum Gene Ther Methods 23: 18–28 22428977 10.1089/hgtb.2011.034

[emmm202115418-bib-0004] Berraondo P , Etxeberria I , Ponz‐Sarvise M , Melero I (2018) Revisiting Interleukin‐12 as a cancer immunotherapy agent. Clin Cancer Res 24: 2716–2718 29549160 10.1158/1078-0432.CCR-18-0381

[emmm202115418-bib-0005] Berraondo P , Sanmamed MF , Ochoa MC , Etxeberria I , Aznar MA , Perez‐Gracia JL , Rodriguez‐Ruiz ME , Ponz‐Sarvise M , Castanon E , Melero I (2019) Cytokines in clinical cancer immunotherapy. Br J Cancer 120: 6–15 30413827 10.1038/s41416-018-0328-y PMC6325155

[emmm202115418-bib-0006] Bradbury AR , Sidhu S , Dubel S , McCafferty J (2011) Beyond natural antibodies: the power of in vitro display technologies. Nat Biotechnol 29: 245–254 21390033 10.1038/nbt.1791 PMC3057417

[emmm202115418-bib-0007] Burg MA , Jensen‐Pergakes K , Gonzalez AM , Ravey P , Baird A , Larocca D (2002) Enhanced phagemid particle gene transfer in camptothecin‐treated carcinoma cells. Cancer Res 62: 977–981 11861367

[emmm202115418-bib-0008] Campbell S , Suwan K , Waramit S , Aboagye EO , Hajitou A (2018) Selective inhibition of histone deacetylation in melanoma increases targeted gene delivery by a bacteriophage viral vector. Cancers (Basel) 10: 125 10.3390/cancers10040125 PMC5923380 29690504

[emmm202115418-bib-0009] Cao JX , Wang H , Gao WJ , You J , Wu LH , Wang ZX (2020) The incidence of cytokine release syndrome and neurotoxicity of CD19 chimeric antigen receptor‐T cell therapy in the patient with acute lymphoblastic leukemia and lymphoma. Cytotherapy 22: 214–226 32305113 10.1016/j.jcyt.2020.01.015

[emmm202115418-bib-0010] Chasteen L , Ayriss J , Pavlik P , Bradbury AR (2006) Eliminating helper phage from phage display. Nucleic Acids Res 34: e145 17088290 10.1093/nar/gkl772 PMC1693883

[emmm202115418-bib-0011] Chertova E , Bergamaschi C , Chertov O , Sowder R , Bear J , Roser JD , Beach RK , Lifson JD , Felber BK , Pavlakis GN (2013) Characterization and favorable in vivo properties of heterodimeric soluble IL‐15.IL‐15Ralpha cytokine compared to IL‐15 monomer. J Biol Chem 288: 18093–18103 23649624 10.1074/jbc.M113.461756 PMC3689953

[emmm202115418-bib-0012] Conforti F , Pala L , Bagnardi V , De Pas T , Martinetti M , Viale G , Gelber RD , Goldhirsch A (2018) Cancer immunotherapy efficacy and patients' sex: a systematic review and meta‐analysis. Lancet Oncol 19: 737–746 29778737 10.1016/S1470-2045(18)30261-4

[emmm202115418-bib-0013] Darrow JJ (2019) Luxturna: FDA documents reveal the value of a costly gene therapy. Drug Discov Today 24: 949–954 30711576 10.1016/j.drudis.2019.01.019

[emmm202115418-bib-0014] Day CP , Merlino G , Van Dyke T (2015) Preclinical mouse cancer models: a maze of opportunities and challenges. Cell 163: 39–53 26406370 10.1016/j.cell.2015.08.068 PMC4583714

[emmm202115418-bib-0015] Dean R , Jensen I , Cyr P , Miller B , Maru B , Sproule DM , Feltner DE , Wiesner T , Malone DC , Bischof M *et al* (2021) An updated cost‐utility model for onasemnogene abeparvovec (Zolgensma(R)) in spinal muscular atrophy type 1 patients and comparison with evaluation by the Institute for Clinical and Effectiveness Review (ICER). J Mark Access Health Policy 9: 1889841 33708361 10.1080/20016689.2021.1889841 PMC7919869

[emmm202115418-bib-0016] Donnelly A , Yata T , Bentayebi K , Suwan K , Hajitou A (2015) Bacteriophage mediates efficient gene transfer in combination with conventional transfection reagents. Viruses 7: 6476–6489 26670247 10.3390/v7122951 PMC4690874

[emmm202115418-bib-0017] Foust KD , Wang X , McGovern VL , Braun L , Bevan AK , Haidet AM , Le TT , Morales PR , Rich MM , Burghes AH *et al* (2010) Rescue of the spinal muscular atrophy phenotype in a mouse model by early postnatal delivery of SMN. Nat Biotechnol 28: 271–274 20190738 10.1038/nbt.1610 PMC2889698

[emmm202115418-bib-0018] Frenkel D , Solomon B (2002) Filamentous phage as vector‐mediated antibody delivery to the brain. Proc Natl Acad Sci USA 99: 5675–5679 11960022 10.1073/pnas.072027199 PMC122830

[emmm202115418-bib-0019] Gaubin M , Fanutti C , Mishal Z , Durrbach A , De Berardinis P , Sartorius R , Del Pozzo G , Guardiola J , Perham RN , Piatier‐Tonneau D (2003) Processing of filamentous bacteriophage virions in antigen‐presenting cells targets both HLA class I and class II peptide loading compartments. DNA Cell Biol 22: 11–18 12590733 10.1089/104454903321112451

[emmm202115418-bib-0020] Geier MR , Trigg ME , Merril CR (1973) Fate of bacteriophage lambda in non‐immune germ‐free mice. Nature 246: 221–223 4586796 10.1038/246221a0

[emmm202115418-bib-0021] Grimm D , Zhou S , Nakai H , Thomas CE , Storm TA , Fuess S , Matsushita T , Allen J , Surosky R , Lochrie M *et al* (2003) Preclinical in vivo evaluation of pseudotyped adeno‐associated virus vectors for liver gene therapy. Blood 102: 2412–2419 12791653 10.1182/blood-2003-02-0495

[emmm202115418-bib-0022] Hajitou A , Rangel R , Trepel M , Soghomonyan S , Gelovani JG , Alauddin MM , Pasqualini R , Arap W (2007) Design and construction of targeted AAVP vectors for mammalian cell transduction. Nat Protoc 2: 523–531 17406616 10.1038/nprot.2007.51

[emmm202115418-bib-0023] Hajitou A , Trepel M , Lilley CE , Soghomonyan S , Alauddin MM , Marini FC 3rd , Restel BH , Ozawa MG , Moya CA , Rangel R *et al* (2006) A hybrid vector for ligand‐directed tumor targeting and molecular imaging. Cell 125: 385–398 16630824 10.1016/j.cell.2006.02.042

[emmm202115418-bib-0024] Johansson A , Hamzah J , Payne CJ , Ganss R (2012) Tumor‐targeted TNFalpha stabilizes tumor vessels and enhances active immunotherapy. Proc Natl Acad Sci USA 109: 7841–7846 22547817 10.1073/pnas.1118296109 PMC3356673

[emmm202115418-bib-0025] Josephs SF , Ichim TE , Prince SM , Kesari S , Marincola FM , Escobedo AR , Jafri A (2018) Unleashing endogenous TNF‐alpha as a cancer immunotherapeutic. J Transl Med 16: 242 30170620 10.1186/s12967-018-1611-7 PMC6119315

[emmm202115418-bib-0026] Kennedy LB , Salama AKS (2020) A review of cancer immunotherapy toxicity. CA Cancer J Clin 70: 86–104 31944278 10.3322/caac.21596

[emmm202115418-bib-0027] Kia A , Yata T , Hajji N , Hajitou A (2013) Inhibition of histone deacetylation and DNA methylation improves gene expression mediated by the adeno‐associated virus/phage in cancer cells. Viruses 5: 2561–2572 24153059 10.3390/v5102561 PMC3814604

[emmm202115418-bib-0028] Kotin RM (2011) Large‐scale recombinant adeno‐associated virus production. Hum Mol Genet 20: R2–R6 21531790 10.1093/hmg/ddr141 PMC3095058

[emmm202115418-bib-0029] Kutateladze M , Adamia R (2010) Bacteriophages as potential new therapeutics to replace or supplement antibiotics. Trends Biotechnol 28: 591–595 20810181 10.1016/j.tibtech.2010.08.001

[emmm202115418-bib-0030] Kwiatkowska A , Nandhu MS , Behera P , Chiocca EA , Viapiano MS (2013) Strategies in gene therapy for glioblastoma. Cancers (Basel) 5: 1271–1305 24202446 10.3390/cancers5041271 PMC3875940

[emmm202115418-bib-0031] Larocca D , Burg MA , Jensen‐Pergakes K , Ravey EP , Gonzalez AM , Baird A (2002) Evolving phage vectors for cell targeted gene delivery. Curr Pharm Biotechnol 3: 45–57 11883506 10.2174/1389201023378490

[emmm202115418-bib-0032] Larocca D , Jensen‐Pergakes K , Burg MA , Baird A (2001) Receptor‐targeted gene delivery using multivalent phagemid particles. Mol Ther 3: 476–484 11319907 10.1006/mthe.2001.0284

[emmm202115418-bib-0033] Larocca D , Kassner PD , Witte A , Ladner RC , Pierce GF , Baird A (1999) Gene transfer to mammalian cells using genetically targeted filamentous bacteriophage. FASEB J 13: 727–734 10094933 10.1096/fasebj.13.6.727

[emmm202115418-bib-0034] Larocca D , Witte A , Johnson W , Pierce GF , Baird A (1998) Targeting bacteriophage to mammalian cell surface receptors for gene delivery. Hum Gene Ther 9: 2393–2399 9829538 10.1089/hum.1998.9.16-2393

[emmm202115418-bib-0035] Lu L , Barbi J , Pan F (2017) The regulation of immune tolerance by FOXP3. Nat Rev Immunol 17: 703–717 28757603 10.1038/nri.2017.75 PMC5793224

[emmm202115418-bib-0036] Manoutcharian K , Gevorkian G , Cano A , Almagro JC (2001) Phage displayed biomolecules as preventive and therapeutic agents. Curr Pharm Biotechnol 2: 217–223 11530876 10.2174/1389201013378671

[emmm202115418-bib-0037] Medler J , Wajant H (2019) Tumor necrosis factor receptor‐2 (TNFR2): An overview of an emerging drug target. Expert Opin Ther Targets 23: 295–307 30856027 10.1080/14728222.2019.1586886

[emmm202115418-bib-0038] Miller SA , Weinmann AS (2010) Molecular mechanisms by which T‐bet regulates T‐helper cell commitment. Immunol Rev 238: 233–246 20969596 10.1111/j.1600-065X.2010.00952.x PMC2988494

[emmm202115418-bib-0039] Monje M , Mitra SS , Freret ME , Raveh TB , Kim J , Masek M , Attema JL , Li G , Haddix T , Edwards MS *et al* (2011) Hedgehog‐responsive candidate cell of origin for diffuse intrinsic pontine glioma. Proc Natl Acad Sci USA 108: 4453–4458 21368213 10.1073/pnas.1101657108 PMC3060250

[emmm202115418-bib-0040] Monjezi R , Tey BT , Sieo CC , Tan WS (2010) Purification of bacteriophage M13 by anion exchange chromatography. J Chromatogr B Analyt Technol Biomed Life Sci 878: 1855–1859 10.1016/j.jchromb.2010.05.028 20538529

[emmm202115418-bib-0041] Moye ZD , Woolston J , Sulakvelidze A (2018) Bacteriophage applications for food production and processing. Viruses 10: 205 10.3390/v10040205 PMC5923499 29671810

[emmm202115418-bib-0042] Nguyen KG , Vrabel MR , Mantooth SM , Hopkins JJ , Wagner ES , Gabaldon TA , Zaharoff DA (2020) Localized Interleukin‐12 for cancer immunotherapy. Front Immunol 11: 575597 33178203 10.3389/fimmu.2020.575597 PMC7593768

[emmm202115418-bib-0043] Nissim A , Hoogenboom HR , Tomlinson IM , Flynn G , Midgley C , Lane D , Winter G (1994) Antibody fragments from a ‘single pot’ phage display library as immunochemical reagents. EMBO J 13: 692–698 7508862 10.1002/j.1460-2075.1994.tb06308.x PMC394860

[emmm202115418-bib-0044] Otani T , Nakamura S , Toki M , Motoda R , Kurimoto M , Orita K (1999) Identification of IFN‐gamma‐producing cells in IL‐12/IL‐18‐treated mice. Cell Immunol 198: 111–119 10648125 10.1006/cimm.1999.1589

[emmm202115418-bib-0045] Owen DL , Sjaastad LE , Farrar MA (2019) Regulatory T cell development in the thymus. J Immunol 203: 2031–2041 31591259 10.4049/jimmunol.1900662 PMC6910132

[emmm202115418-bib-0046] Paoloni MC , Tandle A , Mazcko C , Hanna E , Kachala S , LeBlanc A , Newman S , Vail D , Henry C , Thamm D (2009) Launching a novel preclinical infrastructure: Comparative oncology trials consortium directed therapeutic targeting of TNFα to cancer vasculature. PLoS ONE 4: e4972 19330034 10.1371/journal.pone.0004972 PMC2659423

[emmm202115418-bib-0047] Pasqualini R , Ruoslahti E (1996) Organ targeting in vivo using phage display peptide libraries. Nature 380: 364–366 8598934 10.1038/380364a0

[emmm202115418-bib-0048] Pollard SM (2013) In vitro expansion of fetal neural progenitors as adherent cell lines. Methods Mol Biol 1059: 13–24 23934830 10.1007/978-1-62703-574-3_2

[emmm202115418-bib-0049] Przystal JM , Umukoro E , Stoneham CA , Yata T , O'Neill K , Syed N , Hajitou A (2013) Proteasome inhibition in cancer is associated with enhanced tumor targeting by the adeno‐associated virus/phage. Mol Oncol 7: 55–66 22951279 10.1016/j.molonc.2012.08.001 PMC3553581

[emmm202115418-bib-0050] Przystal JM , Waramit S , Pranjol MZI , Yan W , Chu G , Chongchai A , Samarth G , Olaciregui NG , Tabatabai G , Carcaboso AM *et al* (2019) Efficacy of systemic temozolomide‐activated phage‐targeted gene therapy in human glioblastoma. EMBO Mol Med 11: e8492 30808679 10.15252/emmm.201708492 PMC6460351

[emmm202115418-bib-0051] Reardon DA , Nabors LB , Stupp R , Mikkelsen T (2008) Cilengitide: an integrin‐targeting arginine‐glycine‐aspartic acid peptide with promising activity for glioblastoma multiforme. Expert Opin Investig Drugs 17: 1225–1235 10.1517/13543784.17.8.1225 PMC2832832 18616418

[emmm202115418-bib-0052] Regulski K , Champion‐Arnaud P , Gabard J (2021) Bacteriophage manufacturing: from early twentieth‐century processes to current GMP. In Bacteriophages: Biology, Technology, Therapy, Harper DR , Abedon ST , Burrowes BH , McConville ML (eds), pp 699–729. Cham: Springer International Publishing

[emmm202115418-bib-0053] Ribatti D (2017) The concept of immune surveillance against tumors. The first theories. Oncotarget 8: 7175–7180 27764780 10.18632/oncotarget.12739 PMC5351698

[emmm202115418-bib-0054] Riley RS , June CH , Langer R , Mitchell MJ (2019) Delivery technologies for cancer immunotherapy. Nat Rev Drug Discov 18: 175–196 30622344 10.1038/s41573-018-0006-z PMC6410566

[emmm202115418-bib-0055] Riviere C , Danos O , Douar AM (2006) Long‐term expression and repeated administration of AAV type 1, 2 and 5 vectors in skeletal muscle of immunocompetent adult mice. Gene Ther 13: 1300–1308 16688207 10.1038/sj.gt.3302766

[emmm202115418-bib-0056] Russell S , Bennett J , Wellman JA , Chung DC , Yu ZF , Tillman A , Wittes J , Pappas J , Elci O , McCague S *et al* (2017) Efficacy and safety of voretigene neparvovec (AAV2‐hRPE65v2) in patients with RPE65‐mediated inherited retinal dystrophy: A randomised, controlled, open‐label, phase 3 trial. Lancet 390: 849–860 28712537 10.1016/S0140-6736(17)31868-8 PMC5726391

[emmm202115418-bib-0057] Santiago‐Ortiz JL , Schaffer DV (2016) Adeno‐associated virus (AAV) vectors in cancer gene therapy. J Control Release 240: 287–301 26796040 10.1016/j.jconrel.2016.01.001 PMC4940329

[emmm202115418-bib-0058] Schneider CA , Rasband WS , Eliceiri KW (2012) NIH image to ImageJ: 25 years of image analysis. Nat Methods 9: 671–675 22930834 10.1038/nmeth.2089 PMC5554542

[emmm202115418-bib-0059] Shirley JL , de Jong YP , Terhorst C , Herzog RW (2020) Immune responses to viral gene therapy vectors. Mol Ther 28: 709–722 31968213 10.1016/j.ymthe.2020.01.001 PMC7054714

[emmm202115418-bib-0060] Smith TL , Yuan Z , Cardo‐Vila M , Sanchez Claros C , Adem A , Cui MH , Branch CA , Gelovani JG , Libutti SK , Sidman RL *et al* (2016) AAVP displaying octreotide for ligand‐directed therapeutic transgene delivery in neuroendocrine tumors of the pancreas. Proc Natl Acad Sci USA 113: 2466–2471 26884209 10.1073/pnas.1525709113 PMC4780640

[emmm202115418-bib-0061] Soman G , Yang X , Jiang H , Giardina S , Vyas V , Mitra G , Yovandich J , Creekmore SP , Waldmann TA , Quinones O *et al* (2009) MTS dye based colorimetric CTLL‐2 cell proliferation assay for product release and stability monitoring of interleukin‐15: assay qualification, standardization and statistical analysis. J Immunol Methods 348: 83–94 19646987 10.1016/j.jim.2009.07.010 PMC2786060

[emmm202115418-bib-0062] Steel JC , Waldmann TA , Morris JC (2012) Interleukin‐15 biology and its therapeutic implications in cancer. Trends Pharmacol Sci 33: 35–41 22032984 10.1016/j.tips.2011.09.004 PMC3327885

[emmm202115418-bib-0063] Stoneham CA , Hollinshead M , Hajitou A (2012) Clathrin‐mediated endocytosis and subsequent Endo‐lysosomal trafficking of adeno‐associated virus/phage. J Biol Chem 287: 35849–35859 22915587 10.1074/jbc.M112.369389 PMC3476254

[emmm202115418-bib-0064] Tandle A , Hanna E , Lorang D , Hajitou A , Moya CA , Pasqualini R , Arap W , Adem A , Starker E , Hewitt S *et al* (2009) Tumor vasculature‐targeted delivery of tumor necrosis factor‐alpha. Cancer 115: 128–139 19090007 10.1002/cncr.24001 PMC8385542

[emmm202115418-bib-0065] Tay RE , Richardson EK , Toh HC (2020) Revisiting the role of CD4(+)T cells in cancer immunotherapy‐new insights into old paradigms. Cancer Gene Ther 28: 5–17 32457487 10.1038/s41417-020-0183-x PMC7886651

[emmm202115418-bib-0066] Tsafa E , Al‐Bahrani M , Bentayebi K , Przystal J , Suwan K , Hajitou A (2016) The natural dietary genistein boosts bacteriophage‐mediated cancer cell killing by improving phage‐targeted tumor cell transduction. Oncotarget 7: 52135–52149 27437775 10.18632/oncotarget.10662 PMC5239540

[emmm202115418-bib-0067] Tsafa E , Bentayebi K , Topanurak S , Yata T , Przystal J , Fongmoon D , Hajji N , Waramit S , Suwan K , Hajitou A (2020) Doxorubicin improves cancer cell targeting by filamentous phage gene delivery vectors. Int J Mol Sci 21: 7867 10.3390/ijms21217867 PMC7660303 33114050

[emmm202115418-bib-0068] Uzhachenko RV , Shanker A (2019) CD8(+) T lymphocyte and NK cell network: Circuitry in the cytotoxic domain of immunity. Front Immunol 10: 1906 31456803 10.3389/fimmu.2019.01906 PMC6700470

[emmm202115418-bib-0069] Verma V , Sprave T , Haque W , Simone CB 2nd , Chang JY , Welsh JW , Thomas CR Jr (2018) A systematic review of the cost and cost‐effectiveness studies of immune checkpoint inhibitors. J Immunother Cancer 6: 128 30470252 10.1186/s40425-018-0442-7 PMC6251215

[emmm202115418-bib-0070] Waehler R , Russell SJ , Curiel DT (2007) Engineering targeted viral vectors for gene therapy. Nat Rev Genet 8: 573–587 17607305 10.1038/nrg2141 PMC7097627

[emmm202115418-bib-0071] Wajant H , Pfizenmaier K , Scheurich P (2003) Tumor necrosis factor signaling. Cell Death Differ 10: 45–65 12655295 10.1038/sj.cdd.4401189

[emmm202115418-bib-0072] Waldmann TA , Dubois S , Miljkovic MD , Conlon KC (2020) IL‐15 in the combination immunotherapy of cancer. Front Immunol 11: 868 32508818 10.3389/fimmu.2020.00868 PMC7248178

[emmm202115418-bib-0073] Wurdinger T , Badr C , Pike L , de Kleine R , Weissleder R , Breakefield XO , Tannous BA (2008) A secreted luciferase for ex vivo monitoring of in vivo processes. Nat Methods 5: 171–173 18204457 10.1038/nmeth.1177 PMC2699561

[emmm202115418-bib-0074] Yata T , Lee EL , Suwan K , Syed N , Asavarut P , Hajitou A (2015) Modulation of extracellular matrix in cancer is associated with enhanced tumor cell targeting by bacteriophage vectors. Mol Cancer 14: 110 26037383 10.1186/s12943-015-0383-4 PMC4451735

[emmm202115418-bib-0075] Yata T , Lee KY , Dharakul T , Songsivilai S , Bismarck A , Mintz PJ , Hajitou A (2014) Hybrid nanomaterial complexes for advanced phage‐guided gene delivery. Mol Ther Nucleic Acids 3: e185 25118171 10.1038/mtna.2014.37 PMC4221597

[emmm202115418-bib-0076] Yuan Z , Syrkin G , Adem A , Geha R , Pastoriza J , Vrikshajanani C , Smith T , Quinn TJ , Alemu G , Cho H *et al* (2013) Blockade of inhibitors of apoptosis (IAPs) in combination with tumor‐targeted delivery of tumor necrosis factor‐alpha leads to synergistic antitumor activity. Cancer Gene Ther 20: 46–56 23154431 10.1038/cgt.2012.83 PMC3534156

[emmm202115418-bib-0077] Zincarelli C , Soltys S , Rengo G , Rabinowitz JE (2008) Analysis of AAV serotypes 1‐9 mediated gene expression and tropism in mice after systemic injection. Mol Ther 16: 1073–1080 18414476 10.1038/mt.2008.76

