# Supplementary material for: Systemically targeted cancer immunotherapy and gene delivery using transmorphic particles
Source: EMBO Mol Med. 2022 Jun 27;14(8):e15418. doi: 10.15252/emmm.202115418 (PMC9358398; doi:10.15252/emmm.202115418)
Supplement: Supplementary file 1 — Expanded View Figures PDF [file EMMM-14-e15418-s004.pdf]

Expanded View Figures

Figure EV1. Genetic maps of Transmorphic Phage/AAV, TPA and Adeno-associated Virus/Phage, AAVP.

A A schematic diagram of the TPA DNA encoding enhanced eGFP. TPA contains two origins of replication: pUC (high copy-number, in yellow), which enables double-stranded DNA replication in prokaryotic hosts, and f1 ori (phage origin of replication, in red), which enables single-stranded DNA replication and packaging into the phage capsid.

B A schematic diagram of the chimeric genome of AAVP encoding eGFP. AAVP contains the full genomic sequence of filamentous bacteriophage, and a transgene cassette from AAV-2 inserted in to an intergenomic region.

 © 2022 The Authors

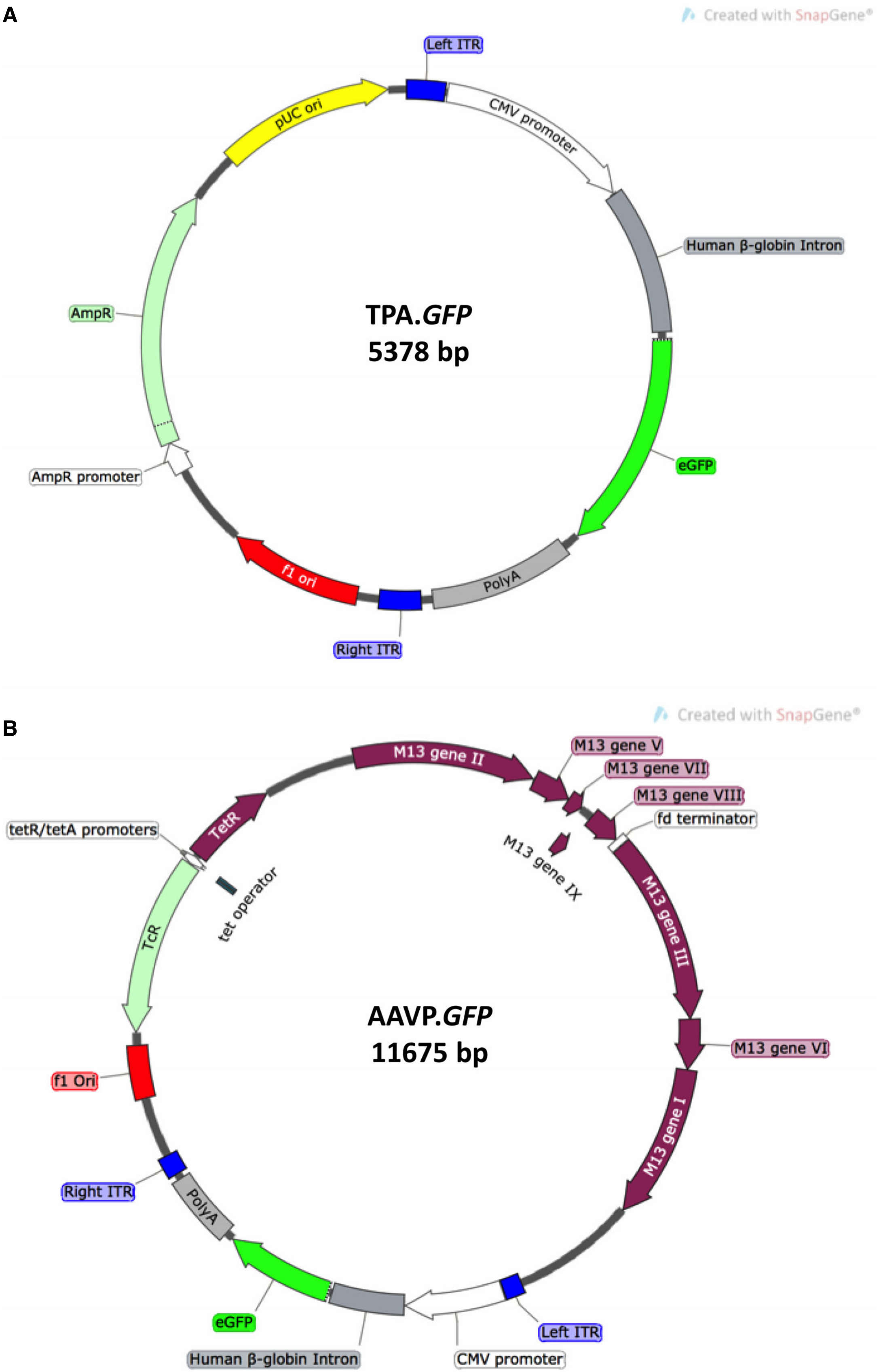
A
Created with SnapGene®
Left ITR
CMV promoter
Human β-globin Intron
eGFP
PolyA
Right ITR
f1 ori
AmpR promoter
AmpR
pUC ori
TPA.GFP
5378 bp
B
Created with SnapGene®
M13 gene II
M13 gene V
M13 gene VII
M13 gene VIII
fd terminator
M13 gene IX
M13 gene III
M13 gene VI
M13 gene I
Left ITR
CMV promoter
Human β-globin Intron
eGFP
PolyA
Right ITR
f1 Ori
TcR
tetR/tetA promoters
TetR
tet operator
AAVP.GFP
11675 bp

Figure EV1.

Figure EV2. Genetic map of M13KO7 helper phage bearing the RGD4C peptide for tumour targeting.

A A schematic diagram of the genome of M13KO7 helper phage used for packaging the TPA DNA (shown in Fig EV1) to produce non-targeted TPA particles. The M13KO7 genome contains a medium copy-number origin of replication (p15A, in yellow).

B A schematic diagram of the genome of RGD4C.M13KO7 helper phage used for packaging the TPA DNA (shown in Fig EV1) to produce the tumour-targeted RGD4C.TPA particles. The RGD4C coding sequence is inserted in-frame in to the M13 gene III, which encodes the pIII minor coat proteins.

© 2022 The Authors

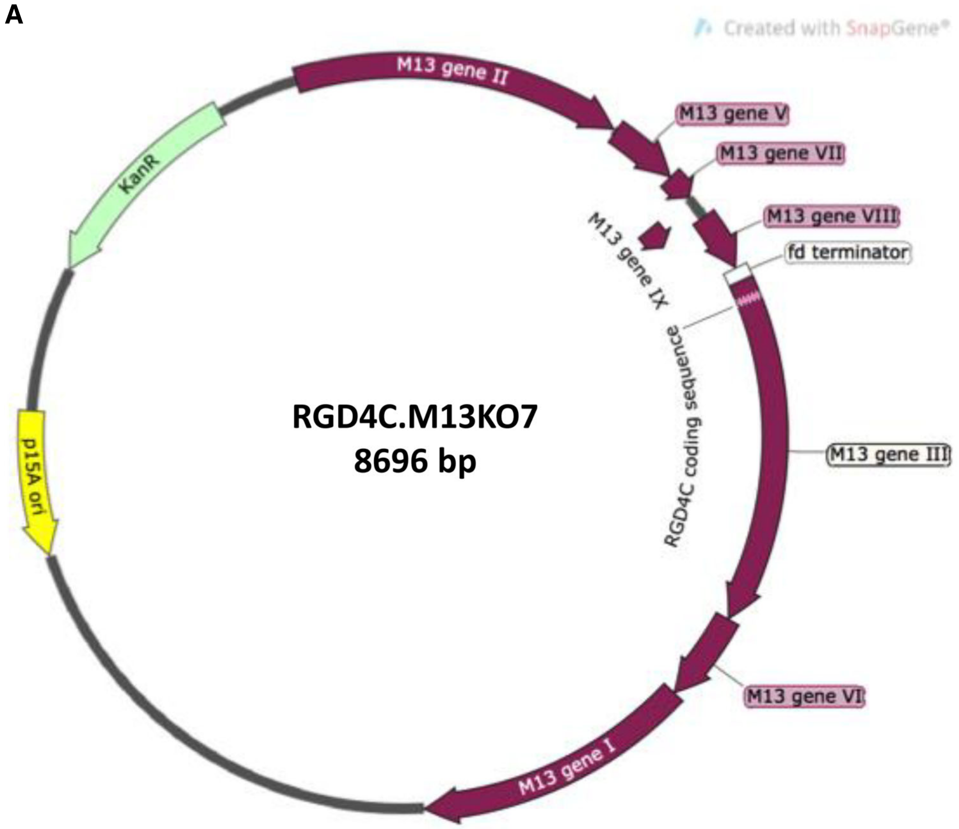
A
Created with SnapGene®
M13 gene II
M13 gene V
M13 gene VII
M13 gene VIII
fd terminator
M13 gene IX
RGD4C coding sequence
M13 gene III
M13 gene VI
M13 gene I
KanR
p15A ori
RGD4C.M13KO7
8696 bp

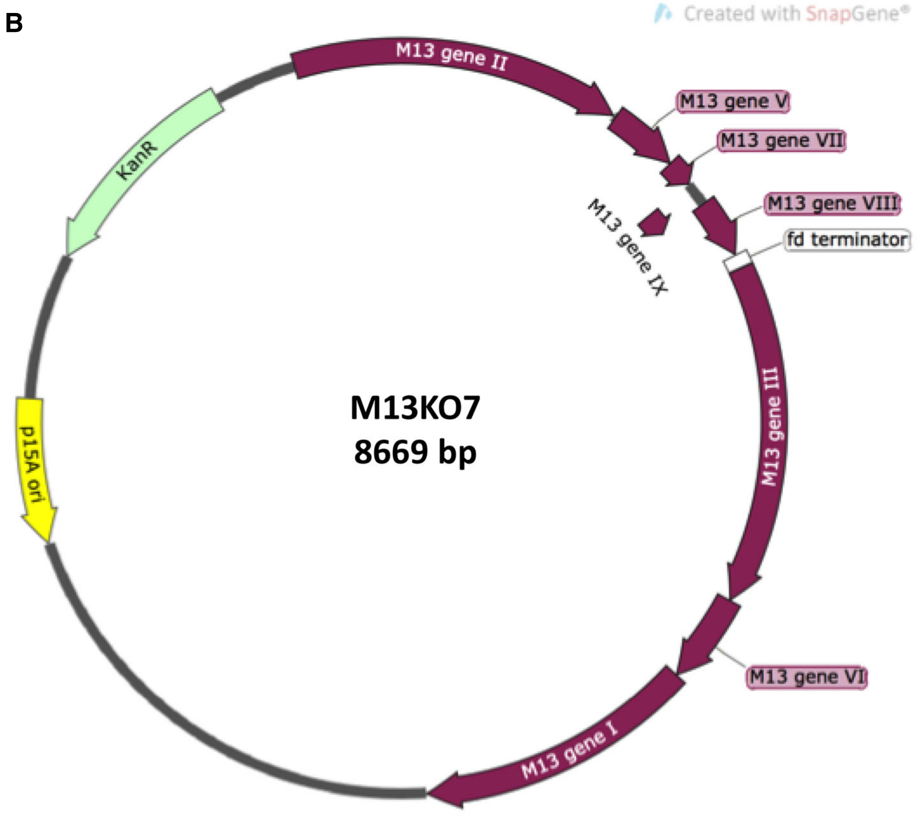
B
Created with SnapGene®
M13 gene II
M13 gene V
M13 gene VII
M13 gene VIII
fd terminator
M13 gene IX
M13 gene III
M13 gene VI
M13 gene I
KanR
p15A ori
M13KO7
8669 bp

Figure EV2.

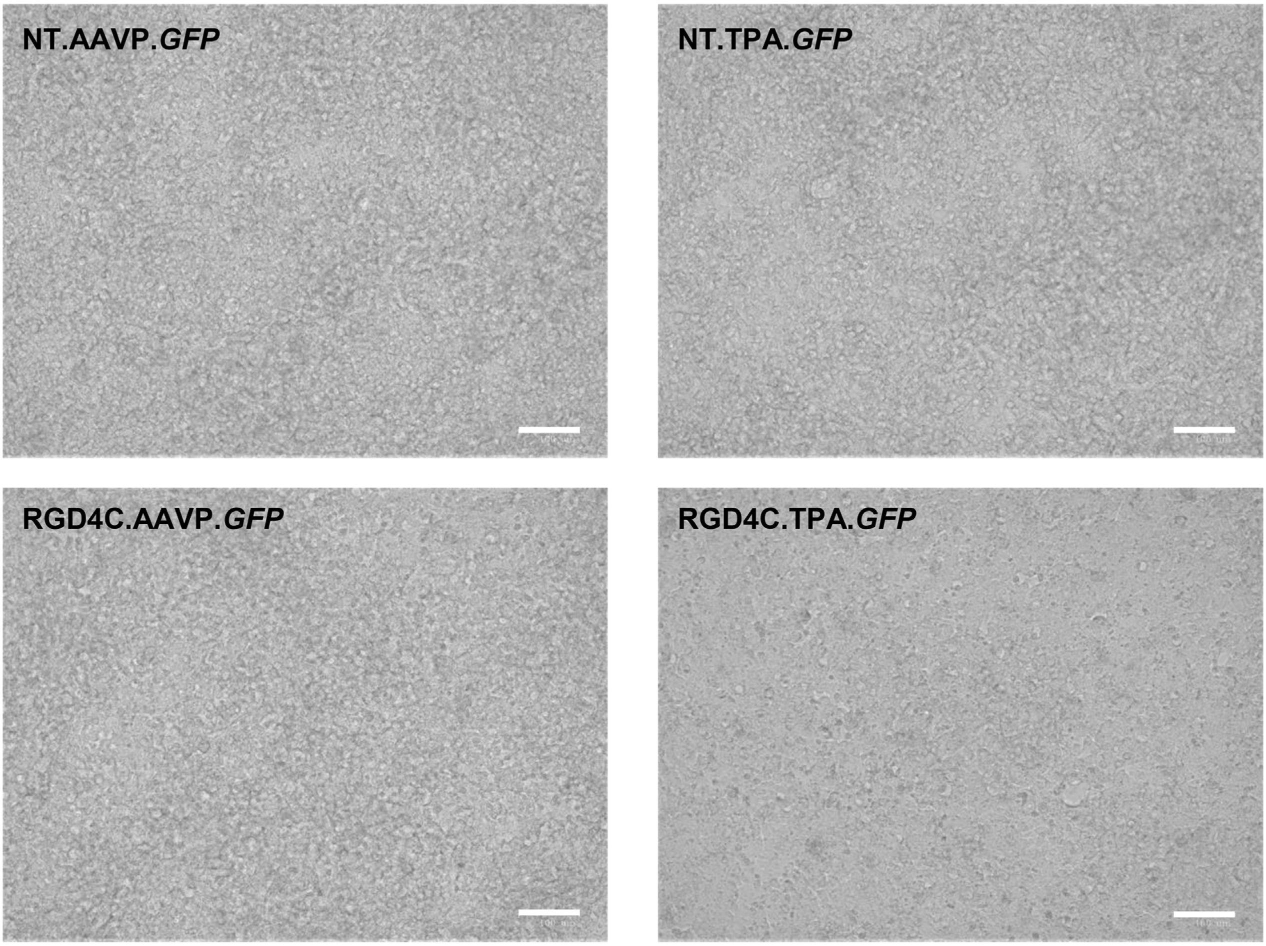

Figure EV3. Phase contract images of HEK293 cells treated with TPA.*GFP* and AAVP.*GFP*.

Images of cells were obtained at day 7 post-treatment with 1×10^6 TU/cell of either RGD4C.TPA.*GFP* or RGD4C.AAVP.*GFP*. Non-targeted (NT) vectors were included as controls. Data shown are representative of three independent experiments ($n = 3$). Scale bar, 100 μm.

Source data are available online for this figure.

Figure EV4. TPA particle biodistribution and targeted cytokine therapy.

A Cohorts of immunocompetent BALB/c mice with established subcutaneous tumours derived from CT26.CL25 cells ($n = 6$ mice), were systemically administered with increasing doses 5×10^9, 1×10^{10} and 1×10^{11} TU/mouse, of targeted (RGD4C) or non-targeted (NT) TPA.$IL15^{IgK}$. $IL15^{IgK}$ gene expression was assessed by quantification of the $IL15^{IgK}$ mRNA in tumours and healthy tissues after 5 days. Data shown are representative of one experiment, $n = 2$ technical replicates.

B Graphs showing tumour growth after targeted delivery of $IL15^{IgK}$ or *IL12*. Tumour-bearing mice injected with non-targeted (NT) TPA particles were included as controls. Data from Figs 6D and 7C were used to show the mean of total tumour luminescence from day 0 to day 5 ± SEM ($n = 4$ mice per group).

Source data are available online for this figure.

 © 2022 The Authors

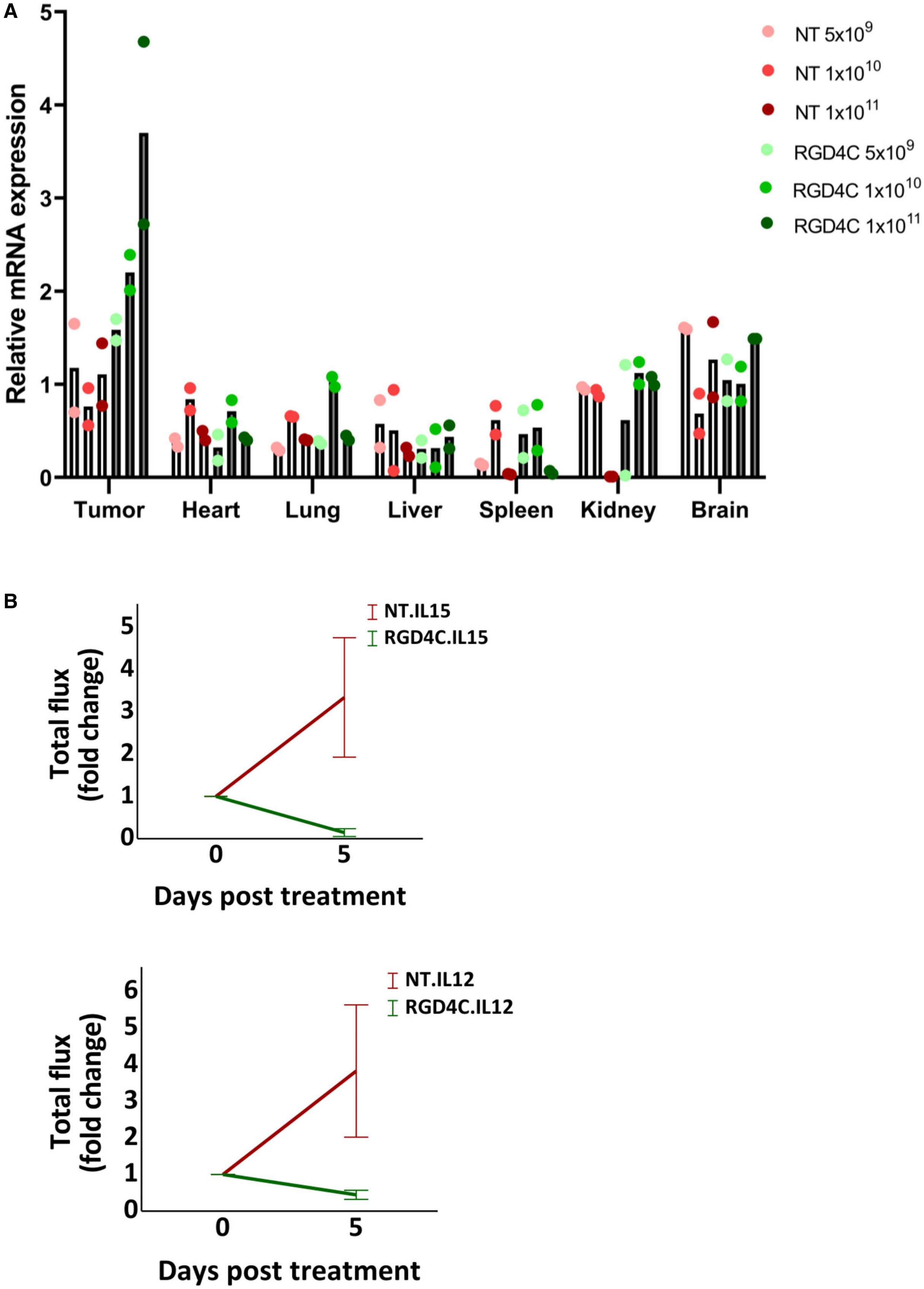
A
Relative mRNA expression
0
1
2
3
4
5
Tumor
Heart
Lung
Liver
Spleen
Kidney
Brain
NT 5x10^9
NT 1x10^10
NT 1x10^11
RGD4C 5x10^9
RGD4C 1x10^10
RGD4C 1x10^11
B
Total flux (fold change)
Days post treatment
NT.IL15
RGD4C.IL15
NT.IL12
RGD4C.IL12

Figure EV4.

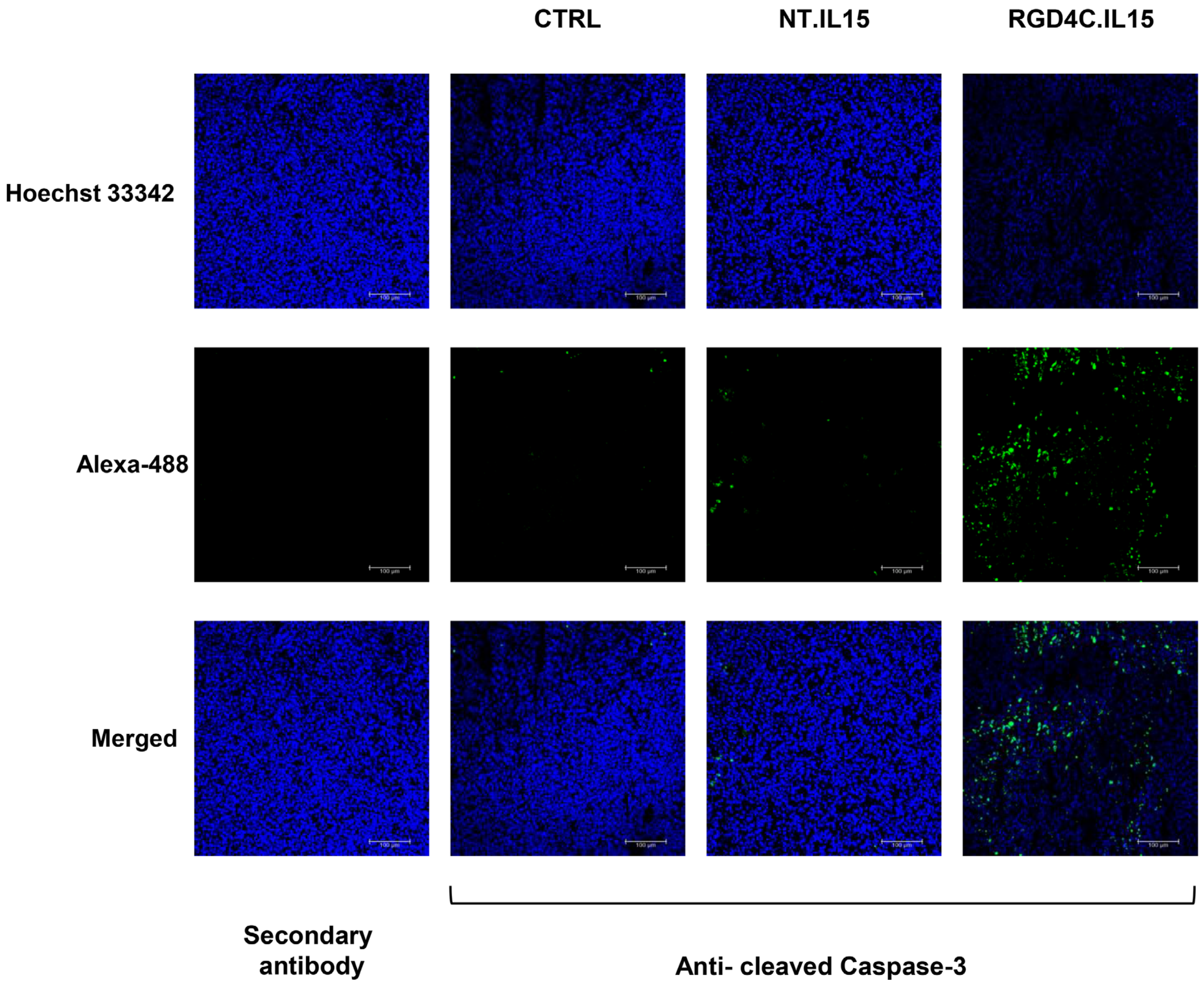

Figure EV5. Immunostaining of tumour sections using an anti-cleaved caspase-3 antibody.

Tumour sections from tumour-bearing mice ($n = 6$) following single TPA.*IL15*^{IgK} dose treatment, 5×10^{10} TU/mouse, of targeted (RGD4C.*IL15*) or non-targeted (NT.*IL15*). Untreated mice were used as controls (CTRL). Tumour sections incubated with the secondary antibody alone were also included as negative controls. Hoechst 33342 was used to stain the cell nuclei. Scale bar, 100 μm.

Source data are available online for this figure.

© 2022 The Authors
