# Supplementary material for: Systemically targeted cancer immunotherapy and gene delivery using transmorphic particles
Source: EMBO Mol Med. 2022 Jun 27;14(8):e15418. doi: 10.15252/emmm.202115418 (PMC9358398; doi:10.15252/emmm.202115418)
Supplement: Supplementary file 2 — Table EV1 [file EMMM-14-e15418-s010.docx]

**Table EV1. List of oligonucleotide sequences used for molecular cloning.**

| **Name** | **Sequences (5’-3’)** |
| --- | --- |
| IL2 sense | ATCGAGGATCCATGTACAGAATGCAACTCCTGTCTTGTATTGCACTAAGTCTCGCACTTGTCACAAACAGTGAATTCATCGA |
| IL2 antisense | TCGATGAATTCACTGTTTGTGACAAGTGCGAGACTTAGTGCAATACAAGACAGGAGTTGCATTCTGTACATGGATCCTCGAT |
| IgK sense | ATCGAGGATCCATGGAGACAGACACACTCCTGCTATGGGTACTGCTGCTCTGGGTTCCAGGTTCCACTGGTGACGAATTCATCGA |
| IgK antisense | TCGATGAATTCGTCACCAGTGGAACCTGGAACCCAGAGCAGCAGTACCCATAGCAGGAGTGTGTCTGTCTCCATGGATCCTCGAT |
| h TNF$a$ Fw | ATCGGAATTCGTCAGATCATCTTCTCGAACCCCGA |
| h TNF$a$ Rev | CGCTAGTCGACGTCTGGCCAGCTAGCTCACAG |
| m IL15 Fw | ATCGGAATTCGGCATTCATGTCTTCATTTTGGG |
| m IL15 Rev | ATCGGTCGACTGCAGTCAGGACGTGTTGATGAA |
| m IL12 Fw | GTAGGCGCGCCATGTGTCAATCACGCTACC |
| m IL12 Rev | GTAGTCGACCTAGGATCGGACCCTG |
| M13.RGD Fw | AGGGGGGATTGTTTTTGTGAAACTGTTGAAAGTTGTTTAGCAAAACCCC |
| M13.RGD Rev | ACAATCACAAGCGGAGTGAGAATAGAAAGGAACAAC |
| Lucia Fw | CGCTACGCTAGAATTCGTCACCATGGAAATCAAGGTGCTG |
| Lucia Rev | CGCTACGCTAGGATCCGCTATCATCTGTCCCCAGCCAGA |

h: human

m: mouse
