# Supplementary material for: Systemically targeted cancer immunotherapy and gene delivery using transmorphic particles
Source: EMBO Mol Med. 2022 Jun 27;14(8):e15418. doi: 10.15252/emmm.202115418 (PMC9358398; doi:10.15252/emmm.202115418)
Supplement: Supplementary file 3 — Table EV2 [file EMMM-14-e15418-s003.docx]

**Table EV2. List of oligonucleotide sequences used for qPCR.**

| **Name** | **Sequences (5’-3’)** |
| --- | --- |
| ITR Fw | GGAACCCCTAGTGATGGAGTT |
| ITR Rev | CGGCCTCAGTGAGCGA |
| h TNF$a$ Fw | GCCCATGTTGTAGCAAACCC |
| h TNF$a$ Rev | TATCTCTCAGCTCCACGCCA |
| m IL15 Fw | GTGACTTTCATCCCAGTTGC |
| m IL15 Rev | TTCCTTGCAGCCAGATTCTG |
| m GAPDH Fw | AACTTTGGCATTGTGGAAGG |
| m GAPDH Rev | ACACATTGGGGGTAGGAACA |
| m CD8a Fw | ACTACCAAGCCAGTGCTGCGAA |
| m CD8a Rev | ATCACAGGCGAAGTCCAATCCG |
| m CD8b Fw | GAATGTGAAGCCAGAGGACAGTG |
| m CD8b Rev | GGGCAGTTGTAGGAAGGACATC |
| m NKp46 Fw | TAGGGCTCACAGAGGGACATAC |
| m NKp46 Rev | GTAGGTGCAAGGCTGCTGTTCT |
| m GZMa Fw | GTGGTGGAAAGGACTCCTGCAA |
| m GZMa Rev | GAGAGGAAAGTATAGACACCAGG |
| m GZMb Fw | CAGGAGAAGACCCAGCAAGTCA |
| m GZMb Rev | CTCACAGCTCTAGTCCTCTTGG |
| m Prf1 Fw | ACACAGTAGAGTGTCGCATGTAC |
| m Prf1 Rev | GTGGAGCTGTTAAAGTTGCGGG |
| m TBX21 Fw | CCACCTGTTGTGGTCCAAGTTC |
| m TBX21 Rev | CCACAAACATCCTGTAATGGCTTG |
| m FoxP3 Fw | CCTGGTTGTGAGAAGGTCTTCG |
| m FoxP3 Rev | TGCTCCAGAGACTGCACCACTT |
