# Supplementary material for: Systemically targeted cancer immunotherapy and gene delivery using transmorphic particles
Source: EMBO Mol Med. 2022 Jun 27;14(8):e15418. doi: 10.15252/emmm.202115418 (PMC9358398; doi:10.15252/emmm.202115418)
Supplement: Supplementary file 11 [file EMMM-14-e15418-s007.pdf]

EMBO Molecular Medicine

Systemically Targeted Cancer Immunotherapy and Gene Delivery using Transmorphic Particles.

Paladd Asavarut, Sajee Waramit, Keittisak Suwan, Gert Marais, Aitthiphon Chongchai, Surachet Benjathummarak, Mariam Al-Bahrani, Paula Vila-Gomez, Matthew Williams, Prachya Kongtawelert, Teerapong Yata, and Amin Hajitou
DOI: 10.15252/emmm.202115418

Corresponding author: Amin Hajitou (a.hajitou@imperial.ac.uk)

Review Timeline:

Submission Date:	30th Nov 21
Editorial Decision:	26th Jan 22
Revision Received:	11th May 22
Editorial Decision:	16th May 22
Revision Received:	23rd May 22
Accepted:	24th May 22

Editor: Lise Roth

Transaction Report:

(Note: Please note that the manuscript was previously reviewed at another journal and the author response to these referee reports were taken into account in the editorial decision at EMBO Molecular Medicine. With the exception of the correction of typographical or spelling errors that could be a source of ambiguity, letters and reports are not edited. Referee reports are anonymous unless the Referee chooses to sign their reports.)

Responses to reviewers' comments

Reviewer #1: Asavarut et al. describes an optimized viral vector particle that chimerizes an AAV genome with a bacteriophage capsid (transmorphic phage/aav = TPA). The phage capsid was previously shown to allow retargeting by insertion of RGD4C, a known high-affinity ligand of αvβ3 integrin which is highly expressed on tumors and newly sprouted blood vessels. The innovation in this resource paper is the progression of the previous generation of phage/AAV (AAVP) to the current one by avoiding helper phage genome to be co-packaged. This consequently has the possible benefit to ensure the phage particles to be smaller and more stable. The studies further characterize TPA particles, and demonstrate the potential in tumor killing in mouse models and in vitro.

The data demonstrates quite clearly TPA is a vector system of interest and likely an improvement compared to the previous generation of phage/aav vector systems. The ability to create such an hybrid system is remarkable; this group has persisted with these efforts over the year and should be complemented. The versatility of the system to overcome existing limitations of AAV, particularly genome size, seems to be an important attribute. Overall, the data is compelling in demonstrating the improvement, and the utility in vitro and mouse.

However, this reviewer highlights a number of limitations to the study, both conceptual, technical, and stylistically.

Conceptual

1. The authors, notwithstanding their remarkable synthetic biology feat, fail to articulate and demonstrate the key advantages of their system. It is not entirely clear to the reviewer why a RGD4C phage capsid would be preferred for tumor targeting over other RGD4C targeting approaches. The specificity argument is possibly a powerful one, however the data is limited in this manuscript and in these models to demonstrate truly the level of specificity/potency of the system at therapeutic doses (e.g. DNA, not just RNA biodistribution studies).

Response: the RGD4C (CDCRGDCFC) was identified as the highest tumor homing phage, with a prevalence of 80%, in *in vivo* screenings upon intravenous injection of a phage display peptide library to mice bearing human breast tumours(1). The RGD4C/phage exhibits high tumor targeting and often is the winner in *in vivo* phage screenings in tumor-bearing mice unless we co-administer with RGD4C inhibitors

to identify other phage-peptides(2). We and others have published a large body of work proving the tumor selectivity of RGD4C-phage vectors in mice, rats and pet dogs with no gene delivery detected in healthy tissues(3-5). Recently, we reported that a panel of normal human primary cells from different histological origins do not express or have very low expression of the $\alpha_v\beta_3$ and $\alpha_v\beta_5$ integrin receptors of RGD4C(6). Notably, this very low integrin profile did not translate into gene delivery to normal cells by RGD4C/phage vectors. Finally, we also confirmed the tumor selectivity of RGD4C.TPA that we report in this manuscript.

2. While some progress is reported on minimizing helper phase packaging, the authors still report 2% of particles to be phage. Presumably at very high doses that would be used clinically that level of contamination would be considered a remaining safety issue.

Response: in our study, we reported a contamination of phage in the TPA isolate of 2% or less. This contamination rate is low and increases the novelty of our study, as no phage-derived expression systems utilizing a helper bacteriophage has been able to achieve particle yields while maintaining a contamination rate lower than 10%(7). We understand the reviewer's concern and would like to highlight that in clinical applications, the TPA isolate can be purified to separate the phage contamination using routine methods in the industry, such as ultracentrifugation or fast-protein liquid chromatography, which is currently used to purify bacteriophage preparations in a commercial scale. We have addressed this concern through clarification in the main text.

3. The authors fail to compellingly provide their rationale for the design considerations of their aav/phage. Why phage? If one is able to make the AAV capsid inert for all tissues, but biodistributing only to tumors, would that achieve the same goal? Why AAV? Do you need the ITRs? What value do they bring? What is the translational relevance of the particular retargeting peptide used, beyond from mouse models? Is less restricted genome size important for the application that the authors pursue? If helper phage genome remains a fairly significant contaminant in either system (albeit reduced in TPA) is the advantage of having smaller particles important in TPA? Do you have stability data to demonstrate this within this system?

Response: in our study, we designed and characterised a novel particle, transmorphic phage/AAV (TPA), to address an unmet need in both AAV and bacteriophage as individual vectors due to their native biology. AAV are eukaryotic viruses, which fundamentally lack tissue specificity and are immunogenic, despite being highly efficient at gene expression conferred upon by their ITR sequences. Although it has

been shown that serotyping or pseudotyping AAV to distinguished tissue target groups does indeed improve specificity, AAV vectors still cannot safely and systemically target pathology in vivo. In short, the AAV capsid cannot be made selectively inert or highly specific to mammalian tissue due to its inherent biology. Moreover, peptides identified by the phage display technology have been used to generate ligand-targeted vectors to target the cells of interest. For viral vectors, the ideal approach is to genetically engineer new ligands into the capsid proteins of the virus to generate a single agent. Although this is ideal, this insertion of an exogenous ligand from one structural context into the differing structural context of a capsid protein can ablate the function of the ligand or disrupt viral assembly and function. These "context" problems are fundamental, since an ideal candidate peptide ligand may be identified, by phage display screenings, but cannot be applied because the ligand destroys the vector or the vector destroys the ligand. This translation problem stems in part from the fact that peptides isolated from phage libraries are selected in the protein structural context of the pIII capsid protein of the M13 phage and are then translated into different protein structures of a viral capsid protein(8). Therefore, we packaged the efficient rAAV transgene cassette (containing ITRs) using the capsid of bacteriophage because of the advantages given by their coat proteins. Specifically, the phage capsid can be modified to be highly specific to tissue targets through insertion mutations (in our case, RGD4C), and are less immunogenic than mammalian viruses as their natural host are prokaryotes; we also reported a large body of literature to show that their repeated administrations, to achieve a therapeutic response, are not a problem. Another important aspect is that bacteriophage-based expression systems will enable industrial-scale production at a fraction of the cost compared to mammalian viruses. Furthermore, phage vectors have previously been shown to effectively treat soft tissue sarcomas in pet dogs to the point of cure in several subjects, as mentioned in our references. Finally, regarding the role of ITRs, we previously reported that ITR-flanked transgene cassettes provide better gene expression by phage vectors compared to conventional transgenes cassettes, lacking the ITRs, through maintenance of the entire mammalian transgene cassette, better persistence of episomal DNA and formation of concatemers of the transgene cassette(2).

The reviewer brings up an important point concerning particle size. We believe that particle size significantly impacts the efficacy of TPA particles, and that is precisely what we have demonstrated in our study. The particles described is a rAAV genome packaged using bacteriophage coat proteins, which is a key point of novelty as it is truly transmorphic. To be precise, the TPA genome does not contain any phage structural genes, thus differentiating TPA from other hybrid or chimeric phage

vectors in existence. The significant reduction in genome size that follows positively impacts its efficacy in gene transduction and cancer immunotherapy, as shown in our in vitro and preclinical data. We investigated the impact of size through our *in vitro* ECM diffusion assay and Matrigel transwell test, then in eukaryotic cells through a particle internalisation assay 2 and 4 hours post transduction and TPA genome nuclear accumulation. We would like to note that the reduction in genome size also introduces the possibility of packaging larger rAAV transgenes than previously reported in the literature. Because the length of rAAV transgenes is limited by the AAV capsid architecture, using a bacteriophage capsid, which extrudes depending on genome length, will enable transduction of longer or more complex AAV transgenes.

For long term stability, we monitored the TPA titer over 2 years at 4°C and found that the particles remain fairly stable.

Technical

4. The titration for phage is by transducing units (measured on bacteria). Is it possible that the RGD4C mutation alters the transduction biology or physical packaging? It would be important to measure also physical particles (e.g. genome copies), and compare these titers with AAVP and regular AAV production as a benchmark, and perform equal physical titer comparisons in all characterization studies.

Response: as determined by previous studies from our group, we have found no evidence to suggest that the RGD4C mutation is detrimental to replication and encapsulation of TPA particles or bacteriophage vectors. The pIII minor coat protein gene of the bacteriophage is robust and able to tolerate the 9 amino acid mutation on the particular cloning site. In fact, the ability of bacteriophages to tolerate mutations on its pIII gene is hallmark to the development of phage display as a field (awarded the Nobel Prize 2018 in Chemistry). In particular, phage libraries are able to withstand displaying even light chain antibody fragments, demonstrating their ability to tolerate mutations. The question of whether such mutations will affect packaging is a valid one; however we observed high-titre production of TPA particles and do not see that the RGD4C mutation has negatively impacted particle production. The yields achieved by our production protocol also surpasses those in the literature, including studies on AAVP, indicating novelty and efficiency of the TPA particle expression system.

The reviewer's comments on using genome copies as a measure is a valid criticism that we can perform and benefit from. However, we would like to mention that the infectivity of eukaryotic viruses (i.e. rAAV) cannot be directly compared to prokaryotic viruses or its derivative, as they belong to different native hosts and thus rely on different biological mechanisms of nucleic acid delivery to the nucleus.

Another important consideration is economic cost, as the trade-off in infectivity of the TPA (compared to mammalian viruses) may potentially be offset by the cost advantages of using a prokaryotes for its production rather an eukaryotic host cells.

5. *Specificity/biodistribution, arguably the defining feature of the innovation in the studies, needs to be demonstrated with more exhaustive molecular analyses and ideally in more relevant pharmacological in vivo models (e.g. NHP?)*

Response: we are in agreement with the reviewer, however, for the purposes of demonstrating clinical efficacy, we focused on simple but robust methods that demonstrate and confirm straightforward alterations in biodistribution and tumor selectivity. We feel that our experiments provide sufficient mechanistic insight to account for our observations in gene transduction selectivity and immunotherapy in vivo, however, we welcome suggestions on further molecular analyses.

Stylistically

- Two key concepts to the novelty and criticality in the manuscript are barely discussed; technically how is the TPA accomplished; is it really just splitting up the genes in 2 separate plasmids? Also, the targeting and selectivity of RGD4C is assumed. While I understand there is a body of literature on this approach, it is key to the authors argument that this targeting allows for pristine selectivity to be translated to ultimate clinical application. How sure can we be of this?

- Improved articulation of rationale

Response: we apologise for the stylistic improvements that the reviewer felt was needed. We have edited the main text to be clear on the design and construction of TPA particles. We would like to point out that the selectivity of the RGD4C is not assumed, but was investigated in the present study and also previously proven in numerous studies from multiple groups that demonstrated that it is an efficacious ligand for binding specifically to tumor cells in vitro and in vivo.

Reviewer #2: "Systemically Targeted Cancer Immunotherapy and Gene Delivery using Transmorphic Particles" is a manuscript last-authored by Dr. Amin Hajitou, who is an expert in experimental anti-cancer gene therapy delivery. In this study by Asavarut et al., a novel hybrid vector system based on a bacteriophage (phage) capsid and the DNA of recombinant human adeno- associated virus (AAV) using a tumor-targeted prokaryotic viral capsid has been designed and tested ex vivo and in vivo. A similar

vector, termed adeno-associated virus/phage (AAVP), had been previously reported by this group and enabled targeted transgene delivery(2). The AAVP contains the full phage genomic sequence that does not have a therapeutic value and has to be packaged into large particles. Here, the group designed a new system termed Transmorphic Phage/AAV (TPA). It is based on a helper phage which enables packaging of only the essential DNA material in a smaller particle that the authors hypothesized to have a better biodistribution profile. The RGD4C peptide that enables tumor targeting was used as in previous studies for comparison. A hybrid TNFα fused with the IL2 signal peptide and IL15 fused with IgK signal peptide were used as new experimental anti-cancer agents, along with IL12.

This proof of principle study is a significant contribution to the field of targeted cancer therapy. It introduces the new phage-AAV vector as a selective and efficient gene delivery tool. Low TPA particle production, and helper phage contamination were the technical obstacles that the authors encountered and have partly resolved. The use of three different cytokines in various cancer cell models reinforces the conclusions. While the study is important and may have a strong impact on the field, there are a number of technical and conceptual issues that need to be addressed.

Major points:

1. Individual phage filaments in Fig 2C are difficult to tell apart in images provided. It would be better to have filaments at lower density for analysis. Also, the TPA filaments appear to be thicker in Fig 2C: is this the case? If so, is this expected to affect tissue penetration?

Response: we note that performing the micrograph at lower density will yield a better image. The TPA filaments are not thicker than AAVP, we believe that this is an effect of contrast from the microscope. We quantified the thickness by measurement during analysis and did not detect any significant differences.

2. Is it possible that the particle titration method underestimates TPA density relative to AAVP due to worse bacterial infectivity of TPA (which yet might have high cell transducibility)? It would help to confirm titer equality by an alternative method, such as qPCR or immunologically with anti-phage antibodies.

Response: we agree with the reviewer that the study can benefit from using an alternate quantification method for TPA, especially qPCR to determine gene copies. We would like to point out that because the AAVP and TPA both contain pIII minor coat proteins that bear the RGD4C mutation, their bacterial

infectivity should be the same as the pIII is the site used by the bacteriophages to bind to the prokaryotic host for entry. If it does indeed affect entry, then it is intrinsically controlled as both AAVP and TPA carries the same mutation on the same protein gene.

3. For this initial TPA characterization, a side-by-side biodistribution study and evaluation of TPA vs AAVP targeting is essential in a mouse tumor model. Comparative immunohistochemistry with anti-phage antibodies needs to be performed. GFP or Luc tumor delivery with TPA vs AAVP also needs to be compared.

Response: the main objective of the study was to develop a novel phage vector for application in cancer immunotherapy. We included AAVP in the initial experiments as control for size to show that the reduced size of M13 phage-derived vectors does matter for gene delivery to mammalian cells as this is a growing hypothesis in the field.

We understand the reviewer's point, and that's why we report here the superiority of TPA to AAVP in various in vitro experiments. To comply with the reviewer, we can also carry out in vivo comparison in tumor-bearing mice to confirm the superiority of TPA to AAVP, for gene delivery to tumors, by using particles carrying either *Luc*, *TNF*α, *IL15* or *IL12* transgenes.

4. In vivo cytokine gene delivery data, starting with Fig 4, lacks AAVP comparison. Without it, it is impossible to evaluate the relative improvement of TPA-directed gene therapy. This would be particularly important because the TNFa and IL15 used here are modified and have not been tested in vivo before.

Response: we have generated data in vitro to show that RGD4C.TPA particles provide better delivery of our newly designed TNFα compared to RGD4C.AAVP. As mentioned in the above point-3 raised by this reviewer, we also can carry in vivo studies to analyse delivery of TNFα to tumors in preclinical models upon systemic administration.

5. N= of mice used in tumor treatment experiments cannot be found. They need to be provided for each efficacy experiment separately and statistical analysis needs to be revisited. Images of individual resected tumors should be provided at least in the Supplement. Tumor growth data are shown only for IL12. Please also provide them for TNFa and IL15.

Response: in the previous manuscript we provided the number of mice for in vivo experiments in the Methods section. We have now complied with the reviewer and provided this number for each

experiment. As requested by the reviewer, we will be happy to provide images of resected tumors and tumor growth data.

6. Presumably, at some load AAVP and TPA will transduce cells in vivo outside the tumor, possibly irrespective of RGD4C. It would be important to identify the number of TPA particles (with and without RGD4C) at which it happens, and what the normal organs targeted are, to anticipate possible side effects.

Response: the tumor selectivity of RGD4C-phages was raised above in point-1 of reviewe#1, please see our response. It is known that the phages are cleared by the reticulo-endothelial system, therefore we expect to see accumulation of both AAVP and TPA particles in phagocytic cells e.g. Kupfer cells the liver. However, the nonspecific hepatic clearance of phage particles (Geier et al., Nature 1973), does not result in an undesirable gene transduction of the liver. These observations are in sharp contrast with the well-documented nonspecific transduction of normal organs (such as liver) by the mammalian viral gene delivery vectors.

Minor points:

- The Figures are low resolution and it is hard to see some labels and evaluate certain data.

Response: we have improved the quality of the figures.

- Figure 2F: Brightfield cell images or nuclear staining would help to evaluate these data. The images aimed to show that RGD4C.TPA.GFP transduction is distinctly higher than that of RGD4C.AAVP.GFP have no signal at all for RGD4C.AAVP.GFP. That is unexpected. Is the frequency of GFP+ cells lower or the GFP signal / cell? By how much? Both need to be quantified. That would help relate these data to Fig 3D quantification where difference is only 2-fold.

Response: we can provide brightfield cell images. The reviewer's observation that GFP expression by RGD4C.AAVP.GFP was too low is an important point. Indeed, the transduction efficiency of RGD4C.AAVP particle can be highly variable for the same cell type and reach sometime low levels around 0.3%. We still don't know the factors behind this, but the TPA always performed better than AAVP for gene delivery, in all experiments where the two particles were compared side-by-side.

- While the prediction that smaller particles should have better tissue permeability are reasonable and confirmed with the Matrigel experiments (Fig 3), it is not clear why TPA transduces so much better in cell

culture experiments (Fig 2F) were there is no ECM hinderance: is there an explanation? Could it be due to some TPA phage not accounted for by bacterial titration?

Response: as we stated in the manuscript improved diffusion through the ECM is one mechanism through which TPA can transduce cells better than AAVP. However, we also report that TAP particles have better cell entry and better nuclear accumulation which are important steps in gene delivery efficacy.

- Fig 3E: why did AAVP remain predominantly in the cytoplasm? What explains better nuclear localization of TPA DNA?

Response: important point. We believe that the TPA contains and delivers the AAV genome only which has evolved optimised mechanisms for intracellular trafficking in mammalian cells. While AAVP delivers both AAV but also a large bacteriophage genome that has no optimised strategies for intracellular trafficking in mammalian cells.

- AAV DNA integrates into the genome. This needs to be discussed. Have the authors analyzed integration of AAVP vs TPA DNA? Has it been analyzed how long the transgenes are expressed for post-injection in vivo?

Response: we previously investigated integration of AAVP DNA (Hajitou et al. cell 2006) and found that there is no integration, and that the AAVP genome remains extrachromosomal. Regarding the long-term transgene expression in tumors in vivo, this can vary depending on the tumor type and how fast the tumors become necrotic(2, 3). We will be happy to include a discussion on this in the manuscript.

- It is mentioned once that RGD4C, the peptide used for phage tumor delivery, binds to αv integrins. Because αv integrins are also expressed outside tumors, some more discussion of the results and future perspectives are warranted.

Response: the tumor selectivity of RGD4C peptide was also raised in point-1 of reviewer#1 (please see our response).

The RGD4C ligand binds mainly to $\alpha_v\beta_3$, but also to a lesser level to the $\alpha_v\beta_5$ heterodimer. Various integrin heterodimers can be found in wide variety of human cells; however, both $\alpha_v\beta_3$ and $\alpha_v\beta_5$ are highly restricted and typically overexpressed on cancer cells and tumor vasculature(1, 9). In Human biopsies, the $\alpha_v\beta_3$ integrin is widely expressed on blood vessels of human tumor biopsy samples but not on vessels of biopsies from normal tissues; the distribution of $\alpha_v\beta_3$ in human is highly restricted, with

expression on activated endothelium, activated vascular smooth muscle and tumors. Besides this, the $\alpha_v\beta_3$ has been shown to have relatively limited cellular distribution in humans quiescent tissues; apart from its expression at high levels in the inflamed synovial tissues of rheumatoid arthritis patients (10, 11), $\alpha_v\beta_3$ is absent or minimally, or barely detectable on endothelial cells(12), some B-cells, platelets, monocytes, intestinal cells, and smooth muscle cells, as well as a small percentage of activated leukocytes, macrophages, and osteoclasts. Integrin $\alpha_v\beta_5$ is often found in the same pathological contexts as $\alpha_v\beta_3$, but can also be found in fibroblasts. Importantly, we previously reported that there is no expression or minimal expression of these integrins in human normal cells, but this low level of αv integrins does not permit any gene expression by RGD4C.phage vectors(6).

- In mouse experiments, do primers used for RT-qPCR detect only the delivered cytokine gene or also the endogenous cytokine?

Response: no cytokine mRNA signal was detected in control untreated cells.

- The group recently used a phage-AAV vector for experimental drug delivery of TNFα (Chongchai A, et al. FASEB J. 2021). In that study, an endosomal escape peptide, H5W, was displayed on pVIII coat protein to enhance gene delivery. It is not clear why the authors chose to not do it for TPA and discussion along these lines would be welcome.

Response: we have data showing that display of the endosomal escape peptide, H5W, on a major pVIII coat protein of the TPA particles further enhances gene delivery efficacy. We will be happy to include these data.

- Results contain a lot of technical details on vector construction, characterization and optimization that appear to be better suited for Methods and/or Supplemental Materials. Also, some parts of Results, such as cancer type descriptions and cytokine MOA seem to belong better in Introduction or Discussion.
Text needs to be proof-read. There are typos and language imperfections. It is advised that the manuscript is edited for clarity. For example, the meaning of the sentence "Furthermore, 90% of patients die from metastatic cancer, which relies on the systemic delivery (6)." Is not clear. Reference call out format is not internally consistent.

Response: we have complied and revised the text to improve the manuscript and will continue to do so.

- TNFα has both anti and pro-cancer effects. For example, it has been reported that TNFα interferes with

immune checkpoint blockade approaches to cancer treatment (e.g. Bertrand et al 2017) and can increase cancer cell aggressiveness (W. Liu et al Scientific Reports 2020). This, along with issues related to potential adverse effects of IL15 and IL12, need to be discussed.

Response: yes, it is correct that TNFα can have both anti and pro-cancer effects but this depends on the levels of its release and production within the tumors and whether its production is selectively localised or not. We and other groups have reported that selective delivery of TNFα by RGD4C.phages results in anti-tumor effects in rodents and large animals e.g., pet dogs with natural cancers(4, 13-17).

REFERENCES

1. Arap W, Kolonin MG, Trepel M, Lahdenranta J, Cardo-Vila M, Giordano RJ, et al. Steps toward mapping the human vasculature by phage display. Nat Med. 2002;8(2):121-7.
2. Hajitou A, Trepel M, Lilley CE, Soghomonyan S, Alauddin MM, Marini FC, 3rd, et al. A hybrid vector for ligand-directed tumor targeting and molecular imaging. Cell. 2006;125(2):385-98.
3. Hajitou A, Lev DC, Hannay JA, Korchin B, Staquicini FI, Soghomonyan S, et al. A preclinical model for predicting drug response in soft-tissue sarcoma with targeted AAVP molecular imaging. Proceedings of the National Academy of Sciences of the United States of America. 2008;105(11):4471-6.
4. Paoloni MC, Tandle A, Mazcko C, Hanna E, Kachala S, Leblanc A, et al. Launching a novel preclinical infrastructure: comparative oncology trials consortium directed therapeutic targeting of TNFalpha to cancer vasculature. PloS one. 2009;4(3):e4972.
5. Przystal JM, Hajji N, Khozoie C, Renziehausen A, Zeng Q, Abaitua F, et al. Efficacy of arginine depletion by ADI-PEG20 in an intracranial model of GBM. Cell Death Dis. 2018;9(12):1192.
6. Przystal JM, Waramit S, Pranjol MZI, Yan W, Chu G, Chongchai A, et al. Efficacy of systemic temozolomide-activated phage-targeted gene therapy in human glioblastoma. EMBO Mol Med. 2019;11(4).
7. Chasteen L, Ayriss J, Pavlik P, Bradbury AR. Eliminating helper phage from phage display. Nucleic acids research. 2006;34(21):e145.
8. Ghosh D, Barry MA. Selection of muscle-binding peptides from context-specific peptide-presenting phage libraries for adenoviral vector targeting. J Virol. 2005;79(21):13667-72.
9. Hemminki A, Belousova N, Zinn KR, Liu B, Wang M, Chaudhuri TR, et al. An adenovirus with enhanced infectivity mediates molecular chemotherapy of ovarian cancer cells and allows imaging of gene expression. Mol Ther. 2001;4(3):223-31.
10. Gamble LJ, Borovjagin AV, Matthews QL. Role of RGD-containing ligands in targeting cellular integrins: Applications for ovarian cancer virotherapy (Review). Exp Ther Med. 2010;1(2):233-40.
11. Koch AE. The role of angiogenesis in rheumatoid arthritis: recent developments. Ann Rheum Dis. 2000;59 Suppl 1:i65-71.

12. Van De Wiele C, Oltenfreiter R, De Winter O, Signore A, Slegers G, Dierckx RA. Tumour angiogenesis pathways: related clinical issues and implications for nuclear medicine imaging. Eur J Nucl Med Mol Imaging. 2002;29(5):699-709.
13. Tandle A, Hanna E, Lorang D, Hajitou A, Moya CA, Pasqualini R, et al. Tumor vasculature-targeted delivery of tumor necrosis factor-alpha. Cancer. 2009;115(1):128-39.
14. Smith TL, Yuan Z, Cardo-Vila M, Sanchez Claros C, Adem A, Cui MH, et al. AAVP displaying octreotide for ligand-directed therapeutic transgene delivery in neuroendocrine tumors of the pancreas. Proc Natl Acad Sci U S A. 2016;113(9):2466-71.
15. Staquicini FI, Smith TL, Tang FHF, Gelovani JG, Giordano RJ, Libutti SK, et al. Targeted AAVP-based therapy in a mouse model of human glioblastoma: a comparison of cytotoxic versus suicide gene delivery strategies. Cancer gene therapy. 2020;27(5):301-10.
16. Yuan Z, Syrkin G, Adem A, Geha R, Pastoriza J, Vrikshajanani C, et al. Blockade of inhibitors of apoptosis (IAPs) in combination with tumor-targeted delivery of tumor necrosis factor-alpha leads to synergistic antitumor activity. Cancer gene therapy. 2013;20(1):46-56.
17. Chongchai A, Waramit S, Suwan K, Al-Bahrani M, Udomruk S, Phitak T, et al. Bacteriophage-mediated therapy of chondrosarcoma by selective delivery of the tumor necrosis factor alpha (TNFalpha) gene. FASEB J. 2021;35(5):e21487.

26th Jan 2022

Dear Amin,

Thank you for submitting your work to EMBO Molecular Medicine, and please accept my apologies for the delay in getting back to you. I had originally secured an advisor who promised a report by the end of 2021, but never got back to us despite several chasers. I therefore contacted a second advisor, who has now provided a feedback.
This advisor stated:

"I have now had a look at the manuscript by Asavurat et al, the reviewers comments and the response to these. I think overall the data is convincing, well presented and supports the conclusions drawn. In particular, the in vivo efficacy of the TPA approach across different tumour models in both immunocompetent and immunosuppressed mice is impressive.
In terms of the initial reviewers' comments, which are very thorough and overall positive, in my view these have been thoughtfully and sufficiently addressed.
The major point to raise concerning the rebuttal is that, in response to reviewer 2, points 3 and 4, the authors respond that they can compare TPA and AAVP for gene delivery in vivo, although they have not actually done this. However, I think this is acceptable, since they have clearly demonstrated this superiority in vitro. I personally would not request further in vivo experiments for this manuscript.
Hence, in summary, I think this is a high-quality paper, for which the initial reviewers' concerns have been sufficiently addressed. I would therefore be happy to now recommend it to be accepted for publication."

Based on this advisor's input and after discussion within the team, I am pleased to inform you that we will be able to accept your manuscript, once editorial revisions will be performed.
We require:

1) A .docx formatted version of the manuscript text (including legends for main figures, EV figures and tables). Please make sure that the changes are highlighted to be clearly visible.

2) Individual production quality figure files as .eps, .tif, .jpg (one file per figure). For guidance, download the 'Figure Guide PDF' (https://www.embopress.org/page/journal/17574684/authorguide#figureformat).

3) A .docx formatted letter INCLUDING the reviewers' reports and your detailed point-by-point responses to their comments. As part of the EMBO Press transparent editorial process, the point-by-point response is part of the Review Process File (RPF), which will be published alongside your paper.

4) A complete author checklist, which you can download from our author guidelines (https://www.embopress.org/page/journal/17574684/authorguide#submissionofrevisions). Please insert information in the checklist that is also reflected in the manuscript. The completed author checklist will also be part of the RPF.

5) Please note that all corresponding authors are required to supply an ORCID ID for their name upon submission of a revised manuscript.

6) It is mandatory to include a 'Data Availability' section after the Materials and Methods. Before submitting your revision, primary datasets produced in this study need to be deposited in an appropriate public database, and the accession numbers and database listed under 'Data Availability'. Please remember to provide a reviewer password if the datasets are not yet public (see https://www.embopress.org/page/journal/17574684/authorguide#dataavailability).
In case you have no data that requires deposition in a public database, please state so in this section. Note that the Data Availability Section is restricted to new primary data that are part of this study.

7) For data quantification: please specify the name of the statistical test used to generate error bars and P values, the number (n) of independent experiments (specify technical or biological replicates) underlying each data point and the test used to calculate p-values in each figure legend. The figure legends should contain a basic description of n, P and the test applied. Graphs must include a description of the bars and the error bars (s.d., s.e.m.). Exact p values are required.

8) We would also encourage you to include the source data for figure panels that show essential data. Numerical data should be provided as individual .xls or .csv files (including a tab describing the data). For blots or microscopy, uncropped images should be submitted (using a zip archive if multiple images need to be supplied for one panel). Additional information on source data and instruction on how to label the files are available at
.

9) Our journal encourages inclusion of *data citations in the reference list* to directly cite datasets that were re-used and

obtained from public databases. Data citations in the article text are distinct from normal bibliographical citations and should directly link to the database records from which the data can be accessed. In the main text, data citations are formatted as follows: "Data ref: Smith et al, 2001" or "Data ref: NCBI Sequence Read Archive PRJNA342805, 2017". In the Reference list, data citations must be labeled with "[DATASET]". A data reference must provide the database name, accession number/identifiers and a resolvable link to the landing page from which the data can be accessed at the end of the reference. Further instructions are available at .

10) We replaced Supplementary Information with Expanded View (EV) Figures and Tables that are collapsible/expandable online. A maximum of 5 EV Figures can be typeset. EV Figures should be cited as 'Figure EV1, Figure EV2" etc... in the text and their respective legends should be included in the main text after the legends of regular figures.
- For the figures that you do NOT wish to display as Expanded View figures, they should be bundled together with their legends in a single PDF file called *Appendix*, which should start with a short Table of Content. Appendix figures should be referred to in the main text as: "Appendix Figure S1, Appendix Figure S2" etc.
- Additional Tables/Datasets should be labeled and referred to as Table EV1, Dataset EV1, etc. Legends have to be provided in a separate tab in case of .xls files. Alternatively, the legend can be supplied as a separate text file (README) and zipped together with the Table/Dataset file.
See detailed instructions here:

11) The paper explained: EMBO Molecular Medicine articles are accompanied by a summary of the articles to emphasize the major findings in the paper and their medical implications for the non-specialist reader. Please provide a draft summary of your article highlighting
- the medical issue you are addressing,
- the results obtained and
- their clinical impact.

This may be edited to ensure that readers understand the significance and context of the research. Please refer to any of our published articles for an example.

12) For more information: There is space at the end of each article to list relevant web links for further consultation by our readers. Could you identify some relevant ones and provide such information as well? Some examples are patient associations, relevant databases, OMIM/proteins/genes links, author's websites, etc...

13) Author contributions: the contribution of every author must be detailed in a separate section (before the acknowledgments).

14) Conflict of interest: We updated our journal's competing interests policy in January 2022 and request authors to consider both actual and perceived competing interests. Please review the policy https://www.embopress.org/competing-interests and update your competing interests if necessary.

15) Every published paper now includes a 'Synopsis' to further enhance discoverability. Synopses are displayed on the journal webpage and are freely accessible to all readers. They include a short stand first (maximum of 300 characters, including space) as well as 2-5 one-sentences bullet points that summarizes the paper. Please write the bullet points to summarize the key NEW findings. They should be designed to be complementary to the abstract - i.e. not repeat the same text. We encourage inclusion of key acronyms and quantitative information (maximum of 30 words / bullet point). Please use the passive voice. Please attach these in a separate file or send them by email, we will incorporate them accordingly.

Please also suggest a striking image or visual abstract to illustrate your article as a PNG file 550 px wide x 300-600 px high.

16) As part of the EMBO Publications transparent editorial process initiative (see our Editorial at http://embomolmed.embopress.org/content/2/9/329), EMBO Molecular Medicine will publish online a Review Process File (RPF) to accompany accepted manuscripts.
This file will be published in conjunction with your paper and will include the anonymous referee reports, your point-by-point response and all pertinent correspondence relating to the manuscript. Let us know whether you agree with the publication of the RPF and as here, if you want to remove or not any figures from it prior to publication.
Please note that the Authors checklist will be published at the end of the RPF.

EMBO Molecular Medicine has a "scooping protection" policy, whereby similar findings that are published by others during review or revision are not a criterion for rejection. Should you decide to submit a revised version, I do ask that you get in touch after three months if you have not completed it, to update us on the status.

I look forward to receiving your revised manuscript.

With kind regards,

Lise

Lise Roth, PhD
Editor
EMBO Molecular Medicine

The authors performed the requested changes.

16th May 2022

Dear Amin,

Thank you for submitting your revised manuscript to EMBO Molecular Medicine. I am pleased to inform you that we will be able to accept your manuscript once the following final editorial issues will be addressed:

1/ Main manuscript text:
- Please address the comments from our data editors and define the central band, boxes and whiskers of the boxplot in the figure legends for Fig. 6D, 7C, etc. Please also correct the legends for Fig 6B and EV4A (no SEM shown).
- Please remove the highlighted text and only keep in track changes mode any new modification.
- We can accommodate a maximum of 5 keywords, please adjust accordingly.
- Please remove "data not shown" (p. 9). As per our guidelines on "Unpublished Data", all data referred to in the paper should be displayed in the main or Expanded View figures.
- Please make sure that all figures are referenced in the main text and in chronological order (i.e. Fig. 1E is called out after Fig. 2A, B, and Fig. EV1B is called out after EV2A, B).
- Author contribution: please confirm that Paladd Asavarut, Sajee Waramit and Keittisak Suwan are co-first authors, with equal contribution to the manuscript.

2/ Thank you for providing The paper explained. I added minor modifications, mostly to shorten the text, please amend as you see fit:
Medical Issue
Since the 1980s, antitumor cytokines have been used as cancer immunotherapy. Yet a fundamental problem remains the control over immune activating cytokines at the target site, which can have fatal effects on the host. Cytokine-encoding genes have thus been developed to express cytokines in cancer cells only; however, gene delivery is hindered by the lack of tumor-selective vectors and issues linked to repeated administrations.

Results
We established a unique prokaryotic viral-based approach of intravenous gene delivery to specifically target tumors by using the filamentous M13 bacteriophage that infects bacteria only. In this vector, the M13 phage capsid was engineered to target cancer and deliver therapeutic transgene expression cassettes carrying genes encoding interleukin IL12, IL15 and tumor necrosis factor alpha. These phage-derived particles proved to be an efficient platform for safe and selective systemic delivery of cytokines to solid tumors, while avoiding healthy tissues in preclinical models of human and murine tumors. Moreover, administration of particles in immunocompetent animals could be repeated and resulted in tumor eradication and complete response in more than 50% of the mice.

Clinical Impact
The newly developed phage-derived particles can be applied for selective and efficient cytokine therapy. These findings are important since targeted cytokine delivery has been a major barrier for clinical translation. Given that cytokines have already been tested in cancer patients, and that phage safety in human is increasingly established, the clinical efficacy of this targeted cytokine therapy to treat solid tumors is promising. Moreover, the treatment is administered through the systemic route, and thus could be applied both for localised and metastatic disease.

3/ Thank you for providing a synopsis figure. Please upload it as a PNG/TIF/JPEG file 550 px wide x 300-600 px high, and make sure that the text remains legible.
The synopsis text should include a short stand first (maximum of 300 characters, including space) as well as 2-5 one-sentences bullet points that summarizes the paper. Please write the bullet points to summarize the key NEW findings. They should be designed to be complementary to the abstract - i.e. not repeat the same text. We encourage inclusion of key acronyms and quantitative information (maximum of 30 words / bullet point).

4/ As part of the EMBO Publications transparent editorial process initiative (see our Editorial at http://embomolmed.embopress.org/content/2/9/329), EMBO Molecular Medicine will publish online a Review Process File (RPF) to accompany accepted manuscripts.
This file will be published in conjunction with your paper and will include the anonymous referee reports, your point-by-point response and all pertinent correspondence relating to the manuscript. Let us know whether you agree with the publication of the RPF and as here, if you want to remove or not any figures from it prior to publication.

I look forward to receiving your revised manuscript.

With kind regards,

Lise

Lise Roth, PhD
Senior Editor
EMBO Molecular Medicine

The authors performed the requested changes.

24th May 2022

Dear Amin,

Thank you for submitting your revised files. I am pleased to inform you that your manuscript is accepted for publication and is now being sent to our publisher to be included in the next available issue of EMBO Molecular Medicine.

Congratulations on your interesting work!

With kind regards,

Lise

Lise Roth, Ph.D
Senior Editor
EMBO Molecular Medicine

Follow us on Twitter @EmboMolMed
Sign up for eTOCs at embopress.org/alertsfeeds

EMBO Press Author Checklist

Corresponding Author Name: Amin Hajitou	
Journal Submitted to: EMBO Molecular Medicine	
Manuscript Number: EMM-2021-15418	

USEFUL LINKS FOR COMPLETING THIS FORM
The EMBO Journal - Author Guidelines
EMBO Reports - Author Guidelines
Molecular Systems Biology - Author Guidelines
EMBO Molecular Medicine - Author Guidelines

Reporting Checklist for Life Science Articles (updated January 2022)
This checklist is adapted from Materials Design Analysis Reporting (MDAR) Checklist for Authors. MDAR establishes a minimum set of requirements in transparent reporting in the life sciences (see Statement of Task: 10.31222/osf.io/9sm4x). Please follow the journal's guidelines in preparing your manuscript.
Please note that a copy of this checklist will be published alongside your article.

Abridged guidelines for figures
1. Data
The data shown in figures should satisfy the following conditions:
- the data were obtained and processed according to the field's best practice and are presented to reflect the results of the experiments in an accurate and unbiased manner.
- ideally, figure panels should include only measurements that are directly comparable to each other and obtained with the same assay.
- plots include clearly labeled error bars for independent experiments and sample sizes. Unless justified, error bars should not be shown for technical replicates.
- if n<5, the individual data points from each experiment should be plotted. Any statistical test employed should be justified.
- Source Data should be included to report the data underlying figures according to the guidelines set out in the authorship guidelines on Data Presentation.

2. Captions
Each figure caption should contain the following information, for each panel where they are relevant:
- a specification of the experimental system investigated (eg cell line, species name).
- the assay(s) and method(s) used to carry out the reported observations and measurements.
- an explicit mention of the biological and chemical entity(ies) that are being measured.
- an explicit mention of the biological and chemical entity(ies) that are altered/varied/perturbed in a controlled manner.
- the exact sample size (n) for each experimental group/condition, given as a number, not a range;
- a description of the sample collection allowing the reader to understand whether the samples represent technical or biological replicates (including how many animals, litters, cultures, etc.).
- a statement of how many times the experiment shown was independently replicated in the laboratory.
- definitions of statistical methods and measures:
  - common tests, such as t-test (please specify whether paired vs. unpaired), simple χ2 tests, Wilcoxon and Mann-Whitney tests, can be unambiguously identified by name only, but more complex techniques should be described in the methods section;
  - are tests one-sided or two-sided?
  - are there adjustments for multiple comparisons?
  - exact statistical test results, e.g., P values = x but not P values < x;
  - definition of 'center values' as median or average;
  - definition of error bars as s.d. or s.e.m.

Please complete ALL of the questions below.
Select "Not Applicable" only when the requested information is not relevant for your study.

Materials

Newly Created Materials	Information included in the manuscript?	In which section is the information available? (Reagents and Tools Table, Materials and Methods, Figures, Data Availability Section)
New materials and reagents need to be available; do any restrictions apply?	Not Applicable	

Antibodies	Information included in the manuscript?	In which section is the information available? (Reagents and Tools Table, Materials and Methods, Figures, Data Availability Section)
For **antibodies** provide the following information:<br>- Commercial antibodies: RRID (if possible) or supplier name, catalogue number and or/clone number<br>- Non-commercial: RRID or citation	Yes	Materials and Methods

DNA and RNA sequences	Information included in the manuscript?	In which section is the information available? (Reagents and Tools Table, Materials and Methods, Figures, Data Availability Section)
**Short novel DNA or RNA including primers, probes**: provide the sequences.	Yes	Table EV1 and Table EV2

Cell materials	Information included in the manuscript?	In which section is the information available? (Reagents and Tools Table, Materials and Methods, Figures, Data Availability Section)
**Cell lines:** Provide species information, strain. Provide accession number in repository **OR** supplier name, catalog number, clone number, and/**OR** RRID.	Yes	Materials and Methods
**Primary cultures:** Provide species, strain, sex of origin, genetic modification status.	Yes	Materials and Methods
Report if the cell lines were recently **authenticated** (e.g., by STR profiling) and tested for mycoplasma contamination.	Yes	Materials and Methods

Experimental animals	Information included in the manuscript?	In which section is the information available? (Reagents and Tools Table, Materials and Methods, Figures, Data Availability Section)
**Laboratory animals or Model organisms:** Provide species, strain, sex, age, genetic modification status. Provide accession number in repository **OR** supplier name, catalog number, clone number, **OR** RRID.	Yes	Material and Methods
**Animal observed in or captured from the field:** Provide species, sex, and age where possible.	Not Applicable	
Please detail **housing and husbandry conditions**.	Yes	Materials and Methods

Plants and microbes	Information included in the manuscript?	In which section is the information available? (Reagents and Tools Table, Materials and Methods, Figures, Data Availability Section)
**Plants:** provide species and strain, ecotype and cultivar where relevant, unique accession number if available, and source (including location for collected wild specimens).	Not Applicable	
**Microbes:** provide species and strain, unique accession number if available, and source.	Yes	Materials and Methods

Human research participants	Information included in the manuscript?	In which section is the information available? (Reagents and Tools Table, Materials and Methods, Figures, Data Availability Section)
If collected and within the bounds of privacy constraints report on age, sex and gender or ethnicity for all study participants.	Not Applicable	

Core facilities	Information included in the manuscript?	In which section is the information available? (Reagents and Tools Table, Materials and Methods, Figures, Data Availability Section)
If your work benefited from core facilities, was their service mentioned in the acknowledgments section?	Not Applicable	

Design

Study protocol	Information included in the manuscript?	In which section is the information available? (Reagents and Tools Table, Materials and Methods, Figures, Data Availability Section)
If study protocol has been **pre-registered, provide DOI in the manuscript**. For clinical trials, provide the trial registration number **OR** cite DOI.	Not Applicable	
Report the **clinical trial registration number** (at ClinicalTrials.gov or equivalent), where applicable.	Not Applicable	

Laboratory protocol	Information included in the manuscript?	In which section is the information available? (Reagents and Tools Table, Materials and Methods, Figures, Data Availability Section)
Provide DOI OR other citation details if **external detailed step-by-step protocols** are available.	Not Applicable	

Experimental study design and statistics	Information included in the manuscript?	In which section is the information available? (Reagents and Tools Table, Materials and Methods, Figures, Data Availability Section)
Include a statement about **sample size** estimate even if no statistical methods were used.	Yes	Materials and Methods
Were any steps taken to minimize the effects of subjective bias when allocating animals/samples to treatment (e.g. **randomization procedure**)? If yes, have they been described?	Yes	Materials and Methods
Include a statement about **blinding** even if no blinding was done.	Yes	Materials and Methods
Describe **inclusion/exclusion criteria** if samples or animals were excluded from the analysis. Were the criteria pre-established?<br><br>If sample or data points were omitted from analysis, report if this was due to attrition or intentional exclusion and provide justification.	Yes	Materials and Methods
For every figure, are **statistical tests** justified as appropriate? Do the data meet the assumptions of the tests (e.g., normal distribution)? Describe any methods used to assess it. Is there an estimate of variation within each group of data? Is the variance similar between the groups that are being statistically compared?	Yes	Materials and Methods, and Figure Legends

Sample definition and in-laboratory replication	Information included in the manuscript?	In which section is the information available? (Reagents and Tools Table, Materials and Methods, Figures, Data Availability Section)
In the figure legends: state number of times the experiment was **replicated** in laboratory.	Yes	Figures Legends
In the figure legends: define whether data describe **technical or biological replicates**.	Yes	Figures Legends

Ethics

Ethics	Information included in the manuscript?	In which section is the information available? (Reagents and Tools Table, Materials and Methods, Figures, Data Availability Section)
Studies involving **human participants**: State details of **authority granting ethics approval** (IRB or equivalent committee(s), provide reference number for approval.	Not Applicable	
Studies involving **human participants**: Include a statement confirming that **informed consent** was obtained from all subjects and that the experiments conformed to the principles set out in the WMA Declaration of Helsinki and the Department of Health and Human Services Belmont Report.	Not Applicable	
Studies involving **human participants:** For publication of **patient photos**, include a statement confirming that consent to publish was obtained.	Not Applicable	
Studies involving experimental **animals**: State details of **authority granting ethics approval** (IRB or equivalent committee(s), provide reference number for approval. Include a statement of compliance with ethical regulations.	Yes	Materials and Methods
Studies involving **specimen and field samples:** State if relevant **permits** obtained, provide details of authority approving study; if none were required, explain why.	Not Applicable	

Dual Use Research of Concern (DURC)	Information included in the manuscript?	In which section is the information available? (Reagents and Tools Table, Materials and Methods, Figures, Data Availability Section)
Could your study fall under dual use research restrictions? Please check biosecurity documents and list of **select agents and toxins** (CDC): https://www.selectagents.gov/sat/list.htm	Not Applicable	
If you used a select agent, is the security level of the lab appropriate and reported in the manuscript?	Not Applicable	
If a study is subject to dual use research of concern regulations, is the name of the **authority granting approval and reference number** for the regulatory approval provided in the manuscript?	Not Applicable	

Reporting

The MDAR framework recommends adoption of discipline-specific guidelines, established and endorsed through community initiatives. Journals have their own policy about requiring specific guidelines and recommendations to complement MDAR.

Adherence to community standards	Information included in the manuscript?	In which section is the information available? (Reagents and Tools Table, Materials and Methods, Figures, Data Availability Section)
State if relevant guidelines or checklists (e.g., **ICMJE, MIBBI, ARRIVE, PRISMA**) have been followed or provided.	Yes	Materials and Methods, Figure Legends, Reagents, Tables EV1 and EV2.
For **tumor marker prognostic studies,** we recommend that you follow the **REMARK** reporting guidelines (see link list at top right). See author guidelines, under 'Reporting Guidelines'. Please confirm you have followed these guidelines.	Not Applicable	
For **phase II and III randomized controlled trials**, please refer to the **CONSORT** flow diagram (see link list at top right) and submit the CONSORT checklist (see link list at top right) with your submission. See author guidelines, under 'Reporting Guidelines'. Please confirm you have submitted this list.	Not Applicable	

Data Availability

Data availability	Information included in the manuscript?	In which section is the information available? (Reagents and Tools Table, Materials and Methods, Figures, Data Availability Section)
Have **primary datasets** been deposited according to the journal's guidelines (see 'Data Deposition' section) and the respective accession numbers provided in the Data Availability Section?	Not Applicable	
Were **human clinical and genomic datasets** deposited in a public access-controlled repository in accordance to ethical obligations to the patients and to the applicable consent agreement?	Not Applicable	
Are **computational models** that are central and integral to a study available without restrictions in a machine-readable form? Were the relevant accession numbers or links provided?	Not Applicable	
If publicly available data were reused, provide the respective **data citations in the reference list**.	Not Applicable	
